# Evaluation of Maltose-Based Cationic Liposomes with Different Hydrophobic Tails for Plasmid DNA Delivery

**DOI:** 10.3390/molecules22030406

**Published:** 2017-03-12

**Authors:** Bo Li, Liangliang Deng, Meiyan Liu, Youlin Zeng

**Affiliations:** National and Local Joint Engineering Laboratory for New Petrochemical Materials and Fine Utilization of Resources, Hunan Normal University, Changsha 410081, Hunan, China; bolilicn@gmail.com (B.L.); liangliangdeng1998@163.com (L.D.); xplmy@163.com (M.L.)

**Keywords:** maltose-based cationic lipid, synthesis, transfection assay

## Abstract

In this paper, three cationic glycolipids with different hydrophobic chains Malt-DiC12MA (**IX a**), Malt-DiC14MA (**IX b**) and Malt-DiC16MA (**IX c**) were constructed by using maltose as starting material via peracetylation, selective 1-*O*-deacetylation, trichloroacetimidation, glycosylation, azidation, deacetylation, Staudinger reaction, tertiary amination and quaternization. Target compounds and some intermediates were characterized by ^1^H-NMR, ^13^C-NMR, ^1^H-^1^H COSY and ^1^H-^13^C HSQC. The results of gel electrophoresis assay, atomic force microscopy images (AFM) and dynamic light scattering (DLS) demonstrate that all the liposomes could efficiently bind and compact DNA (N/P ratio less than 2) into nanoparticles with proper size (88 nm–146 nm, PDI < 0.4) and zeta potential (+15 mV–+26 mV). The transfection efficiency and cellular uptake of glycolipids in HEK293 cell were evaluated through the enhanced green fluorescent protein (EGFP) expression and Cy3-labeled pEGFP-C1 (Enhanced Green Fluorescent Protein plasmid) images, respectively. Importantly, it indicated that Malt-DiC14MA exhibited high gene transfer efficiency and better uptake capability at N/P ratios of 8:1. Additionally, the result of cell viability showed glycolipids exhibited low biotoxicity and good biocompatibility by thiazolyl blue tetrazolium bromide (MTT) assay.

## 1. Introduction

In recent years, gene therapy mediated by the transfer of genetic materials [1,2,3,4] (including small interfering RNA, oligonucleotides and plasmid DNA) to specific cells in order to down or unregulate the expression of defective genes, has achieved enormous attention due to its potential application in prevention and treatment of acquired and inherited diseases such as cancer, infectious diseases and immunodeficiency, etc. [5,6,7,8,9]. Nevertheless, one of the major impediments in the therapeutic potential of nucleic acids is the limited development of efficient and safe vectors which can protect genes from degradation, deliver genes to the specific cells and promote its endosomal escape with high efficiency and specificity [10,11,12]. Basically, there are two main forms of vectors for gene delivery: viral and non-viral vectors, each of them has their own advantages and disadvantages [13]. Virus-based vectors, such as adenovirus vectors, adeno-associated virus vectors, or retrovirus vectors, have attracted tremendous interests due to their high transfection efficiency at mediating the process of gene delivery [14,15,16]. However, they suffer from some shortcomings, such as immunogenicity, uncertainty safety, insertional mutagenesis and oncogenic transformation, which limit their application in clinical trials [17,18].

Non-viral vectors including cationic liposomes [1], positively charged polymers [19] and peptides [20,21] are widely studied due to their low immunogenicity and cytotoxicity, high gene carrying capacity, ease of large-scale preparation and potential to incorporate targeting ligands [22,23,24,25]. Cationic lipids, as one of the most promising non-viral gene delivery systems, have been intensively investigated for their efficient transfection and facile formulation [26]. After the first discovery of cationic liposomes, *N*-[1-(2,3-dioleyloxy)propyl]-*N*,*N*,*N*-trimethylammonium chloride (DOTMA) was reported by Felgner and Danielsen [27], many liposomal gene delivery reagents including monovalent cationic liposomes, multivalent cationic liposomes, pH-sensitive cationic liposomes and lipidoid nanoparticles [1] have also been developed. Among these cationic lipids, carbohydrates-based cationic lipids have been drawn great attention [28,29,30,31] because carbohydrates and their derivatives (such as, glycans, proteoglycans, glycoproteins, or glycolipids [32,33]) played key roles in a wide range of biological processes including cell-cell informational reception and transmission, cell growth and metastasis, cell adhesion intercellular identification and immunity [34,35,36]. Furthermore, lots of studies have shown that glycoside ligand could be acted as a targetable ligand for receptors, such as galactose, mannose, glucose and hyaluronic acid [30,31,37,38,39], which overexpressed in the surface of cancer cells. Therefore, carbohydrates may be conjugated in targeted gene delivery system with high transfer efficiency and safety.

**Scheme 1:**
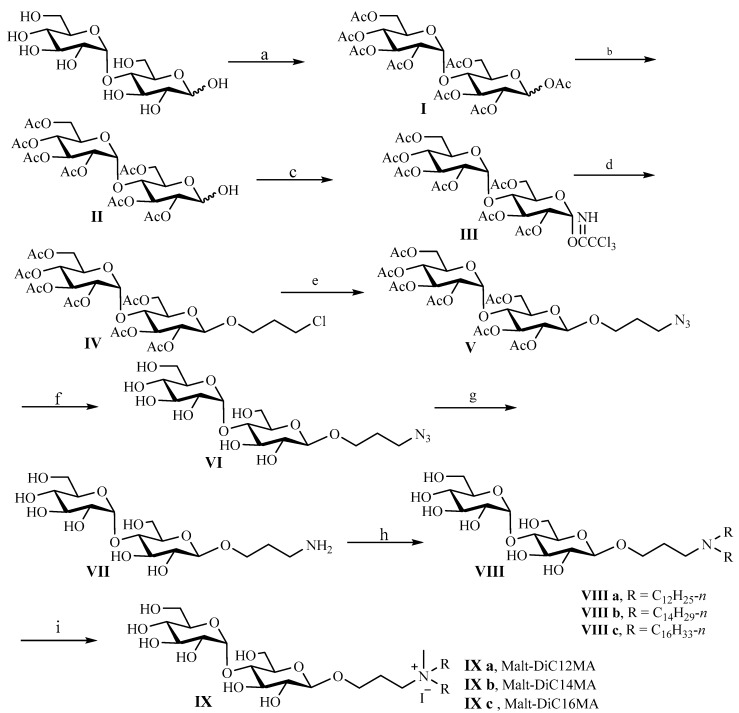
Synthesis of maltose-based cationic liposomes **IX a**–**c**. *Reagents and conditions*: (**a**) Ac_2_O, HClO_4_; (**b**) piperazine, THF (Tetrahydrofuran); (**c**) NCCCl_3_, K_2_CO_3_, CH_2_Cl_2_; (**d**) 3-chloro-1-propanol, TMSOTf (trimethylsilyl trifluoromethanesulfonate), CH_2_Cl_2_, −20 °C to RT; (**e**) NaN_3_, DMF (*N*,*N*-Dimethylformamide), 80 °C; (**f**) NH_3_/CH_3_OH; (**g**) Ph_3_P, THF, 75 °C, reflux; (**h**) K_2_CO_3_, alkyl bromide, C_2_H_5_OH, CH_3_OH, 70 °C; (**i**) CH_3_I, THF. Lipids **IX a**–**IX d** were labeled as Malt-DiC12MA, Malt-DiC14MA, Malt-DiC16MA, respectively.

In a previous study [37], we have demonstrated that glucose-based cationic lipids with hydrophobic tails linked to the positively charged nitrogen atom were higher than that linked to the sugar ring. To investigate the affection of sugar skeleton on the transfection efficiencies of sugar-based cationic lipids, in this work, three cationic lipids of Malt-DiC12MA (**IX a**), Malt -DiC14MA (**IX b**) and Malt-DiC16MA (**IX c**) (Scheme 1), with hydrophobic chains linked to the positively charged nitrogen atoms, were successfully synthesized and the corresponding structures were confirmed by ^1^H-NMR, ^13^C-NMR, ^1^H-^1^H COSY (2D-correlation spectroscopy) and ^1^H-^13^C HSQC (Heteronuclear Single Quantum Coherence). The physical properties and DNA binding capacity of these cationic liposomes and their complexes were characterized by dynamic light scattering (DLS), atomic force microscopy (AFM) and gel electrophoresis. Additionally, the in vitro transfection and cellular uptake of cationic liposomes was evaluated by using HEK293 cells (human embryonic kidney cells), and the toxicity of liposomes was studied by MTT (3-(4,5-dimethyl-2-thiazolyl)-2,5- diphenyl-2-*H*-tetrazolium bromide) assay.

## 2. Results and Discussions

### 2.1. Synthesis of Cationic Glycolipids and Their Structural Elucidation

The straightforward procedure for the synthesis of maltose-based glycolipids having cationic quaternary ammonium headgroup with different twin-alkyl chains is displayed in Scheme 1. The synthesis started with the maltose in the presence of acetic anhydride and perchloric acid to afford the compound **I** in moderate yields (74.0%) [40]. The intermediate **III** [41] was synthesized by selective 1-*O*-deacetylation of compound **I** with piperazine in tetrahydrofuran followed by trichloroacetimidation with trichloroacetonitrile in the presence of potassium carbonate in anhydrous dichloromethane. Glycosylation of intermediate **III** with 3-chloro-1-propanol was catalyzed by TMSOTf (trimethylsilyl trifluoromethanesulfonate) to yield compound **IV**, which was subsequently transformed into intermediate **VI** by azidation with sodium azide in *N*,*N*-dimethylformamide and deacetylation with saturated ammonia in methanol separately. Then, intermediate **VII** was constructed from compounds **VI** by the Staudinger reaction. The lipids **IX a**–**c** were synthesized by tertiary amination of compound **VII** with alkyl bromines in alcohol (methanol:ethanol = 5:2 in volume) followed by quaternization with iodomethane in tetrahydrofuran. The structures of some synthetic intermediates and glycolipids were confirmed by ^1^H-NMR, ^13^C-NMR, ^1^H-^1^H COSY and ^1^H-^13^C HSQC. The detailed synthetic processes are described in the Experimental Section and NMR spectra were included in the Supplementary Materials.

### 2.2. Characterization of Cationic Liposome

The average size, PDI (Polydispersity Index) distribution and zeta potential of nanoparticles were deemed to be important factors for gene delivery, because they markedly affected transfection efficacy, cell toxicity, cellular uptake and release of non-viral vectors [42,43,44]. Herein, these parameters of cationic liposomes and lipoplexes were measured by DLS run at 25 °C using He-Ne laser as laser source having a wavelength of 633 nm and incidence angle of 173 °C. As shown in Table 1, the naked liposomes showed positively charged between +36 and +47 mV and their average particle size at range from 60 nm to 110 nm. Strangely, as shown in Table 1, the average particle size of Malt-DiC14MA liposome is smaller than the other two liposomes (Malt-DiC12MA and Malt-DiC16MA), and one of the main reason may be that Malt-DiC14MA lipid easily forms the smaller micelles aggregate. After lipoplexes formed at different N/P ratios (N/P ratio is the molar ratio of nitrogen N (a representative of cationic liposomes) to phosphor P (a representative of DNA)), as showed in Table 2, the average size grows erratically at N/P ratio range from 4:1 to 8:1 (78 nm–98 nm) compared with naked liposomes. The surface zeta potential of the corresponding complexes increased with the increase of N/P ratio, which was, nevertheless, fewer than for the naked liposomes. In addition, all the naked liposomes and lipoplexes possess non-uniform size distribution (PDI < 0.4), and the average size and zeta potential of the lipoplexes are suitable for intracellular uptake and gene delivery [45,46].

**Figure 1:**
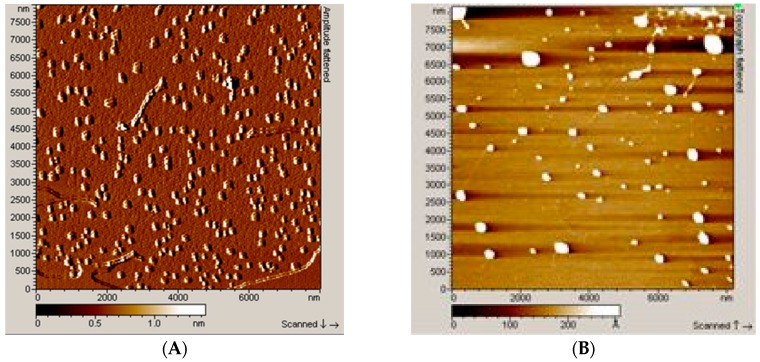
Atomic force microscopy (AFM) images morphologies images of the naked lipids Malt-DiC14MA (**A**) and its pDNA complexes (**B**).

Compared with DLS (in suspension), the information of particle size of the dried liposomes and liposome/DNA complexes achieved by AFM is not accurate, while they could give intuitively insight into the morphology of liposomes and lipoplexes. Although AFM is not the best method to demonstrate the morphology of the liposomes and lipoplexes compared with transmission electron microscopy (TEM) [47], liposome Malt-DiC14MA and corresponding lipoplexes at N/P ratio of 8:1 were characterized only by AFM. As shown in Figure 1, lipids Malt-DiC14MA were dispersed uniformity on the mica and the spherical particles were formed with a diameter of 100–200 nm (Figure 1A). After the lipoplexes were formed at an N/P ratio of 8:1, the morphology of the lipoplexes was irregular, with spherical nanoparticle diameters of approximately 100–350 nm (Figure 1B). Furthermore, the particle size of the complex was slightly larger than naked liposomes.

### 2.3. Gel Retardation Assay

**Figure 2:**
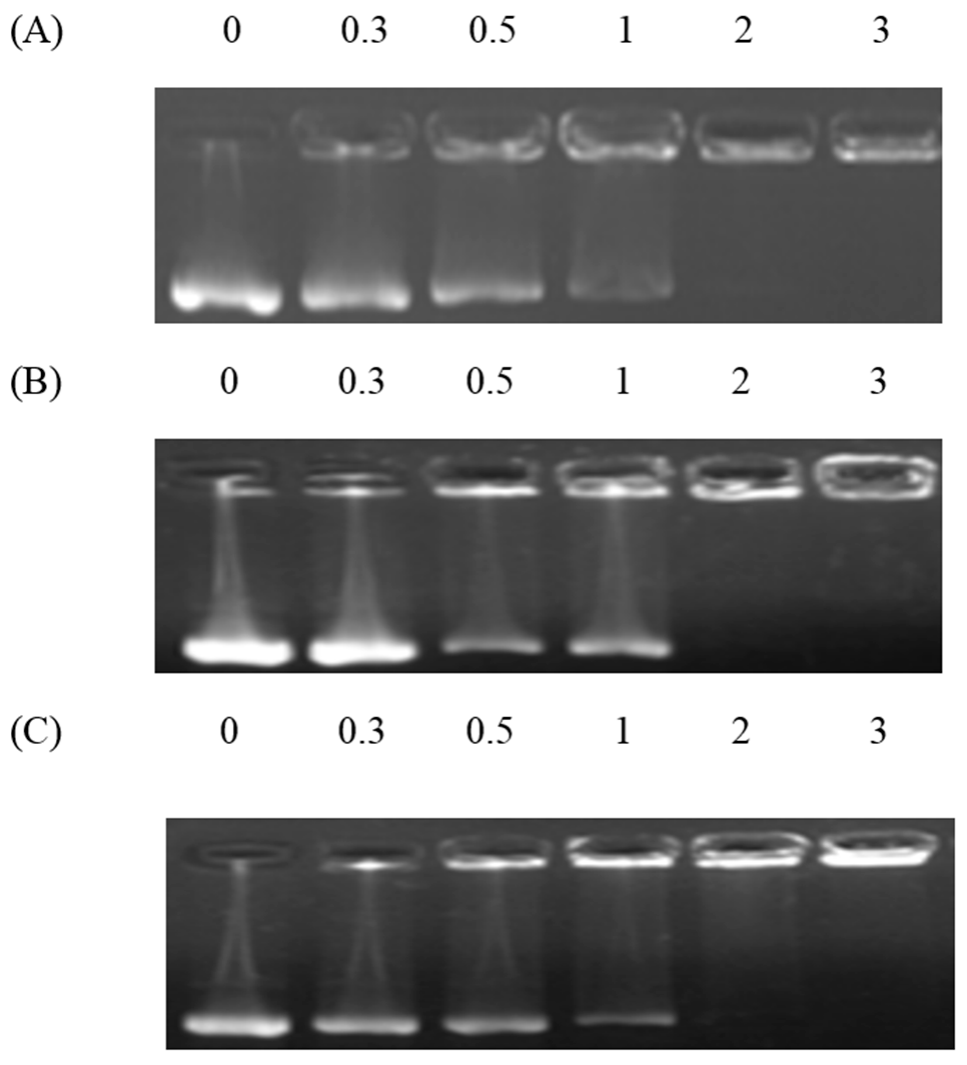
Gel electrophoresis assay of cationic liposome Malt-DiC12MA/DNA (**A**), Malt-DiC14MA/DNA (**B**); Malt-DiC16MA/DNA (**C**) complexes at five different N/P (lipids/DNA) ratios (0.3:1, 0.5:1, 1:1, 2:1, 3:1). The number 0 in the gel images represents the naked pDNA alone run in wells.

The ability of DNA binding was considered as an important parameter of cationic lipid-based gene delivery systems. To explore the interaction between cationic glycolipids and pDNA (plasmid DNA), a gel retardation assay after incubating the cationic liposome with a constant amount of pDNA at five different N/P ratios (0.3:1, 0.5:1, 1:1, 2:1, 3:1) was performed using the naked plasmid DNA (the number 0) as a control. As shown in Figure 2, cationic liposomes Malt-DiC12MA and Malt-DiC14MA were able to retard the pDNA completely from the well at N/P ratios of 1:1, whereas liposomes Malt-DiC16MA presented complete retardation at N/P ratios of 2:1. Based on the above description, the maltose-based glycolipids could bind DNA molecules efficiently and may be a useful gene carrier system.

### 2.4. Transfection

**Figure 3:**
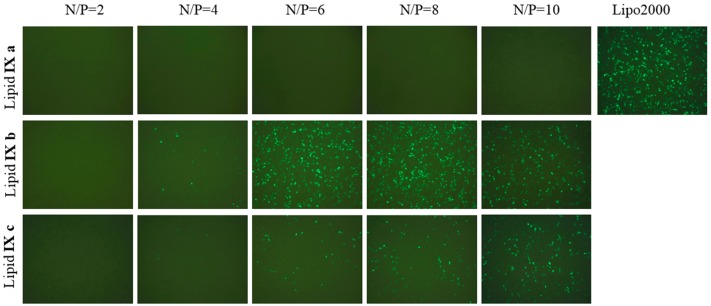
Fluorescence microscope images of HEK293 cells transfected by glycolipid/DNA complexes under different N/P ratios of 2:1, 4:1, 6:1, 8:1. 10:1.

Transfection efficiency is a key experimental parameter of cationic lipid based non-viral gene vector systems. As previously reported [48,49], the gene delivery efficiencies of cationic liposomes strongly depend on the molecular structure and liposomes/pDNA binding ratios. Herein, the transfection capacity of the maltose-based cationic liposomes was estimated by analyzing the expression of green fluorescent protein (GFP) in HEK293 cells at five N/P ratios (2:1, 4:1, 6:1, 8:1 and 10:1). As shown in Figure 3, the maximum transfection efficiency of Malt-DiC14MA lipoplexes was obtained at N/P ratio of 8:1, which is approximately equivalent to the positive control, Lipofectamine2000 (lipo2000). For Malt-DiC16MA liposome, the density of GFP cells increased with increase of N/P ratio but the fluorescent protein expression was weaker integrally than Malt-DiC14MA lipoplexes. However, the transfection efficiency of glucose-based lipids with the hydrophobic tails (C14 or C16) on the positively charged nitrogen atoms were as good as or better than lipo2000 [37]. Simultaneously, the lack of transfections of Malt-DiC12MA liposomes under different N/P ratios and the other two liposomes (Malt-DiC14MA and Malt-DiC16MA) at N/P of 2:1 were also observed, which corresponded with the glucose-based cationic liposomes with hydrophobic tails (C12) at all the N/P ratios and (C14 and C16) at low N/P ratios.

The difference of transfection efficiencies between the maltose-based cationic liposomes with similar structures were attributed primarily to various parameters such as particles sizes (Table 2), DNA binding ability (Figure 2) and the length of hydrophobic chains (Scheme 1), which together affect the processes of cellular uptake and intracellular release. According to the results of pEGFP-C1 (Enhanced Green Fluorescent Protein plasmid) transfection, the length of hydrophobic alkane chains might play a major role in the transfection process, which has an influence on the process of liposome formation and lipoplex-membrane fusion.

### 2.5. Cellular Uptake

**Figure 4:**
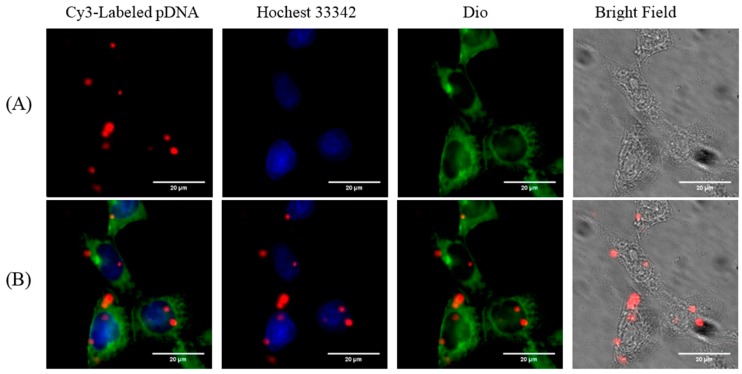
Fluorescence microscopic images (100×) of cellular uptake of Malt-DiC14MA Lipids/DNA complexes at the N/P ratio of 8:1 in HEK293 after 4 h of gene transfection. The upper column (**A**) represents single fluorescent images, the down column (**B**) represents merger images. Bar: 20 μm. (Green: Dio label cytomembrane, Red: Cy3-labeled pDNA, Blue: Hochest 33342 stained cell nuclei).

To further explore the correlation between cellular uptake and transfection efficiencies, the cellular uptake of the Malt-DiC12MA and Malt-DiC14MA liposomes/Cy3-labeled pDNA lipoplexes (Cy3, CyDye mono-reactive NHS Esters) were analyzed qualitatively in HEK293 cells at N/P charge ratio of 8:1. Dio (3,3’-dioctadecyloxacarbocyanine perchlorate) was used to stain the cytomembrane (green), the nuclei were labeled by Hochest 33342 (blue), Cy3-labeled pDNA (red) was used to trace the lipoplexes. As shown in Figure 4, the Malt-DiC14MA liposome -associated red fluorescent pDNA is clearly distributed on the periphery of cell nuclei (blue) and in the cell cytoplasm (green). However, the Malt-DiC12MA liposome is unable to bring pDNA into cells by membrane fusion or endocytosis (the red Cy3-labeled pDNA was not seen, Figure S1). The results demonstrated that the higher cellular uptake capability may be one of the important parameters at pEGFP expression.

### 2.6. Cytotoxicity Assay

**Figure 5:**
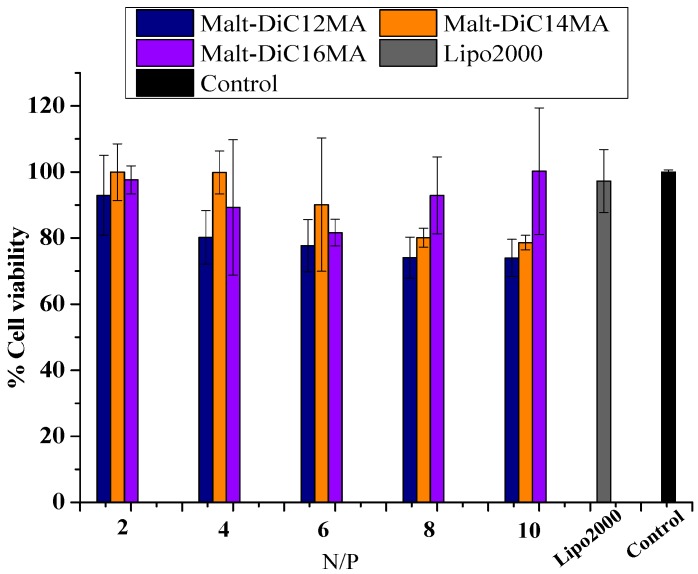
Representative percent cell viabilities of glycolipid/DNA complexes performed 4 h in HEK293 cells using MTT (thiazolyl blue tetrazolium bromide) assay. The cytotoxicity of Lipofectamine2000/pDNA and untreated cells were detected as the positive and normalized control, respectively. (Error bars indicate Standard Deviation, *n* = 3).

Apart from transfection efficiencies and cellular uptake, cytotoxicity is also an important factor in gene delivery protocols to deal with generating formulation about biocompatibility. Thus, the cytotoxicity of Malt-DiC12MA, Malt-DiC14MA and Malt-DiC16MA liposomes at various N/P ratios in HEK293 cells was assessed by MTT assay. Lipofectamine2000/pDNA and untreated cells were detected as the positive and normalized control, respectively. As demonstrated in Figure 5, the cytotoxicity of Malt-DiC12MA and Malt-DiC14MA lipoplexes were increased with the increase of the N/P ratios and the Malt-DiC12MA lipoplexes showed lower cell viability than Malt-DiC14MA and Malt-DiC16MA lipoplexes. Fortunately, the cell viability of all cationic liposomes was more than 75%, indicating that maltose-based cationic liposomes as gene delivery vectors present low cytotoxicity and good biocompatibility.

## 3. Experimental Section

### 3.1. Reagents and General Information

Maltose was purchased from Sinopharm Chemical Reagent Co. Ltd. (Shanghai, China). Lauryl bromide, myristyl bromide, cetyl bromide, 3-chloro-1-propanol, iodomethane were obtained from Shanghai Bangcheng Chemical Co. Ltd. (Shanghai, China). Triphenylphosphine was purchased from Hunan Huihong Chemical Regent Co. Ltd. (Changsha, China). Column chromatography was performed using 200–300 mesh silica gel. Ethidium bromide and agarose were purchased from Sangon Biotech (Shanghai, China). Dulbecco’s modified Eagle’s medium (DMEM), Opti-MEM and Fetal bovine serum (FBS) were purchased from GIBCO (Gaithersburg, MD, USA). Lipofectamine2000 was obtained from Invitrogen (Carlsbad, CA, USA). Label IT Tracker intracellular nucleic acid localization kit including TransIT-LT1 transfection reagent was purchased from Mirus Bio LLC (Madison, WI, USA). Hochest 33342 and Dio were purchased from the Beyotime Institute of Biotechnology (Haimen, China). Dimethyl sulfoxide (DMSO) and MTT were purchased from Sigma-Aldrich (St. Louis, MO, USA). Hek293 cells and EGFP-C_1_ pDNA were donated from School of Life Sciences, Hunan Normal University (Changsha, China). Cultivation plates (96-well and 24-well) and 50 mL cell cultivation flasks were purchased from Corning Co. Ltd. (Corning, NY, USA). In this work, all other reagents and solvent were of analytical grade and were used without further purification. The structures of some synthetic intermediates and final lipids were confirmed by ^1^H-NMR, ^1^H-^1^H COSY, ^1^H-^13^C HSQC (Bruker 500 MHz) and ^13^C-NMR (Bruker 125 MHz) (Bruker, Karlsruhe, Germany). HRMS was run on an UHPLC-Q-Tof Xevo-G2-XS instrument (Waters, Milford, MA, USA).

#### 3.1.1. Synthesis of Cationic Glycolipids **IX a** (Malt-DiC12MA)

*2,3,4,6-Tetra-O-acetyl-α-d-glucopyanosyl-(1→4)-1,2,3,6-tetra-O-acetyl-α,β-d-glucopyranose* (**I**). Acetic anhydride (200.0 mL, 2.1 mol) was cooled to 0 °C and then perchloric acid (0.5 mL) was added dropwise. Maltose (60.0 g, 0.18 mol) was added in partition under the temperature was not excess 20 °C. The reaction mixture was stirring until TLC (Thin Layer chromatography) (petroleum ether:ethyl acetate = 2:1) showed the reaction was completed, during which time the temperature was gradually raised to ambient temperature. The reaction mixture was washed with water and CH_2_Cl_2_ for three times. The organic layer was then dried over anhydrous Na_2_SO_4_ and concentrated to dryness to give compound **I** (88.0 g, 74.0%) as a white powder solid.

*2,3,4,6-Tetra-O-acetyl-α-d-glucopyranosyl-(1→4)-2,3,6-tri-O-acetyl-α,β-d-glucopyranose* (**II**). Piperidine (12.1 g, 140.5 mmol) was added slowly to a mixture of compound **I** (87.7 g, 129.2 mmol) and THF (Tetrahydrofuran) (530.0 mL). The mixture was stirring at room temperature until TLC (petroleum ether:ethyl acetate = 1:1) indicated the substrate was almost disappeared. The mixture was concentrated, then purified by silica gel column chromatography (petroleum ether:ethyl acetate = 2:1) to give compound **II** (74.6 g, 90.6%) as a white powder solid.

*2,3,4,6-Aetra-O-acetyl-α-d-glucopyranosyl-(1→4)-2,3,6-tri-O-acetyl-α,β-d-glucopyanosyl trichloro-acetimidate* (**III**). Anhydrous K_2_CO_3_ (5.0 g, 56.1 mmol) and trichloroacetonitrile (8.8 mL, 87.6 mmol) were added to the solution of compound **II** (18.6 g, 29.2 mmol) in dry DCM (Dichloromethane, 200.0 mL). The mixture was stirring at room temperature until TLC (petroleum ether:ethyl acetate = 1:1) showed the starting material was disappeared. The mixture was filtered and concentrated to dryness. The residue was purified by silica gel column chromatography with petroleum ether:ethyl acetate = 2:1 as the eluent to give compound **III** (15.9 g, 69.7%) as a white powder solid.

*3′-Chloropropyl 2,3,4,6-tetra-O-acetyl-α-d-glucopyranosyl-(1→4)-2,3,6-tri-O-acetyl-β-d-glucopyanoside* (**IV**). TMSOTf (75.0 μL, 0.41 mmol) was added dropwise to a mixture of compound **III** (15.9 g, 20.4 mmol) and 1-chloro-3-hydroxypropane (4.2 mL, 61.2 mmol) in anhydrous CH_2_Cl_2_ (200.0 mL) at −20 °C. The ice bath was removed and the reaction mixture was stirred at room temperature. The completion of the reaction was monitored by TLC (petroleum ether:ethyl acetate = 2:1). The reaction mixture was washed with water and CH_2_Cl_2_. The organic phase was dried over anhydrous Na_2_SO_4_ and concentrated to dryness. The residue was purified by flash chromatography on silica gel (petroleum ether:ethyl acetate = 5:1) to give compound **IV** (11.9 g, 82.1%). ^1^H-NMR (CDCl_3_): δ (ppm): 5.32 (d, 1 H, *J*_1′,2′_ = 4.0 Hz, H-1′), 5.27 (dd, 1 H, *J*_3′,2′_ = 10.5 Hz, *J*_3′,4′_ = 9.5 Hz, H-3′), 5.18 (dd, 1 H, *J*_3,2_ = 9.0 Hz, *J*_3,4_ = 9.5 Hz, H-3), 4.97 (dd, 1 H, *J*_4′,5′_ = 10.5 Hz, *J*_4′,3′_ = 9.5 Hz, H-4′), 4.79–4.76 (dd, 1 H, *J*_2′,1′_ = 4.0 Hz, *J*_2′,3′_ = 10.5 Hz, H-2′), 4.75–4.71 (dd, 1 H, H-2, *J*_3,2_ = 9.0 Hz, *J*_2,1_ = 8.0 Hz, H-2), 4.46 (d, 1 H, *J*_1,2_ = 8.0 Hz, H-1), 4.42 (dd, 1 H, *J*_6a,6b_ = 2.0 Hz, *J*_6a,5_ = 12.0 Hz, H-6a), 4.19–4.14 (m, 2 H, H-6b, H-6a′), 4.00–3.86 (m, 4 H, H-6b′, H-4, H-5′, OCH_2_CH_2_CH*H*Cl), 3.63–3.60 (m, 2 H, H-5, OCH_2_CH_2_CH*H*Cl), 3.54–3.49 (m, 2 H, OC*H*_2_CH_2_CH_2_Cl), 2.06–1.92 (m, 23 H, 7 C*H*_3_CO, OCH_2_C*H*_2_CH_2_Cl); ^13^C-NMR (CDCl_3_): δ (ppm): 170.4 (2 C, 2 CH_3_*C*O), 170.3 (1 C, CH_3_*C*O), 170.0 (1 C, CH_3_*C*O), 169.8 (1 C, CH_3_*C*O), 169.5 (1 C, CH_3_*C*O), 169.3 (1 C, CH_3_*C*O), 100.3 (1 C, C-1), 95.3 (1 C, C-1′), 75.1 (1 C, C-3), 72.6, 72.0, 71.9, 69.8, 69.1, 68.3, 67.9, 66.2, 61.4, 59.0 (10 C, C-2, C-2′, C-3′, C-4′, C-4, C-5, C-5′, C-6, C-6′, OCH_2_CH_2_*C*H_2_Cl), 41.1 (1 C, O*C*H_2_CH_2_CH_2_Cl), 32.0 (1 C, OCH_2_*C*H_2_CH_2_Cl), 20.7, 20.6, 20.5, 20.4, 20.4, 20.4 (7 C, 7 *C*H_3_CO).

*3′-Azidopropyl 2,3,4,6-tetra-O-acetyl-α-d-glucopyranosyl-(1→4)-2,3,6-tri-O-acetyl-β-d-glucopyanoside* (**V**). Sodium azide (9.4 g, 144.6 mmol) was added slowly to a solution of compound **IV** (17.2 g, 24.1 mmol) in anhydrous DMF (*N*,*N*-Dimethylformamide) (200.0 mL) under stirring. Then the reaction mixture was refluxed at 75 °C for 24 h. After the completion of the reaction (monitored by TLC using petroleum ether:ethyl acetate = 3:2), the reaction mixture was extracted with water and CH_2_Cl_2_. The organic phase was dried over anhydrous Na_2_SO_4_ and concentrated to dryness. The residue was purified by flash chromatography on silica gel (petroleum ether:ethyl acetate = 2:1) to give compound **V** (13.2 g, 76.3%) as a white solid. ^1^H-NMR (CDCl_3_): δ (ppm): 5.34 (d, 1 H, *J*_1′,2′_ = 4.0 Hz, H-1′), 5.29 (dd, 1 H, *J*_3′,2′_ = 10.5 Hz, *J*_3′,4′_ = 10.0 Hz, H-3′), 5.18 (dd, 1 H, *J*_3,2_ = 9.0 Hz, *J*_3,4_ = 9.0 Hz, H-3), 4.98 (dd, 1 H, *J*_4′,5′_ = 9.5 Hz, *J*_4′,3′_ = 10.0 Hz, H-4′), 4.80–4.77 (dd, 1 H, *J*_2′,1′_ = 4.0 Hz, *J*_2′,3′_ = 10.5 Hz, H-2′), 4.77–4.73 (dd, 1 H, H-2, *J*_3,2_ = 9.0 Hz, *J*_2,1_ = 8.0 Hz, H-2), 4.46 (d, 1 H, *J*_1,2_ = 8.0 Hz, H-1), 4.43 (dd, 1 H, *J*_6a,6b_ = 2.5 Hz, *J*_6a,5_ = 12.0 Hz, H-6a), 4.20–4.14 (m, 2 H, H-6b, H-6a′), 3.97–3.83 (m, 4 H, H-6b′, H-4, H-5′, OCH_2_CH_2_CH*H*N_3_), 3.64–3.60 (m, 1 H, H-5), 3.56–3.51 (m, 1 H, OCH_2_CH_2_CH*H*N_3_), 3.32–3.25 (m, 2 H, OC*H*_2_CH_2_CH_2_N_3_), 2.08–1.92 (m, 21 H, 7 C*H*_3_CO), 1.85–1.71 (m, 2 H, OCH_2_C*H*_2_CH_2_N_3_); ^13^C-NMR (CDCl_3_): δ (ppm): 170.3 (3 C, 2 CH_3_*C*O), 170.0 (1 C, CH_3_*C*O), 169.7 (1 C, CH_3_*C*O), 169.4 (1 C, CH_3_*C*O), 169.2 (1 C, CH_3_*C*O), 100.1 (1 C, C-1), 95.4 (1 C, C-1′), 75.1 (1 C, C-3), 72.6, 72.0, 71.9, 69.8, 69.1, 68.3, 67.9 (7 C, C-5, C-5′, C-2, C-2′, C-3′, C-4′, C-4), 66.3 (1 C, OCH_2_CH_2_*C*H_2_N_3_), 62.6, 61.4 (2 C, C-6, C-6′), 44.8 (1 C, O*C*H_2_CH_2_CH_2_N_3_), 28.8 (1 C, OCH_2_*C*H_2_CH_2_N_3_), 20.7, 20.6, 20.5, 20.4, 20.4, 20.4 (7 C, 7 *C*H_3_CO).

*3′-Azidopropyl α-d-glucopyranosyl-(1→4)-β-d-glucopyanoside* (**VI**). Ammonia was bubbled into a solution of compound **V** (4.6 g, 6.4 mmol) in anhydrous methanol (50.0 mL) for 0.5 h. The reaction mixture was stirring at room temperature until TLC (petroleum ether:ethyl acetate 3:1) indicated that the reaction was completed. The mixture was concentrated, then purified by flash chromatography on silica gel (ethyl acetate:methanol = 15:1) to give compound **VI** (2.3 g, 85.2%) as a white powder solid. ^1^H-NMR (D_2_O): δ (ppm): 5.42 (d, 1 H, *J*_1′,2′_ = 4.0 Hz, H-1′), 4.49 (d, 1 H, *J*_1,2_ = 8.0 Hz, H-1), 4.04–3.99 (m, 1 H, OCH_2_CH_2_CH*H*N_3_), 3.97–3.94 (dd, 1 H, *J*_6a,6b_ = 2.0 Hz, *J*_6a,5_ = 12.0 Hz, H-6a), 3.89–3.86 (dd, 1 H, *J*_6a′,6b′_ = 2.0 Hz, *J*_6a′,5′_ = 12.0 Hz, H-6a′), 3.81–3.58 (m, 9 H, H-6b, H-6b′, H-4, H-5′, H-3, H-3′, H-2′, H-5, OCH_2_CH_2_CH*H*N_3_), 3.50–3.41 (m, 3 H, OC*H*_2_CH_2_CH_2_N_3_, H-4′), 3.34–3.30 (dd, 1 H, *J*_2,1_ = 8.0 Hz, *J*_2,3_ = 9.5 Hz, H-2), 1.96–1.91 (m, 2 H, OCH_2_C*H*_2_CH_2_N_3_); ^13^C-NMR (D_2_O): δ (ppm): 102.2 (1 C, C-1), 99.6 (1 C, C-1′), 76.9, 76.2, 74.6, 73.0, 72.9, 72.7, 71.7, 69.4 (8 C, C-5, C-5′, C-2, C-2′, C-3′, C-3, C-4′, C-4), 67.3 (1 C, OCH_2_CH_2_*C*H_2_N_3_), 60.8, 60.5 (2 C, C-6, C-6′), 47.9 (1 C, O*C*H_2_CH_2_CH_2_N_3_), 28.3 (1 C, OCH_2_*C*H_2_CH_2_N_3_).

*3′-Aminopropyl α-d-glucopyranosyl-(1→4)-β-d-glucopyanoside* (**VII**). PPh_3_ (1.8 g, 6.8 mmol) was added in portion to a solution of compound **VI** (1.9 g, 4.5 mmol) in THF (10.0 mL) and H_2_O (2.0 mL). The mixture was refluxed at 75 °C until TLC (ethyl acetate: methanol = 5:1) showed the starting material was disappeared. The reaction mixture was evaporated to dryness, then H_2_O (4 mL) was added to give a solid-liquid mixture, which was subsequently filtered and the filter was evaporated to dryness to give a colorless syrup **VII** (1.7 g, 94.4%) using as the starting materials for the next step directly without any purification.

*3′-(N,N-Didodecylamino)propyl α-d-glucopyranosyl-(1→4)-β-d-glucopyanoside* (**VIII a**). The mixture of compound **VII** (440.0 mg, 1.1 mmol), 1-bromododecane (1.1 g, 4.4 mmol, 1.0 mL), anhydrous K_2_CO_3_ (100.0 mg), CH_3_OH (10.0 mL), CH_3_CH_2_OH (4.0 mL) was refluxed until TLC (ethyl acetate:methanol = 3:1) showed the reaction was completed. The mixture was diluted with DCM (20.0 mL), washed with water for two times. The organic phase was dried by anhydrous Na_2_SO_4_ and concentrated to dryness. The residue was purified by silica gel column chromatography with ethyl acetate:methanol = 5:1 as the eluent to give compound **VIII a** (350.0 mg, 43.2%) as a white solid. ^1^H-NMR (MeOD, Methanol-D4): δ (ppm): 5.19 (d, 1 H, *J*_1′,2′_ = 3.5 Hz, H-1′), 4.39 (d, 1 H, *J*_1,2_ = 8.0 Hz, H-1), 4.07–4.02 (m, 1 H, OCH_2_CH_2_CH*H*N(CH_2_CH_2_(CH_2_)_9_CH_3_)_2_), 3.94–3.42 (m, 11 H, H-6a′, H-6b′, H-6a, H-6b, H-3′, OCH_2_CH_2_CH*H*N(CH_2_CH_2_(CH_2_)_9_CH_3_)_2_, H-3, H-5, H-4′, H-2′, H-5′), 3.41–3.35 (m, 2 H, OC*H*_2_CH_2_CH_2_N(CH_2_CH_2_(CH_2_)_9_CH_3_)_2_), 3.31–3.24 (m, 2 H, H-4, H-2), 3.22–3.16 (m, 4 H, N(C*H*_2_CH_2_(CH_2_)_9_CH_3_)_2_), 2.17–2.03 (m, 2 H, OCH_2_C*H*_2_CH_2_N(CH_2_CH_2_(CH_2_)_9_CH_3_)_2_, 1.78–1.71 (m, 4 H, N(CH_2_C*H*_2_(CH_2_)_9_CH_3_)_2_), 1.41–1.31 (m, 36 H, N(CH_2_CH_2_(C*H*_2_)_9_CH_3_)_2_), 0.92 (t, 6 H, *J* = 7.0 Hz, N(CH_2_CH_2_(C_9_H_18_)C*H*_3_)_2_); ^13^C-NMR (MeOD): δ (ppm): 104.1 (1 C, C-1), 102.8 (1 C, C-1′), 81.2, 77.7, 76.6, 74.9, 74.7, 74.5, 74.0, 71.5 (8 C, C-4′, C-5, C-5′, C-2, C-2′, C-3′, C-3, C-4), 68.4 (1 C, OCH_2_CH_2_*C*H_2_N(CH_2_CH_2_(CH_2_)_9_CH_3_)_2_, 62.7, 62.0 (2 C, C-6, C-6′), 54.3 (1 C, N(*C*H_2_CH_2_(CH_2_)_9_CH_3_)_2_), 52.7 (1 C, O*C*H_2_CH_2_CH_2_N(CH_2_CH_2_(CH_2_)_9_CH_3_)_2_, 33.0, 30.7, 30.6, 30.5, 30.4, 30.2, 27.6, 25.3, 24.7, 23.7 (21 C, some signals were overlapped, N(CH_2_(*C*H_2_)_10_CH_3_)_2_), OCH_2_*C*H_2_CH_2_N(CH_2_CH_2_(C_9_H_18_)CH_3_)_2_), 14.4, 14.4 (2 C, N(CH_2_(CH_2_)_10_*C*H_3_)_2_).

*3′-(N,N-Didodecyl-N-methylaminonium iodine)propyl α-d-glucopyranosyl-(1→4)-β-d-glucopyranoside* (**IX a**). CH_3_I (79.0 mg, 0.56 mmol, 35.0 µL) was added in portion to a mixture of compound **VIII a** (200.0 mg, 271.7 µmol) in THF (5.0 mL). The mixture was stirring at room temperature until TLC (ethyl acetate:methanol = 3:1) showed the reaction was almost completed. The reaction mixture was evaporated to dryness and a solid was precipitated when acetone was dropped to the syrup in an ice bath. The mixture was filtered, and the filtrate was washed with acetone and dried by vacuum to give **IX a** (20.0 g, 16.8%) as a light yellow solid. ^1^H-NMR (MeOD): δ (ppm): 5.19 (d, 1 H, *J*_1′,2′_ = 4.0 Hz, H-1′), 4.34 (d, 1 H, *J*_1,2_ = 8.0 Hz, H-1), 4.00–3.41 (m, 15 H, H-6a′, H-6b′, H-6a, H-6b, H-3′, OC*H*_2_CH_2_CH_2_N(CH_2_CH_2_(CH_2_)_9_ CH_3_)_2_, 2 OCH_2_CH_2_CH*H*N(CH_2_CH_2_(CH_2_)_9_CH_3_)_2_, H-3, H-5, H-4′, H-4, H-2, H-5′), 3.34–3.24 (m, 5 H, H-2, N(C*H*_2_CH_2_(CH_2_)_9_CH_3_)_2_), 3.07 (s, 1 H, (C*H*_3_)N(CH_2_(CH_2_)_10_CH_3_)_2_), 2.09–2.05 (m, 2 H, OCH_2_C*H*_2_CH_2_N(CH_2_CH_2_(CH_2_)_9_CH_3_)_2_, 1.82–1.71 (m, 4 H, N(CH_2_C*H*_2_(CH_2_)_9_CH_3_)_2_), 1.43–1.31 (m, 36 H, N(CH_2_CH_2_(C*H*_2_)_9_CH_3_)_2_), 0.92 (t, 6 H, *J* = 7.0 Hz, N(CH_2_CH_2_(C_9_H_18_)C*H*_3_)_2_); ^13^C-NMR (MeOD): δ (ppm): 104.4 (1 C, C-1), 103.0 (1 C, C-1′), 81.4, 77.9, 76.7, 75.1, 74.8, 74.7, 74.2, 71.6, 67.3, 62.9, 62.8, 62.2, 60.8 (14 C, C-4′, C-5, C-5′, C-2, C-2′, C-3′, C-3, C-4, OCH_2_CH_2_*C*H_2_ N(CH_2_CH_2_(CH_2_)_9_CH_3_)_2_, C-6, C-6′, N(*C*H_2_CH_2_(CH_2_)_9_CH_3_)_2_, O*C*H_2_CH_2_CH_2_N(CH_2_CH_2_(CH_2_)_9_CH_3_)_2_, 49.0 (1 C, (*C*H_3_)N(C_12_H_25_)_2_), 33.0, 30.7, 30.6, 30.5, 30.4, 30.2, 27.4, 24.2, 23.7, 23.2 (21 C, some signals were overlapped, N(CH_2_(*C*H_2_)_10_CH_3_)_2_), OCH_2_*C*H_2_CH_2_N(CH_2_CH_2_(C_9_H_18_)CH_3_)_2_), 14.4, 14.4 (2 C, N(CH_2_(CH_2_)_10_*C*H_3_)_2_). HRMS (high-resolution Mass Spectroscopy): *m*/*z* = 751.6287, in agreement with the calculated mass for [M]^+^ = C_40_H_80_NO_11_^+^.

#### 3.1.2. Synthesis of Cationic Glycolipids **IX b** (Malt-DiC14MA)

*3′-(N,N-Ditetradecylamino)propyl α-d-glucopyranosyl-(1→4)-β-d-glucopyranoside* (**VIII b**). The mixture of compound **VII** (900.0 mg, 2.3 mmol), 1-bromotetradecane (2.4 g, 8.8 mmol, 2.6 mL), anhydrous K_2_CO_3_ (0.20 g), CH_3_OH (10.0 mL), CH_3_CH_2_OH (4.0 mL) was refluxed at 75 °C until TLC (ethyl acetate: methanol = 5:1) showed the reaction was completed. The mixture was diluted with DCM (20.0 mL), washed with water for two times. The organic phase was dried by anhydrous Na_2_SO_4_ and concentrated to dryness. The residue was purified by silica gel column chromatography with ethyl acetate:methanol = 3:1 as the eluent to give compound **VIII b** (0.46 g, 25.6%) as a white powder. ^1^H-NMR (MeOD): δ (ppm): 5.21 (d, 1 H, *J*_1′,2′_ = 3.5 Hz, H-1′), 4.41 (d, 1 H, *J*_1,2_ = 8.0 Hz, H-1), 4.09–4.04 (m, 1 H, OCH_2_CH_2_CH*H*N(CH_2_CH_2_(CH_2_)_11_CH_3_)_2_), 3.97–3.93 (dd, 1 H, *J*_6a′,6b′_ = 2.0 Hz, *J*_6a′,5′_ = 5.0 Hz, H-6a′), 3.88–3.46 (m, 10 H, H-6b′, H-6a, H-6b, H-3′, OCH_2_CH_2_CH*H*N(CH_2_CH_2_(CH_2_)_11_CH_3_)_2_, H-3, H-5, H-4′, H-2′, H-5′), 3.44–3.36 (m, 2 H, OC*H*_2_CH_2_CH_2_N(CH_2_CH_2_(CH_2_)_11_CH_3_)_2_), 3.33–3.25 (m, 2 H, H-4, H-2), 3.25–3.18 (m, 4 H, N(C*H*_2_CH_2_(CH_2_)_11_CH_3_)_2_), 2.15–2.07 (m, 2 H, OCH_2_C*H*_2_CH_2_N(CH_2_CH_2_(CH_2_)_11_CH_3_)_2_, 1.83–1.73 (m, 4 H, N(CH_2_C*H*_2_(CH_2_)_11_CH_3_)_2_), 1.44–1.30 (m, 44 H, N(CH_2_CH_2_(C*H*_2_)_11_CH_3_)_2_), 0.94 (t, 6 H, *J* = 7.0 Hz, N(CH_2_CH_2_(C_11_H_22_)C*H*_3_)_2_); ^13^C-NMR (MeOD): δ (ppm): 104.1 (1 C, C-1), 102.8 (1 C, C-1′), 81.2, 77.7, 76.6, 74.9, 74.7, 74.5, 74.0, 71.5 (8 C, C-4′, C-5, C-5′, C-2, C-2′, C-3′, C-3, C-4), 68.4 (1 C, OCH_2_CH_2_ *C*H_2_N(CH_2_CH_2_ (CH_2_)_11_CH_3_)_2_, 62.7, 62.1 (2 C, C-6, C-6′), 54.3 (1 C, N(*C*H_2_CH_2_(CH_2_)_11_CH_3_)_2_), 52.8 (1 C, O*C*H_2_CH_2_CH_2_N(CH_2_CH_2_(CH_2_)_11_CH_3_)_2_, 33.0, 30.7, 30.6, 30.5, 30.4, 30.1, 27.6, 25.4, 24.7, 23.7 (25 C, some signals were overlapped, N(CH_2_(*C*H_2_)_12_CH_3_)_2_), OCH_2_*C*H_2_CH_2_N(CH_2_CH_2_(C_11_H_22_)CH_3_)_2_), 14.4, 14.4 (2 C, N(CH_2_(CH_2_)_12_*C*H_3_)_2_).

*3′-(N,N-Ditetradecyl-N-methylaminonium iodine)propyl α-d-glucopyranosyl-(1→4)-β-d-glucopyranoside* (**IX b**). CH_3_I (0.2 g, 1.4 mmol, 88.0 µL) was added in portion to a mixture of compound **VIII b** (280.0 mg, 353.5 µmol) in THF (10.0 mL) at room temperature. The completion of the reaction was detected by TLC (ethyl acetate:methanol = 3:1). The reaction mixture was evaporated to dryness and a solid was precipitated when the acetone was dropped to the syrup in an ice bath. The mixture was filtered, and the filtrate was washed with acetone and dried by vacuum to give **IX b** (0.20 g, 60.6%) as a white powder solid. ^1^H-NMR (MeOD): δ (ppm): 5.16 (d, 1 H, *J*_1′,2′_ = 3.5 Hz, H-1′), 4.32 (d, 1 H, *J*_1,2_ = 8.0 Hz, H-1), 3.97–3.41 (m, 15 H, H-6a′, H-6b′, H-6a, H-6b, H-3′, OC*H*_2_CH_2_CH_2_N(CH_2_CH_2_(CH_2_)_11_CH_3_)_2_, OCH_2_CH_2_C*H*_2_N(CH_2_CH_2_(CH_2_)_11_CH_3_)_2_, H-3, H-5, H-4′, H-4, H-2, H-5′), 3.30–3.20 (m, 5 H, H-2, N(C*H*_2_CH_2_(CH_2_)_11_CH_3_)_2_), 3.03 (s, 1 H, (C*H*_3_)N(CH_2_(CH_2_)_12_CH_3_)_2_), 2.07–1.99 (m, 2 H, OCH_2_C*H*_2_CH_2_N(CH_2_CH_2_(CH_2_)_11_CH_3_)_2_, 1.78–1.67 (m, 4 H, N(CH_2_C*H*_2_(CH_2_)_11_CH_3_)_2_), 1.40–1.25 (m, 44 H, N(CH_2_CH_2_(C*H*_2_)_11_CH_3_)_2_), 0.87 (t, 6 H, *J* = 7.0 Hz, N(CH_2_CH_2_(C_11_H_22_)C*H*_3_)_2_); ^13^C-NMR (MeOD): δ (ppm): 104.2 (1 C, C-1), 102.8 (1 C, C-1′), 81.0, 77.7, 76.5, 74.9, 74.8, 74.6, 74.0, 71.5, 67.3, 62.7, 62.6, 61.9, 60.7 (14 C, C-4′, C-5, C-5′, C-2, C-2′, C-3′, C-3, C-4, OCH_2_CH_2_*C*H_2_N(CH_2_CH_2_(CH_2_)_11_CH_3_)_2_, C-6, C-6′, N(*C*H_2_CH_2_(CH_2_)_11_CH_3_)_2_, O*C*H_2_CH_2_CH_2_N(CH_2_CH_2_(CH_2_)_11_CH_3_)_2_, 49.0 (1 C, (*C*H_3_)N(C_14_H_29_)_2_), 33.0, 30.7, 30.7, 30.6, 30.5, 30.4, 30.2, 27.4, 24.3, 24.1, 23.7, 23.2 (25 C, some signals were overlapped, N(CH_2_(*C*H_2_)_12_CH_3_)_2_), OCH_2_*C*H_2_CH_2_N(CH_2_CH_2_(C_11_H_22_)CH_3_)_2_), 14.5, 14.5 (2 C, N(CH_2_(CH_2_)_12_*C*H_3_)_2_). HRMS: *m*/*z* = 807.6521, in agreement with the calculated mass for [M]^+^ = C_44_H_88_NO_11_^+^.

#### 3.1.3. Synthesis of Cationic Glycolipids **IX c** (Malt-DiC16MA)

*3′-(N,N-Dihexadecylamino)propyl α-d-glucopyranosyl-(1→4)-β-d-glucopyranoside* (**VIII c**). The mixture of compound **VII** (560.0 mg, 1.4 mmol), 1-bromohexadecane (1.7 g, 5.6 mmol, 1.7 mL), anhydrous K_2_CO_3_ (0.20 g), CH_3_OH (10.0 mL), CH_3_CH_2_OH (4.0 mL) was refluxed at 75 °C until TLC (ethyl acetate:methanol = 3:1) showed the reaction was completed. The mixture was diluted with DCM (20.0 mL), washed with water for two times. The organic phase was dried by anhydrous Na_2_SO_4_ and concentrated to dryness. The residue was purified by silica gel column chromatography with ethyl acetate:methanol = 5:1 as the eluent to give compound **VIII c** (0.49 g, 40.8%) as a white powder solid. ^1^H-NMR (MeOD): δ (ppm): 5.19 (d, 1 H, *J*_1′,2′_ = 3.5 Hz, H-1′), 4.39 (d, 1 H, *J*_1,2_ = 8.0 Hz, H-1), 4.07–4.03 (m, 1 H, OCH_2_CH_2_CH*H*N(CH_2_CH_2_(CH_2_)_13_CH_3_)_2_), 3.95–3.92 (dd, 1 H, *J*_6a′,6b′_ = 2.0 Hz, *J*_6a′,5′_ = 4.0 Hz, H-6a′), 3.88–3.35 (m, 12 H, H-6b′, H-6a, H-6b, H-3′, OCH_2_CH_2_CH*H*N(CH_2_CH_2_(CH_2_)_13_CH_3_)_2_, H-3, H-5, H-4′, H-2′, H-5′, OC*H*_2_CH_2_CH_2_N(CH_2_CH_2_(CH_2_)_13_CH_3_)_2_), 3.31–3.22 (m, 2 H, H-4, H-2), 3.22–3.16 (m, 4 H, N(C*H*_2_CH_2_(CH_2_)_13_CH_3_)_2_), 2.13–2.04 (m, 2 H, OCH_2_C*H*_2_CH_2_N(CH_2_ CH_2_(CH_2_)_13_CH_3_)_2_, 1.81–1.72 (m, 4 H, N(CH_2_C*H*_2_(CH_2_)_13_CH_3_)_2_), 1.44–1.31 (m, 52 H, N(CH_2_CH_2_(C*H*_2_)_13_CH_3_)_2_), 0.93 (t, 6 H, *J* = 7.0 Hz, N(CH_2_CH_2_(C_13_H_26_)C*H*_3_)_2_); ^13^C-NMR (MeOD): δ (ppm): 104.2 (1 C, C-1), 102.9 (1 C, C-1′), 81.3, 77.7, 76.7, 75.0, 74.8, 74.6, 74.1, 71.5 (8 C, C-4′, C-5, C-5′, C-2, C-2′, C-3′, C-3, C-4), 68.4 (1 C, OCH_2_CH_2_*C*H_2_N(CH_2_CH_2_(CH_2_)_13_CH_3_)_2_, 62.7, 62.1 (2 C, C-6, C-6′), 54.3 (1 C, N(*C*H_2_CH_2_(CH_2_)_13_CH_3_)_2_), 52.8 (1 C, O*C*H_2_CH_2_CH_2_N(CH_2_CH_2_(CH_2_)_13_CH_3_)_2_, 33.0, 30.8, 30.7, 30.6, 30.5, 30.4, 30.1, 27.6, 25.4, 24.7, 23.7 (29 C, some signals were overlapped, N(CH_2_(*C*H_2_)_14_CH_3_)_2_), OCH_2_*C*H_2_CH_2_N(CH_2_CH_2_(C_13_H_26_)CH_3_)_2_), 14.4, 14.4 (2 C, N(CH_2_(CH_2_)_14_*C*H_3_)_2_).

*3′-(N,N-Dihexadecyl-N-methylaminonium iodine)propyl α-d-glucopyranosyl-(1→4)-β-d-glucopyanoside* (**IX c**). CH_3_I (0.14 g, 0.98 mmol, 60.0 µL) was added in portion to a solution of compound **VIII c** (200.0 mg, 235.8 µmol) in THF (5.0 mL) at room temperature. The completion of the reaction was detected by TLC (ethyl acetate:methanol = 3:1). The reaction mixture was evaporated to dryness and a white solid was precipitated when the acetone was dropped to the syrup in an ice bath. The mixture was filtered, and the filtrate was washed with acetone and dried by vacuum to give **IX c** (0.18 g, 78.3%) as a white powder. ^1^H-NMR (MeOD): δ (ppm): 5.11 (d, 1 H, *J*_1′,2′_ = 3.5 Hz, H-1′), 4.26 (d, 1 H, *J*_1,2_ = 8.0 Hz, H-1), 3.93–3.33 (m, 15 H, H-6a′, H-6b′, H-6a, H-6b, H-3′, OC*H*_2_CH_2_CH_2_N (CH_2_CH_2_(CH_2_)_13_CH_3_)_2_, OCH_2_CH_2_C*H*_2_N(CH_2_CH_2_(CH_2_)_13_CH_3_)_2_, H-3, H-5, H-4′, H-4, H-2, H-5′), 3.30–3.20 (m, 5 H, H-2, N(C*H*_2_CH_2_(CH_2_)_13_CH_3_)_2_), 2.99 (s, 1 H, (C*H*_3_)N(CH_2_(CH_2_)_14_CH_3_)_2_), 2.05–1.95 (m, 2 H, OCH_2_C*H*_2_CH_2_N(CH_2_CH_2_(CH_2_)_13_CH_3_)_2_, 1.73–1.64 (m, 4 H, N(CH_2_C*H*_2_(CH_2_)_13_CH_3_)_2_), 1.40–1.25 (m, 52 H, N(CH_2_CH_2_(C*H*_2_)_13_CH_3_)_2_), 0.84 (t, 6 H, *J* = 7.0 Hz, N(CH_2_CH_2_(C_13_H_26_)C*H*_3_)_2_); ^13^C-NMR (MeOD): δ (ppm): 104.4 (1 C, C-1), 103.2 (1 C, C-1′), 81.4, 77.9, 76.7, 75.2, 74.9, 74.8, 74.3, 71.6, 67.3, 62.7, 62.1, 60.9 (14 C, C-4′, C-5, C-5′, C-2, C-2′, C-3′, C-3, C-4, OCH_2_CH_2_*C*H_2_N(CH_2_CH_2_(CH_2_)_13_CH_3_)_2_, C-6, C-6′, N(*C*H_2_CH_2_(CH_2_)_13_CH_3_)_2_, O*C*H_2_CH_2_CH_2_N(CH_2_CH_2_(CH_2_)_13_CH_3_)_2_, 49.0 (1 C, (*C*H_3_)N(C_16_H_33_)_2_), 33.1, 30.8, 30.7, 30.6, 30.5, 30.4, 30.2, 27.4, 24.3, 24.1, 23.7, 23.1 (29 C, some signals were overlapped, N(CH_2_(*C*H_2_)_14_CH_3_)_2_), OCH_2_*C*H_2_CH_2_N(CH_2_CH_2_(C_13_H_26_)CH_3_)_2_), 14.4, 14.4 (2 C, N(CH_2_(CH_2_)_14_*C*H_3_)_2_). HRMS: *m*/*z* = 963.7687, in agreement with the calculated mass for [M]^+^ = C_48_H_96_NO_11_^+^.

### 3.2. Preparation of Liposome and Liposome/pDNA Complexes (Lipoplexes)

Lipids (2.5 × 10^−3^ mmol,Malt-DiC12MA, Malt-DiC14MA and Malt-DiC16MA) were weighted, then dissolved in 10 mL of sterile double distilled water and sonicated to clarity at 37 °C for 30 min in a closed vial, which was subsequently filtered with syringe filter (0.45 μm) to give the solution of cationic liposomes (0.25 mmol/L). Furthermore, the pDNA (0.8 μg, OD_260_/OD_280_ > 1.8, the optical density commonly abbreviated to OD) was diluted in 50 μL of Opti-MEM. Liposome/pDNA complexes were formulated by adding diluted liposome solution into pDNA solution at different N/P ratios (2:1, 4:1, 6:1, 8:1 and 10:1) and incubated for 30 min at room temperature.

### 3.3. Cells Culture

Hek293 Cells were cultured in cell culture medium DMEM supplemented with 10% (*v*:*v*) FBS, 100 units/mL penicillin and 100 μg/mL streptomycin in T55 culture dish. The cell line was cultured under a 95% relative humidified atmosphere with a CO_2_ level of 5% at 37 °C.

### 3.4. Gel Retardation Assay

The interaction of cationic glycolipids and pDNA were determined by gel retardation assay. Five ratios of liposomes/pDNA complex (lipoplexes, 0.3:1, 0.5:1, 1:1, 2:1, 3:1) were prepared as described in Section 3.2. Naked pDNA was used as a control. After 30 min of incubation, the lipoplexes were electrophoresed (DYY-6C, Beijing Liuyi Biotechnology Co. Ltd., Beijing, China) on the 1% agarose gel containing ethidium bromide (EB) in TBE buffer (89 mM Tris, 89 mM boric acid, 2 mM EDTA (Ethylenediaminetetraacetic acid), pH = 8.3) at 110 V for 30 min, and then DNA was visualized under an ultraviolet (UV) illuminator (Tanon 2500, Shanghai Tianneng Science and Technology Co. Ltd., Shanghai, China).

### 3.5. Dynamic Light Scattering

Average size, PDI distribution and zeta potential of cationic liposomes or lipoplexes at various N/P were measured by dynamic light scattering system (Zetasizer Nano ZS90, Malvern Instruments Ltd., Worcestershire, UK) at 25 °C. Before measurement, the liposomes and lipoplexes were diluted to 1 mL with sterile double distilled water. Furthermore, the size, PDI and zeta potential were reported as the mean ± standard deviation (SD) (*n* = 3).

### 3.6. Morphology Study by Atomic Force Microscopy

The morphology of naked cationic liposomes and lipoplexes were assessed by atom force microscopy. The lipoplexes sample were prepared by mixing cationic liposomes with pDNA (0.8 μg) at N/P ratio of 8:1 in deionized water (100 μL), which performed best in the transfection experiments. Prior to AFM measurements, the different samples were dropped onto a freshly cleaved mica slice and air-dried at room temperature for approximately 20 min. Then, the morphology of liposomes and lipoplexes were performed on Nanoscope IV atomic force microscope (Veeco Instruments, Plainview, NY, USA) at room temperature.

### 3.7. Transfection Biology (GFP Expression Analysis)

To investigate the gene transfection efficiencies of the synthesized glycolipids, the HEK293 cells were seeded at an initial density of 1.2 × 10^5^ cells per well in a 24-well plate with 250 μL of DMEM containing 10% FBS. Prior to transfection, the cells were adhered at the bottom of the plate overnight to reach an 80%–90% density. Then, the regular medium was exchanged with serum-free medium after washed twice with PBS (phosphate buffer saline) and the lipoplexes (as described in Section 3.2) was added to each well with different N/P ratios. After 4 h incubation at 37 °C, the serum-free medium was changed to 10% FBS regular medium. Following the operating process, the commercially available gene transfection reagent, Lipofectamine2000, was used in combination with pDNA as the positive control. The green fluorescent protein was visualized and recorded under an inverted fluorescence microscope (Olympus LX71, Tokyo, Japan) after 24 h incubation.

### 3.8. Cellular Uptake

The HEK293 cells were seeded in a 35-mm laser confocal culture dish at a density of 3 × 10^5^ cells in 2.5 mL DMEM containing 10% FBS and incubated at 37 °C with 5% CO_2_ for 24 h before transfection. After the culture medium was exchanged with FBS-fresh basic medium, the complexes solutions (prepared by mixing Malt-DiC12MA liposomes, Malt-DiC14MA liposomes and Cy3-labeled pEGFP DNA at N/P ratio of 8 and incubated at room temperature for 30 min) were added to the dish and incubated for another 4 h. The culture medium was removed and the cells were washed with PBS buffer solution (0.5×) for three times. Then, Dio and Hochest 33342 were used to stain lysosomes (20 min) and nuclei (15 min), respectively. After washed the cells with PBS buffer solution for three times, fluorescence microscopic images were observed and recorded by a Nikon Ti-U (Tokyo, Japan) invert fluorescence microscope.

### 3.9. Cytotoxicity Assay

Toxicity of each liposome/pDNA complexes toward HEK293 cells were analyzed by a MTT assay following literature procedures [50,51]. The cells were seeded in a 96-well plate at an initial density of 4 × 10^4^ cells per well cultured in 150 μL 10% FBS regular medium DMEM. After 24 h incubation, the lipoplexes were added to each well at different N/P ratios (2:1, 4:1, 6:1, 8:1 and 10:1) and incubated for another 4 h. Then, 20 μL of MTT (5 mg/mL solution in PBS) were added and incubated for an additional 4 h. Media was removed and 200 µL of DMSO was added per well to dissolve the purple formazan crystals. The absorbance at λ = 490 nm was measured by microplate reader (SpectraMax i3x, Sunnyvale, CA, USA). The control cells were not treated with lipoplexes. The percentage of viable cells was then calculated as [A490 (treated cells) − background]/[A490 (untreated cells) − background] × 100.

## 4. Conclusions

In this work, maltose-based cationic lipids with different hydrophobic chain lengths were successfully synthesized and their structure–activity relationship was investigated for gene delivery activities. The results of ^1^H-NMR, ^13^C-NMR, ^1^H-^1^H COSY and ^1^H-^13^C HSQC were used to confirm their structures. The average size, zeta potential, morphology and pDNA binding ability of the glycolipids and lipoplexes were also detected. Importantly, the in vitro delivery results of pDNA by lipoplexes against HEK293 cells indicated that the maltose-based cationic lipid with *n*-C_14_H_29_ hydrocarbon chain shows maximum transfection, better uptake capability and lower cytotoxicity. Therefore, the cationic lipid Malt-DiC14MA has a potential application in the field of designing non-viral vectors for gene therapy.

## Figures and Tables

**Table 1:** The average sizes and zeta potential of lipids **IX a**–**IX c**.

Cationic Liposomes	Average Size (nm)	PDI ^a^	Zeta Potential (mV)
Malt-DiC12MA **IX a**	90.3 ± 3.5	0.221 ± 0.031	+46.0 ± 5.6
Malt-DiC14MA **IX b**	63.4 ± 0.7	0.167 ± 0.025	+36.2 ± 6.8
Malt-DiC16MA **IX c**	109.7 ± 2.6	0.198 ± 0.089	+37.4 ± 4.3

^a^ PDI: Polydispersity Index.

**Table 2:** Average size, PDI distribution and zeta potential of cationic complexes under the different N/P ratio ^a^.

Lipid/DNA Complex	N/P Ratio	Average Size (nm)	PDI	Zeta Potential (mV)
Malt-DiC14MA/DNA	4:1	88.6 ± 6.8	0.125 ± 0.056	+15.1 ± 1.6
Malt-DiC14MA/DNA	6:1	98.6 ± 7.5	0.365 ± 0.036	+14.6 ± 3.5
Malt-DiC14MA/DNA	8:1	78.6 ± 11.5	0.154 ± 0.012	+16.0 ± 7.8
Malt-DiC14MA/DNA	10:1	90.3 ± 4.5	0.365 ± 0.046	+20.4 ± 3.7
Malt-DiC16MA/DNA	4:1	120.6 ± 9.9	0.157 ± 0.036	+19.3 ± 8.6
Malt-DiC16MA/DNA	6:1	127.6 ± 11.2	0.258 ± 0.014	+21.8 ± 6.5
Malt-DiC16MA/DNA	8:1	146.8 ± 5.6	0.147 ± 0.045	+22.6 ± 1.2
Malt-DiC16MA/DNA	10:1	110.6 ± 11.5	0.375 ± 0.046	+26.8 ± 4.6

^a^ According to the results of pEGFP-C1 (Enhanced Green Fluorescent Protein plasmid) transfection, Malt-DiC14MA and Malt-DiC16MA liposomes possess transfection capacity at N/P ratio of 4:1, 6:1, 8:1 and 10:1. Therefore, these N/P ratios were selected to investigate the particle size and zeta potential by DLS (dynamic light scattering).

## Supplementary Materials

Supplementary materials are available online.
