# Supplementary material for: Evaluation of Maltose-Based Cationic Liposomes with Different Hydrophobic Tails for Plasmid DNA Delivery
Source: Molecules. 2017 Mar 12;22(3):406. doi: 10.3390/molecules22030406 (PMC6155304; doi:10.3390/molecules22030406)
Supplement: Supplementary file 1 [file molecules-22-00406-s001.pdf]

Evaluation of maltose-based cationic liposomes with different hydrophobic tails for plasmid DNA delivery

Bo Li[†] , Liangliang Deng[†] , Meiyan Liu, Youlin Zeng[*]

(*National & Local Joint Engineering Laboratory for New Petro-chemical Materials and Fine Utilization of Resources, Hunan Normal University, Changsha, Hunan, 410081, P. R. China*)

Contents

Fig. S1 ··S1
NMR spectrums of intermediates and lipids **IX a** (**Malt-DiC12MA**)··············S2
NMR spectrums of intermediates and lipids **IX b** (**Malt-DiC14MA**)··············S22
NMR spectrums of intermediates and lipids **IX c** (**Malt-DiC16MA**)··············S30

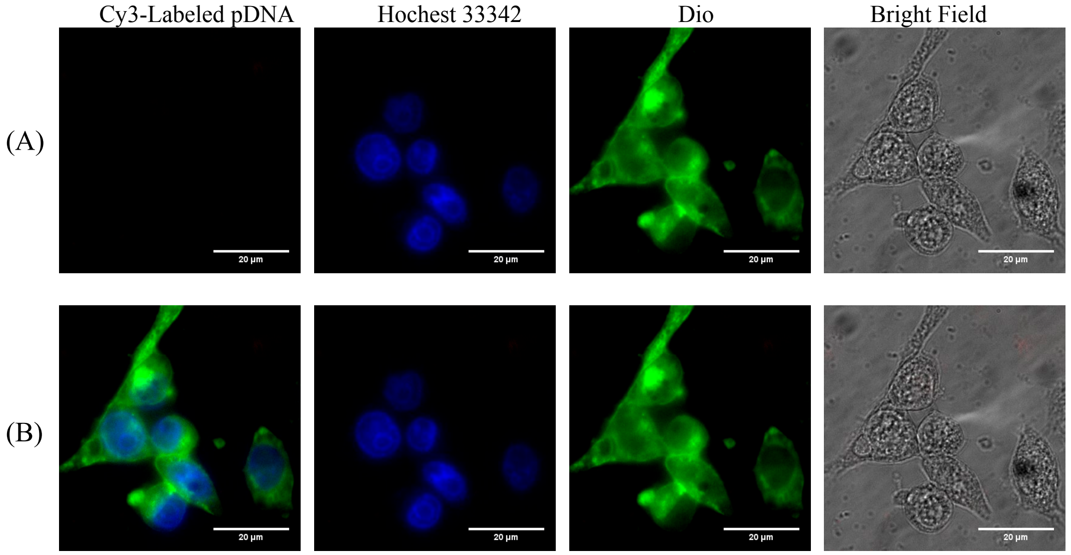

Fig. S1 Fluorescence microscopic images (100×) of cellular uptake of Malt-DiC12MA Lipids/DNA complexes at the N/P ratio of 8:1 in HEK293 after 4 h of gene transfection. The upper column (A) represents single fluorescent images, the down column (B) represents merger images. Bar: 20 μm. (Green: Dio label cytomembrane, Red: Cy3-labeled pDNA, Blue: Hochest 33342 stained cell nuclei).

[*] Corresponding author. Tel.:+86 13975805056; fax: +86 73188872531
E-mail address: youlinzengcn@gmail.com (Youlin Zeng).
Project supported by the Nation Nature Sciences Foundation of China General Program: 21272064)
[†]These authors have contributed equally to this work.

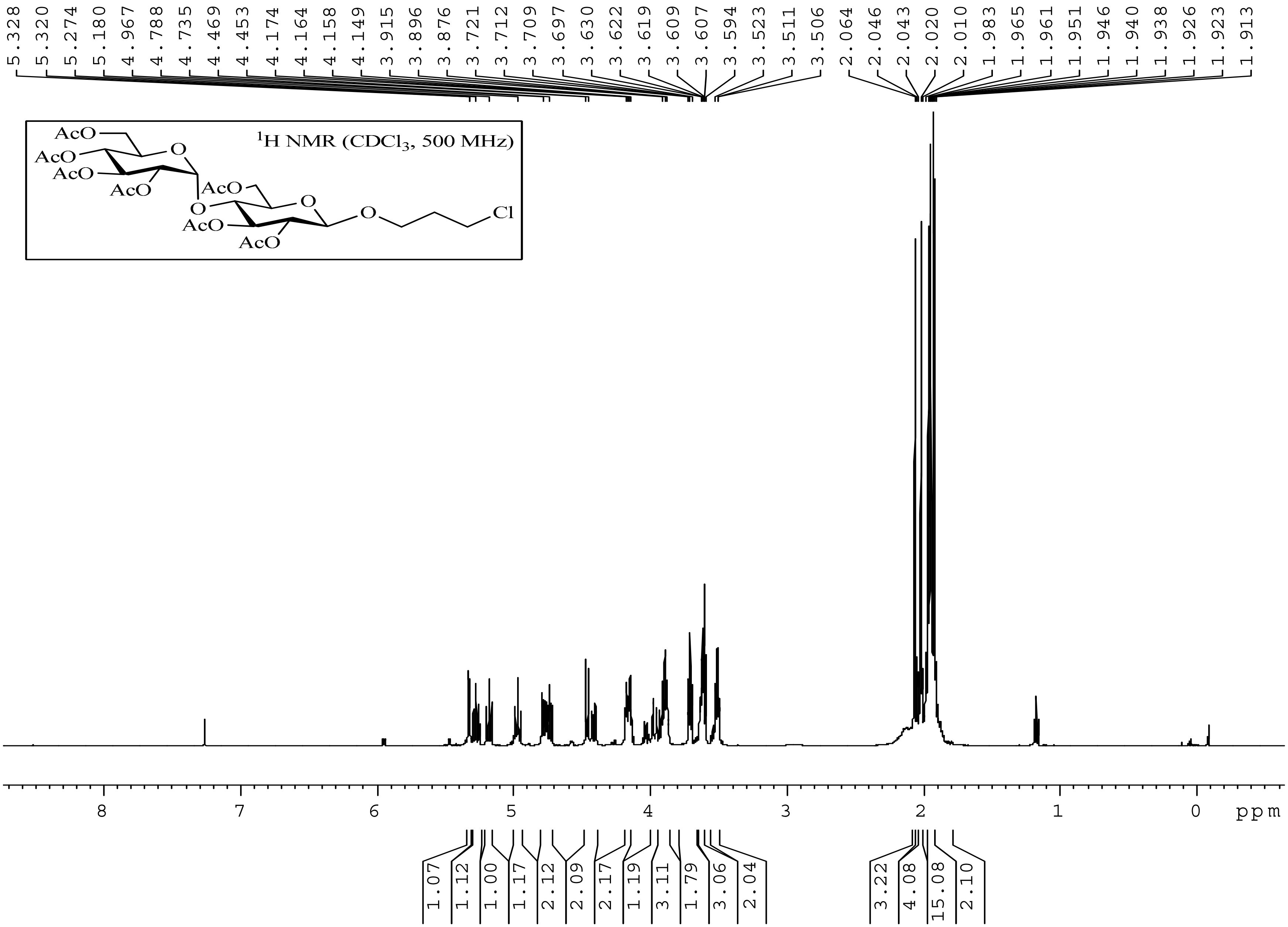
5.328 5.320 5.274 5.180 4.967 4.788 4.735 4.469 4.453 4.174 4.164 4.158 4.149 3.915 3.896 3.876 3.721 3.712 3.709 3.697 3.630 3.622 3.619 3.609 3.607 3.594 3.523 3.511 3.506
2.064 2.046 2.043 2.020 2.010 1.983 1.965 1.961 1.951 1.946 1.940 1.938 1.926 1.923 1.913
AcO
AcO
AcO
AcO
O
AcO
O
O
AcO
AcO
O
Cl
¹H NMR (CDCl₃, 500 MHz)
8 7 6 5 4 3 2 1 0 ppm
1.07 1.12 1.00 1.17 2.12 2.09 2.17 1.19 3.11 1.79 3.06 2.04
3.22 4.08 15.08 2.10

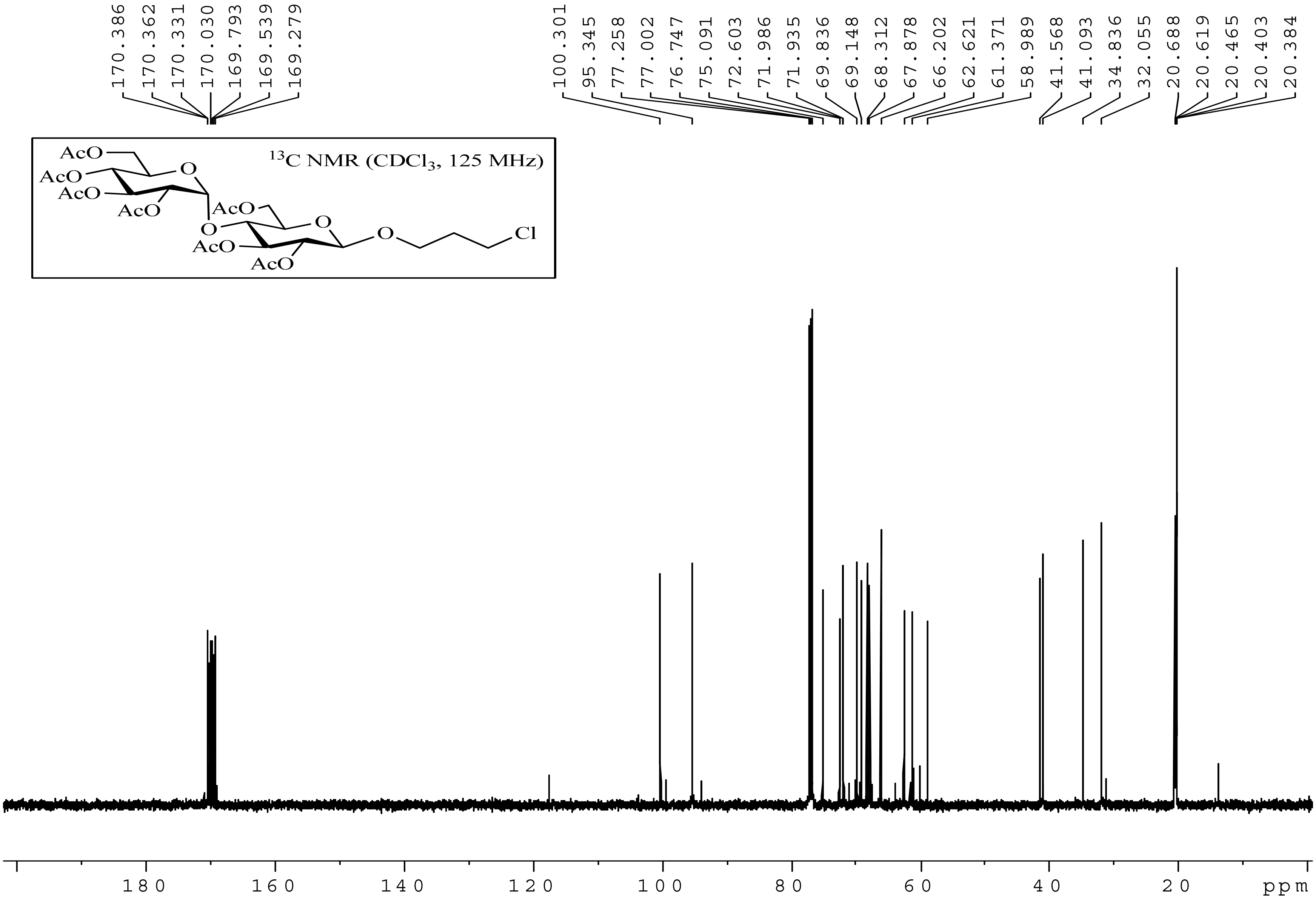
170.386
170.362
170.331
170.030
169.793
169.539
169.279
100.301
95.345
77.258
77.002
76.747
75.091
72.603
71.986
71.935
69.836
69.148
68.312
67.878
66.202
62.621
61.371
58.989
41.568
41.093
34.836
32.055
20.688
20.619
20.465
20.403
20.384
AcO
AcO
AcO
AcO
O
AcO
O
O
AcO
AcO
O
Cl
13C NMR (CDCl3, 125 MHz)
180
160
140
120
100
80
60
40
20
ppm

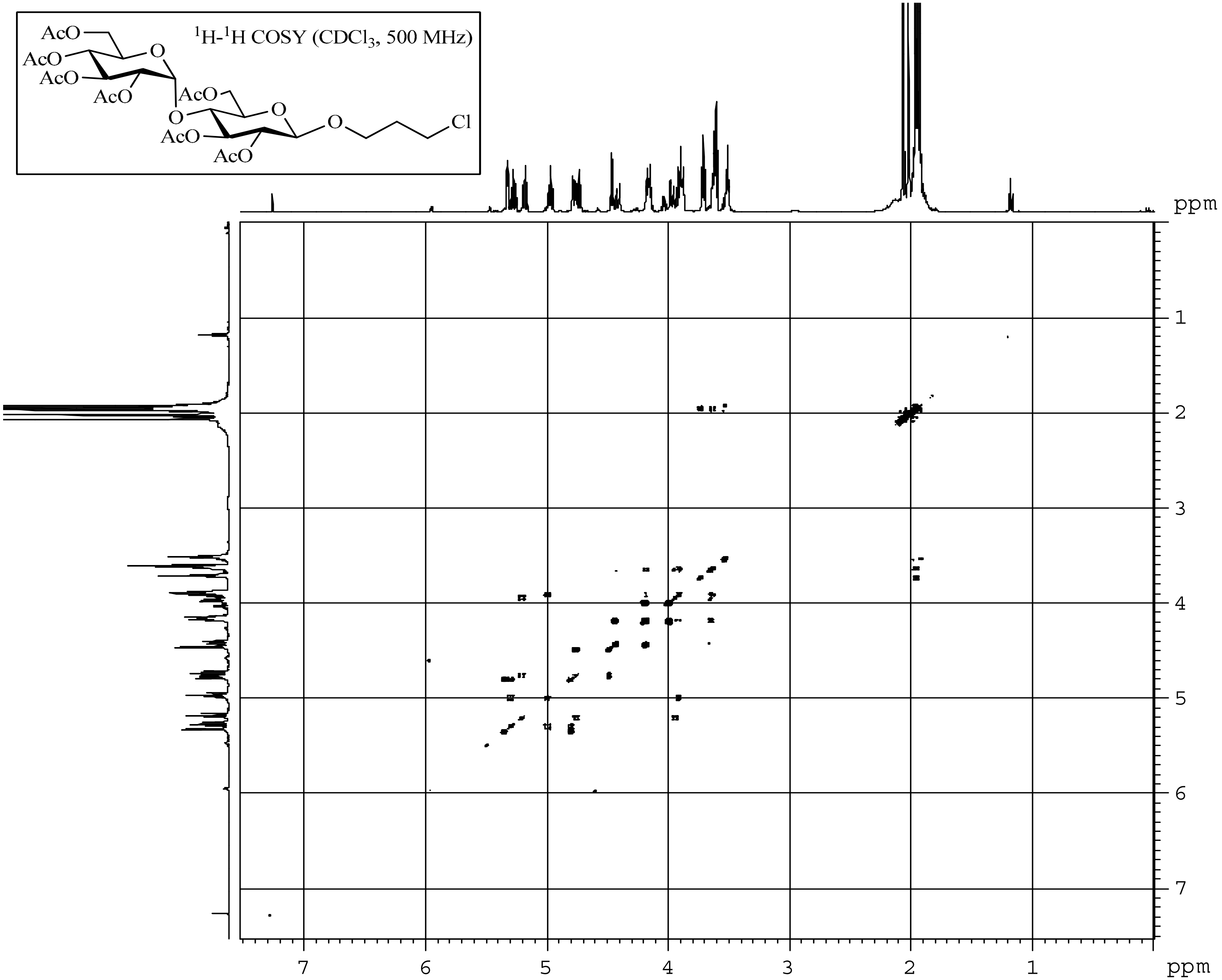
AcO
AcO
AcO
AcO
O
AcO
O
O
AcO
AcO
O
Cl
1H-1H COSY (CDCl3, 500 MHz)
ppm
1
2
3
4
5
6
7
7
6
5
4
3
2
1
ppm

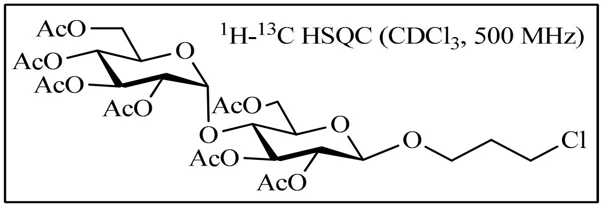
AcO
AcO
AcO
AcO
O
AcO
O
AcO
AcO
O
O
Cl
^{1}H-^{13}C HSQC (CDCl$_3$, 500 MHz)

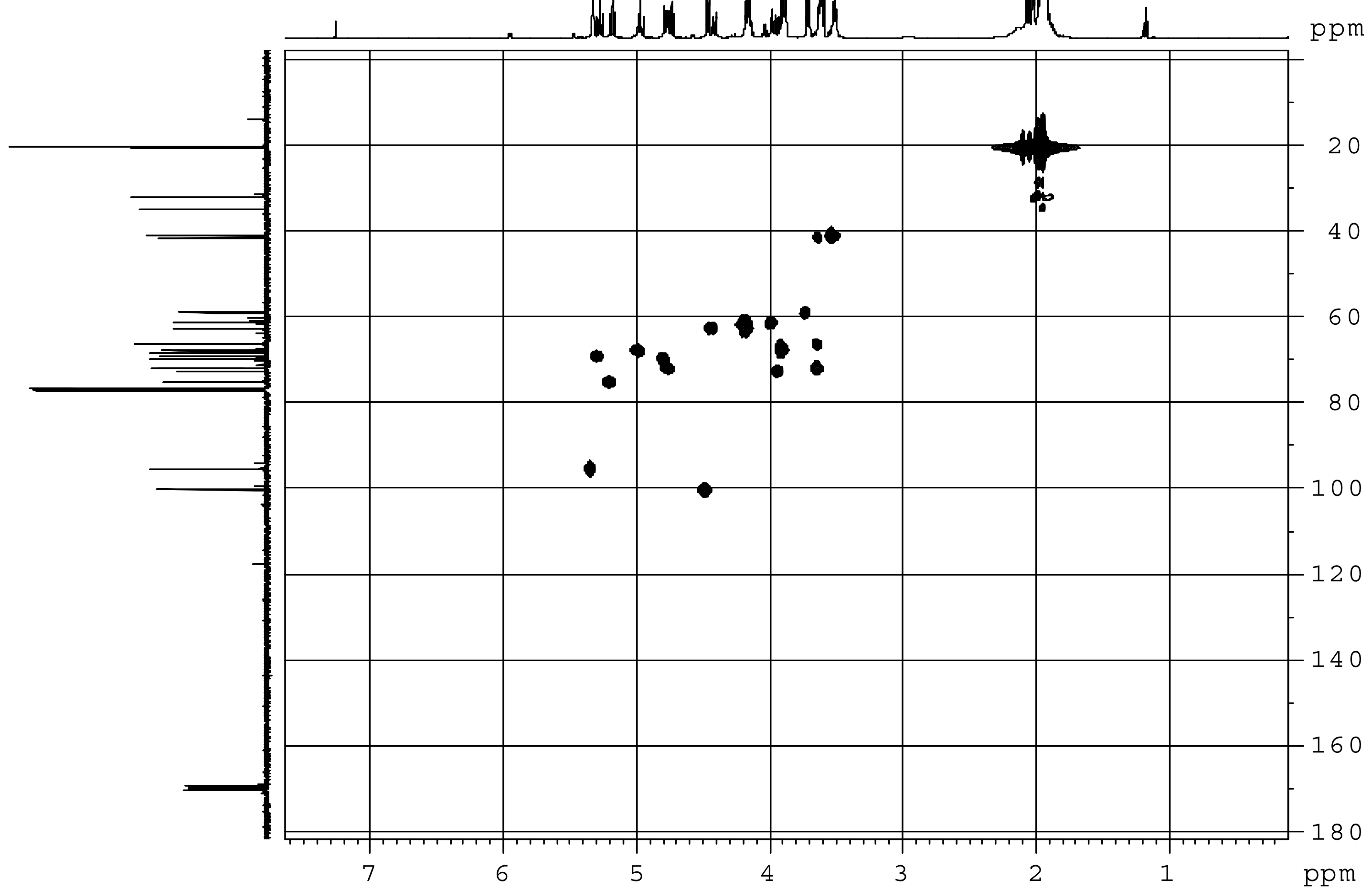
ppm
7
6
5
4
3
2
1
ppm
20
40
60
80
100
120
140
160
180

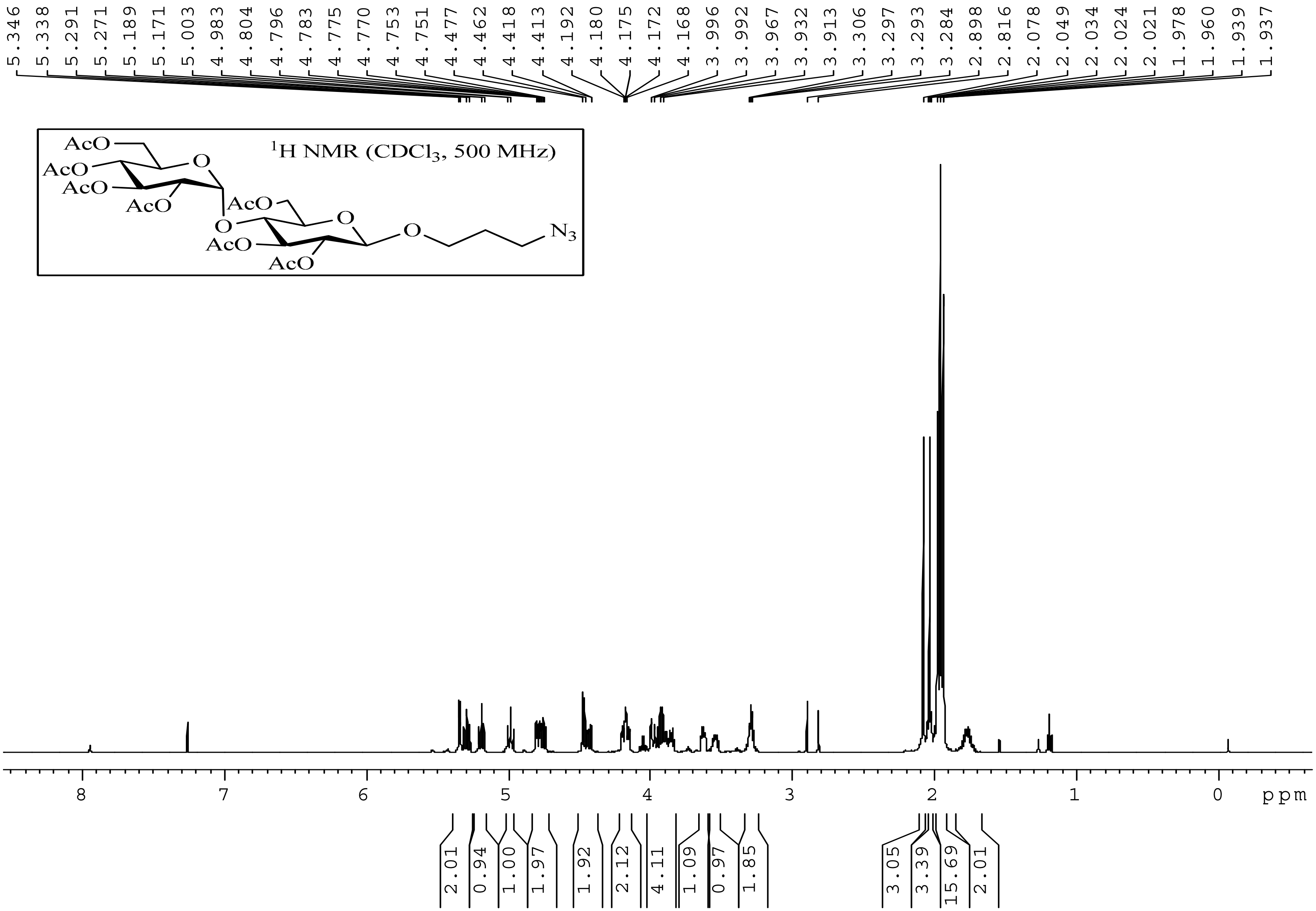

5.346 5.338 5.291 5.271 5.189 5.171 5.003 4.983 4.804 4.796 4.783 4.775 4.770 4.753 4.751 4.477 4.462 4.418 4.413 4.192 4.180 4.175 4.172 4.168 3.996 3.992 3.967 3.932 3.913 3.306 3.297 3.293 3.284 2.898 2.816 2.078 2.049 2.034 2.024 2.021 1.978 1.960 1.939 1.937
^{1}H NMR (CDCl$_3$, 500 MHz)
AcO
AcO
AcO
AcO
AcO
O
O
AcO
AcO
O
O
N$_3$
8 7 6 5 4 3 2 1 0 ppm
2.01 0.94 1.00 1.97 1.92 2.12 4.11 1.09 0.97 1.85 3.05 3.39 15.69 2.01

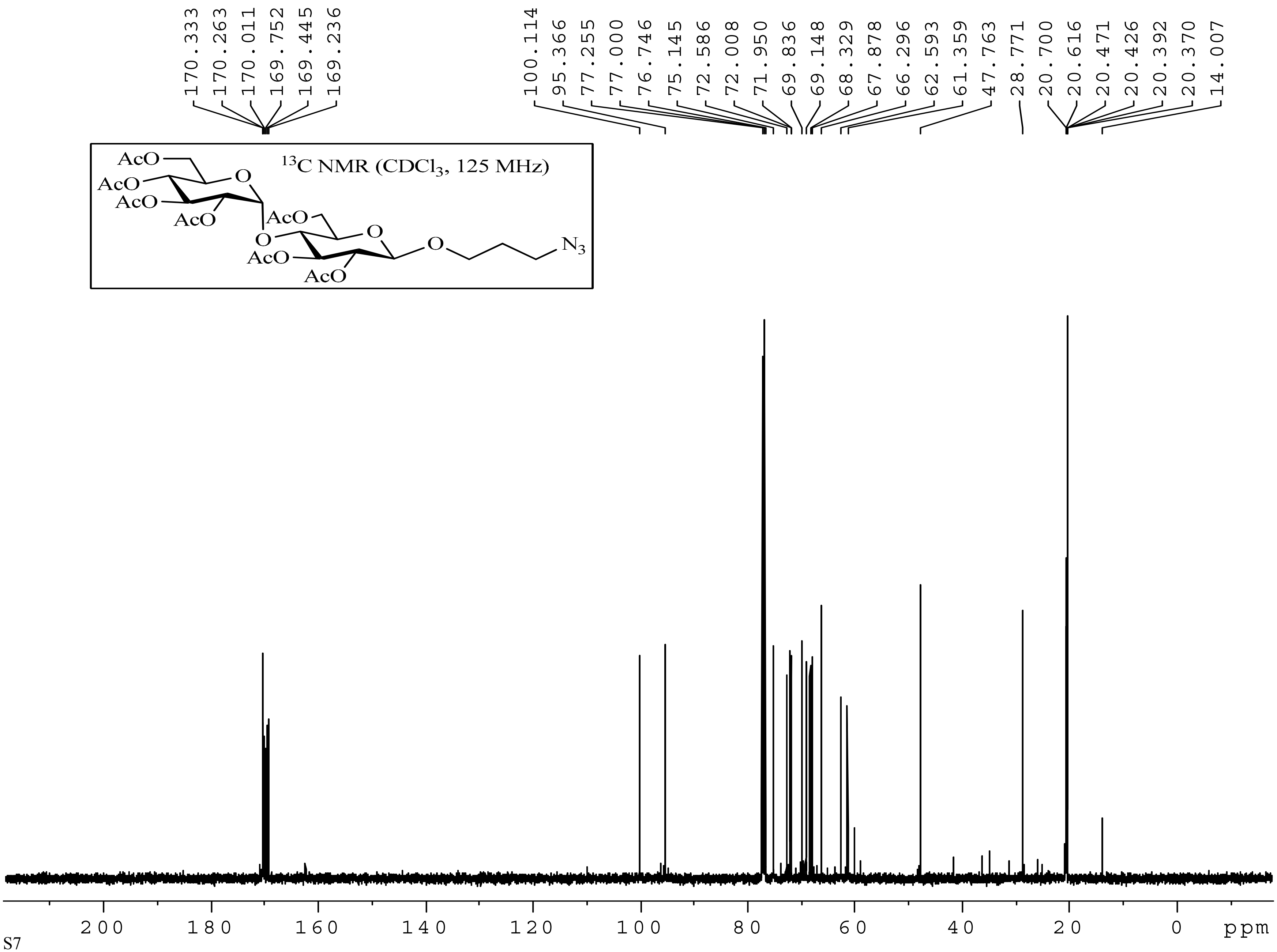
170.333
170.263
170.011
169.752
169.445
169.236
100.114
95.366
77.255
77.000
76.746
75.145
72.586
72.008
71.950
69.836
69.148
68.329
67.878
66.296
62.593
61.359
47.763
28.771
20.700
20.616
20.471
20.426
20.392
20.370
14.007
13C NMR (CDCl3, 125 MHz)
AcO
AcO
AcO
AcO
AcO
O
O
AcO
AcO
O
O
N3
200
180
160
140
120
100
80
60
40
20
0
ppm

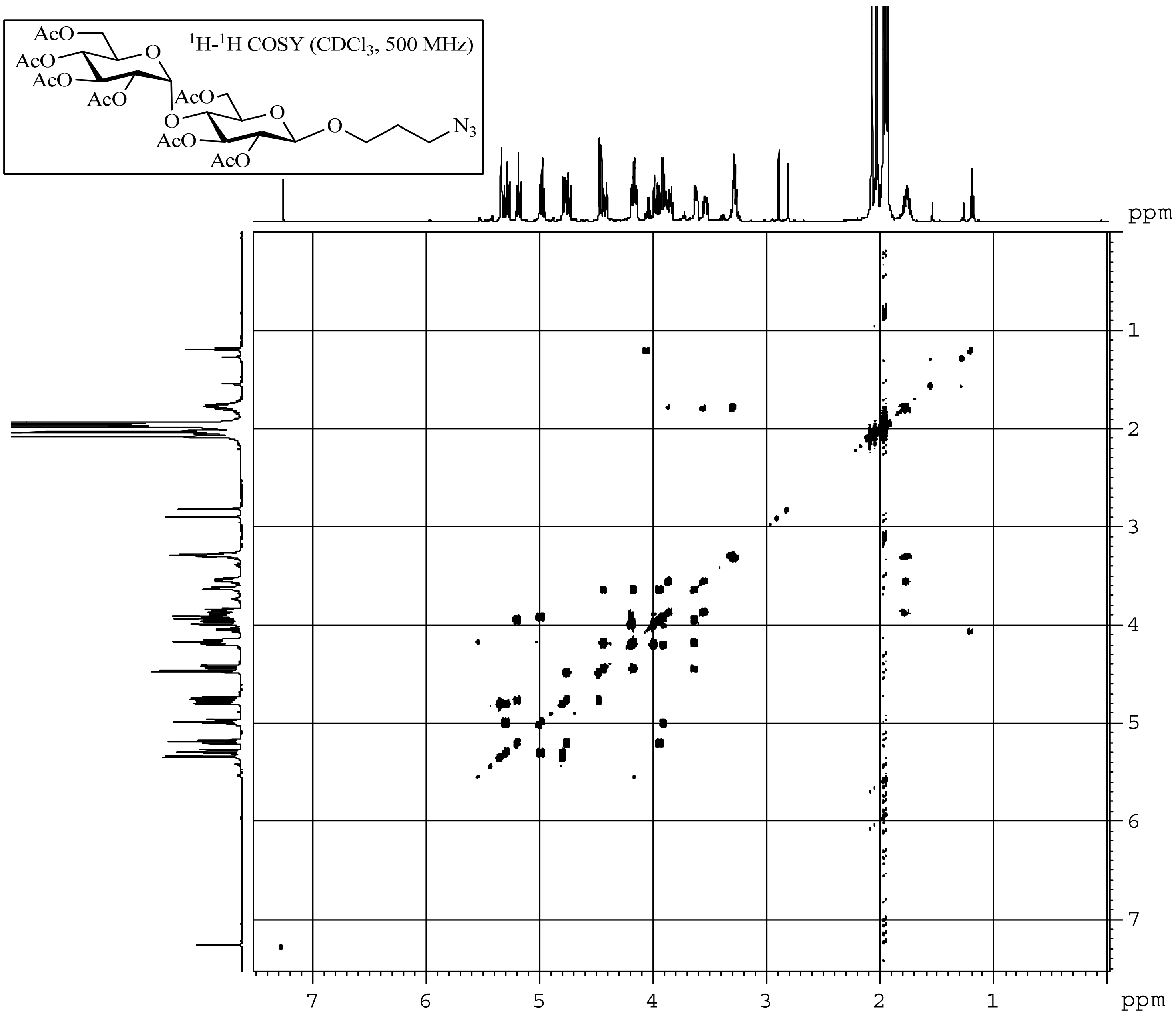
AcO
AcO
AcO
AcO
O
AcO
O
AcO
O
AcO
AcO
O
N3
1H-1H COSY (CDCl3, 500 MHz)
ppm
1
2
3
4
5
6
7
7
6
5
4
3
2
1
ppm

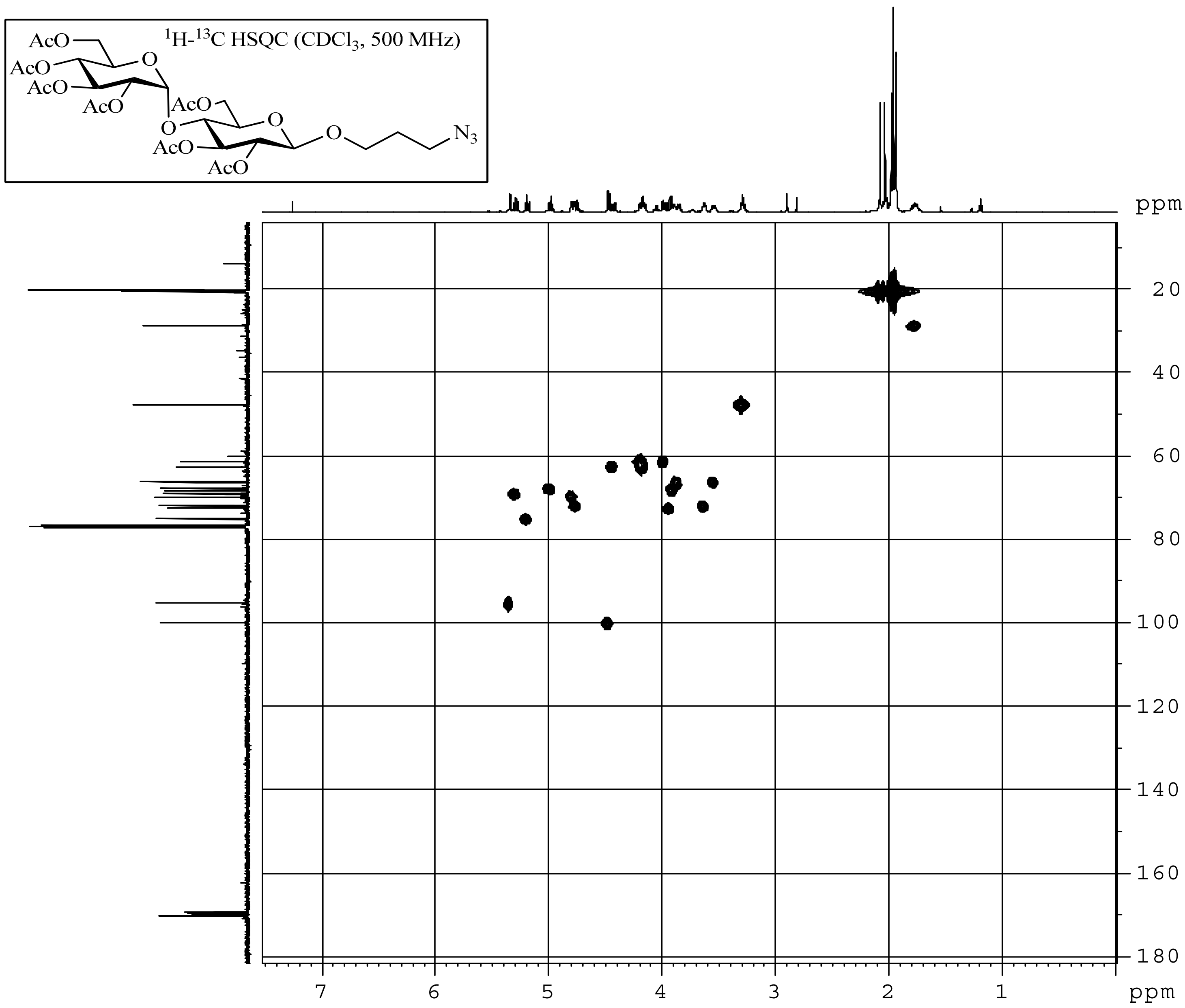

AcO
AcO
AcO
AcO
O
AcO
O
O
AcO
AcO
O
N3
1H-13C HSQC (CDCl3, 500 MHz)
ppm
20
40
60
80
100
120
140
160
180
7
6
5
4
3
2
1
ppm

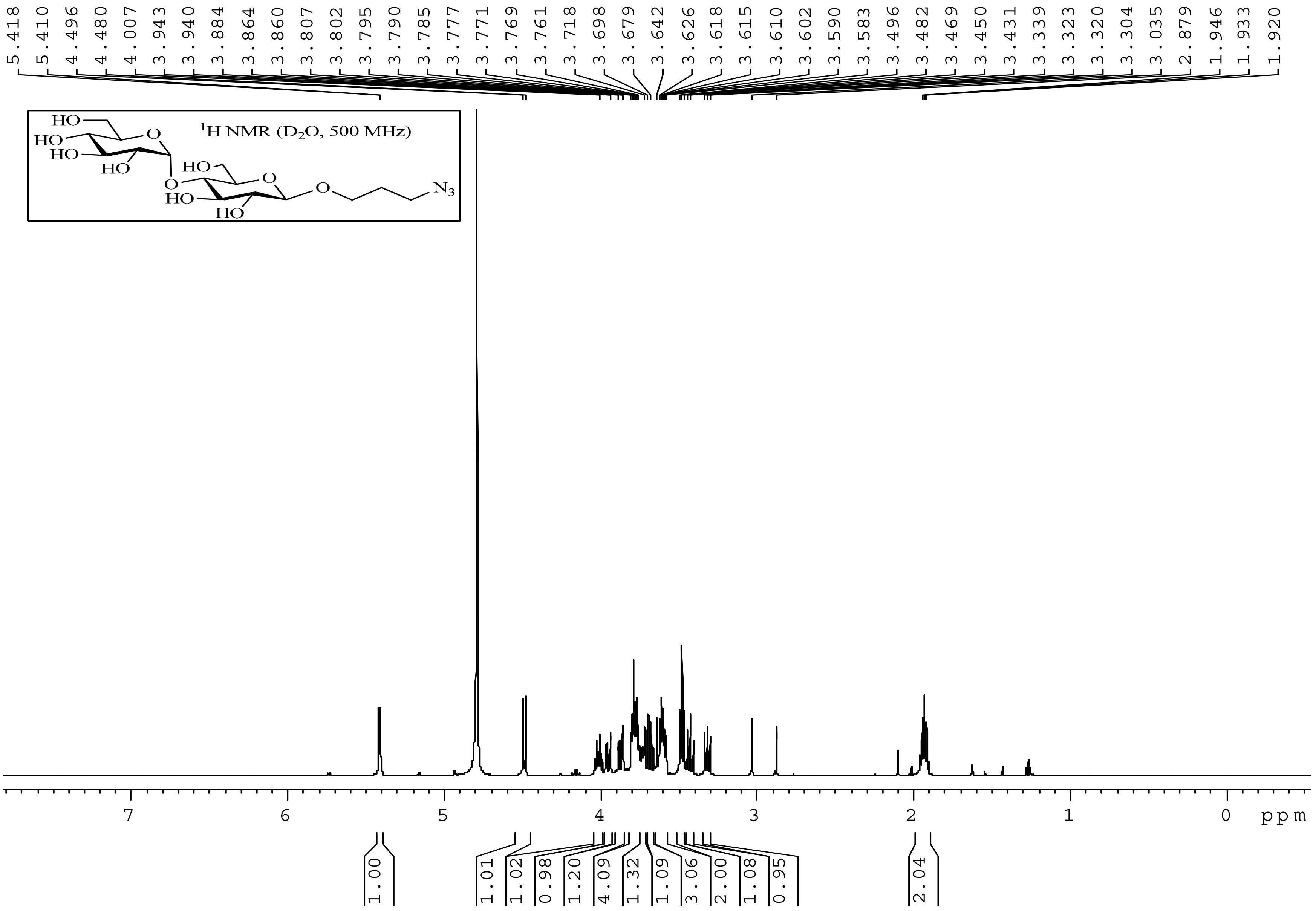

^{1}H NMR (D_2O, 500 MHz)
HO
HO
HO
HO
O
O
HO
O
HO
HO
O
N_3
5.418
5.410
4.496
4.480
4.007
3.943
3.940
3.884
3.864
3.860
3.807
3.802
3.795
3.790
3.785
3.777
3.771
3.769
3.761
3.718
3.698
3.679
3.642
3.626
3.618
3.615
3.610
3.602
3.590
3.583
3.496
3.482
3.469
3.450
3.431
3.339
3.323
3.320
3.304
3.035
2.879
1.946
1.933
1.920
7
6
5
4
3
2
1
0
ppm
1.00
1.01
1.02
0.98
1.20
4.09
1.32
1.09
3.06
2.00
1.08
0.95
2.04

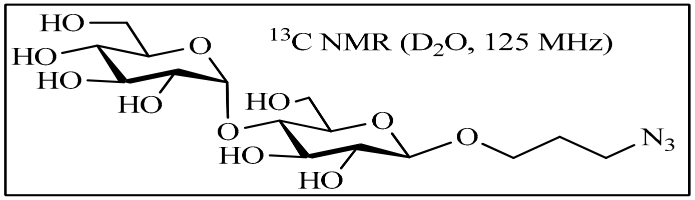
HO
HO
HO
HO
O
O
HO
O
HO
HO
O
N3
13C NMR (D2O, 125 MHz)

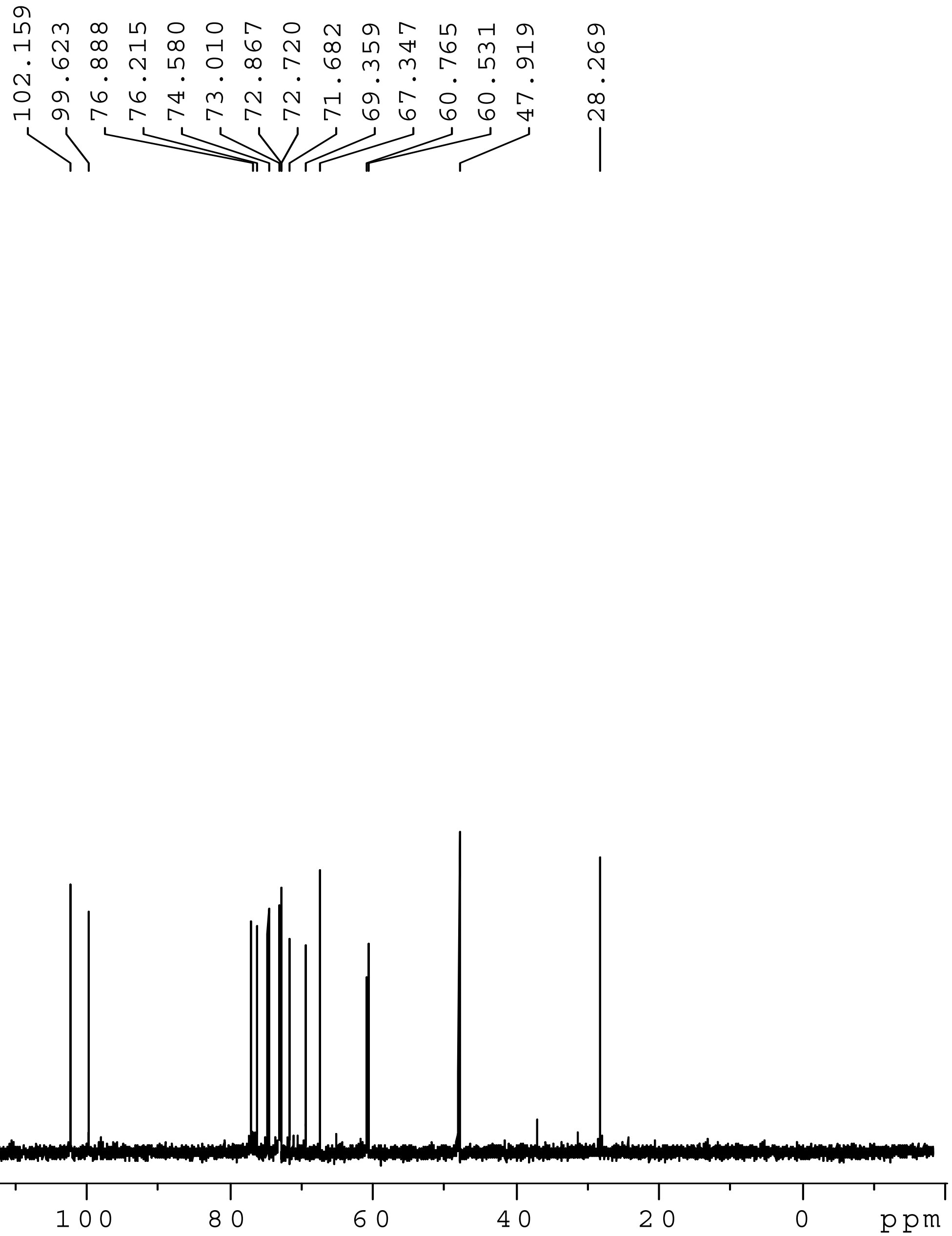
102.159
99.623
76.888
76.215
74.580
73.010
72.867
72.720
71.682
69.359
67.347
60.765
60.531
47.919
28.269
200
180
160
140
120
100
80
60
40
20
0
ppm

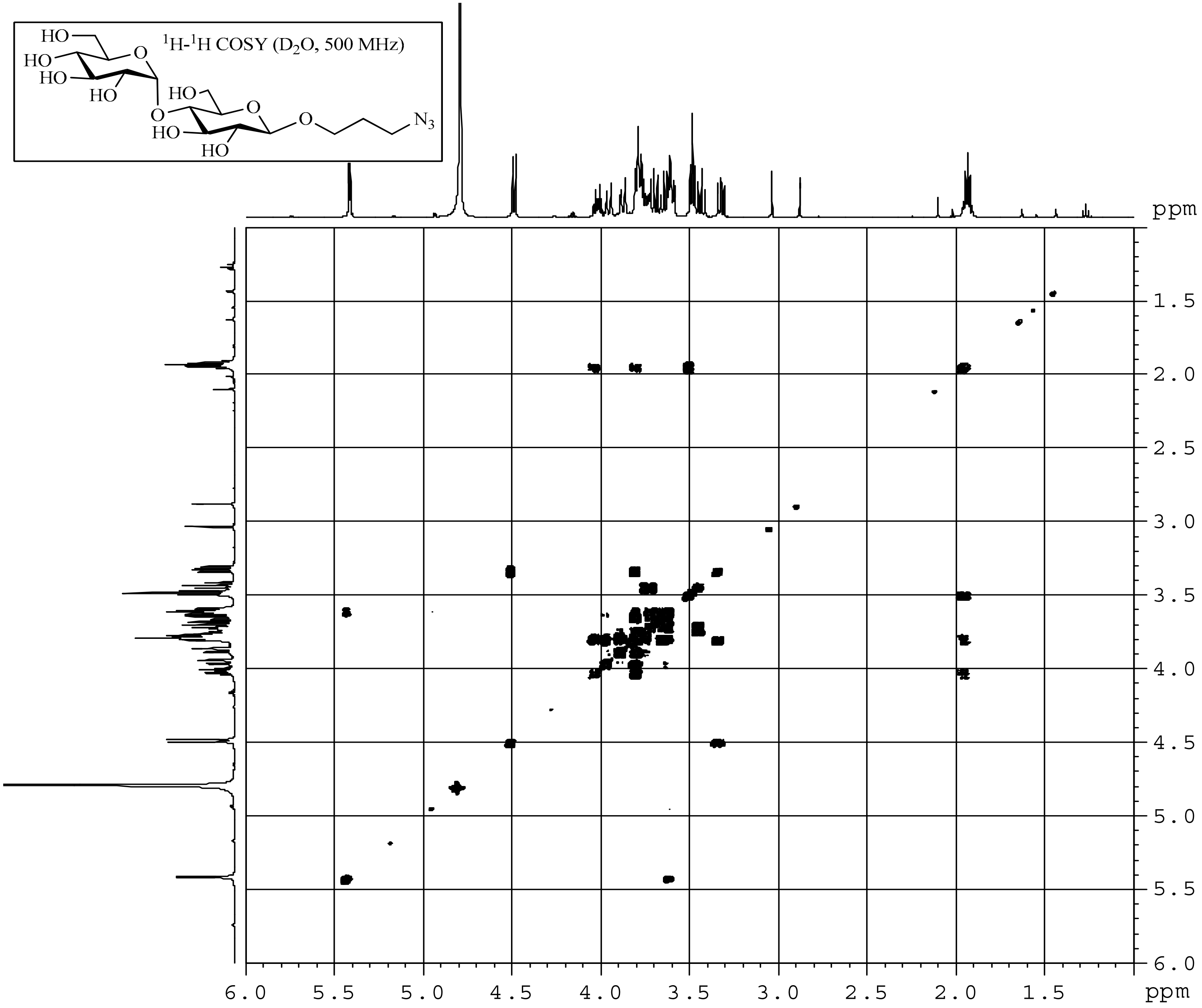

^1H-^1H COSY (D_2O, 500 MHz)
HO
O
HO
HO
HO
O
HO
O
O
HO
HO
N_3
ppm
1.5
2.0
2.5
3.0
3.5
4.0
4.5
5.0
5.5
6.0
6.0
5.5
5.0
4.5
4.0
3.5
3.0
2.5
2.0
1.5
ppm

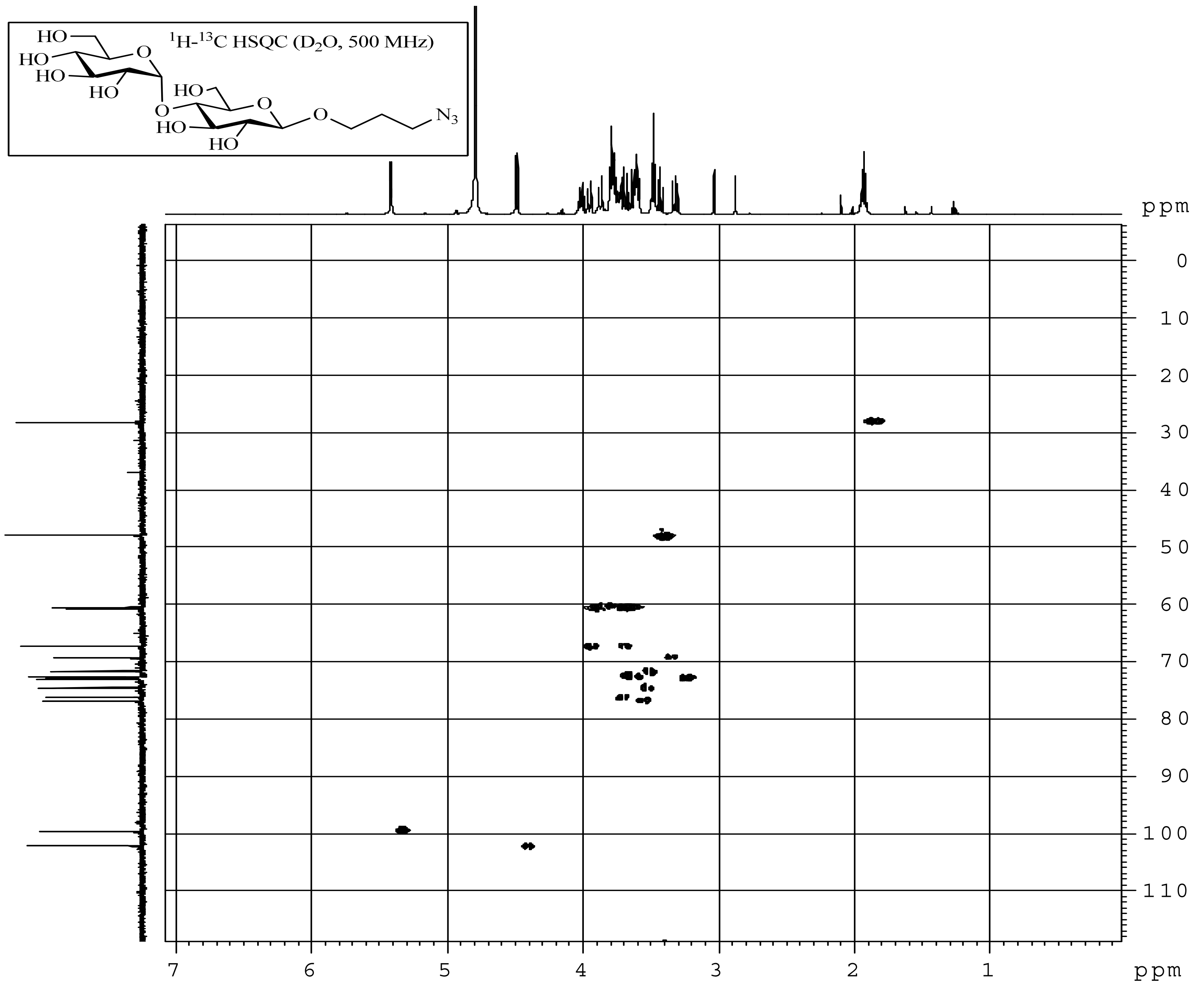

^1H-^{13}C HSQC (D_2O, 500 MHz)
HO
HO
HO
HO
O
O
HO
O
HO
HO
O
N_3
ppm
0
10
20
30
40
50
60
70
80
90
100
110
7
6
5
4
3
2
1
ppm

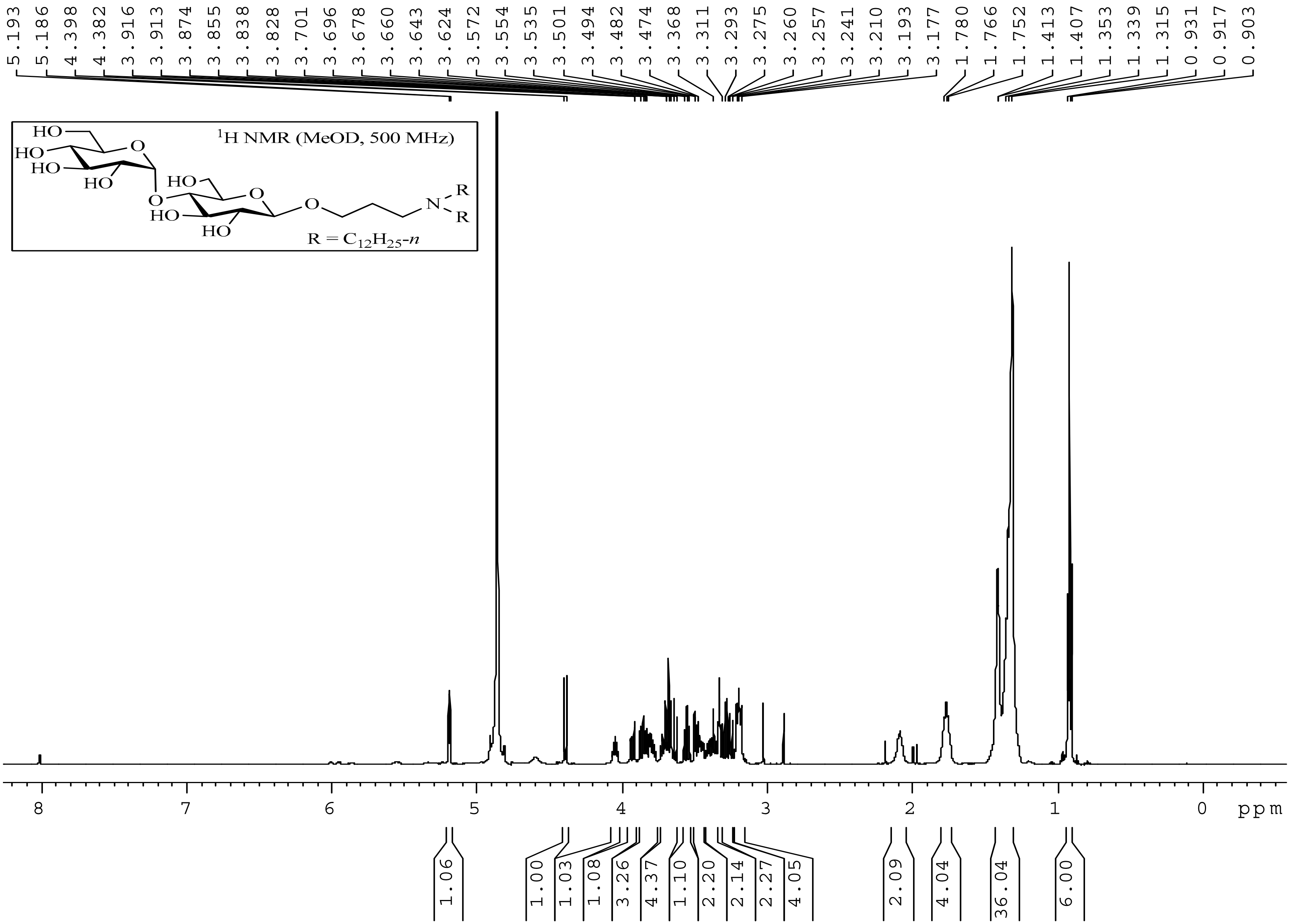

1H NMR (MeOD, 500 MHz)
R = C12H25-n
5.193 5.186 4.398 4.382 3.916 3.913 3.874 3.855 3.838 3.828 3.701 3.696 3.678 3.660 3.643 3.624 3.572 3.554 3.535 3.501 3.494 3.482 3.474 3.368 3.311 3.293 3.275 3.260 3.257 3.241 3.210 3.193 3.177 1.780 1.766 1.752 1.413 1.407 1.353 1.339 1.315 0.931 0.917 0.903
8 7 6 5 4 3 2 1 0 ppm
1.06 1.00 1.03 1.08 3.26 4.37 1.10 2.20 2.14 2.27 4.05 2.09 4.04 36.04 6.00

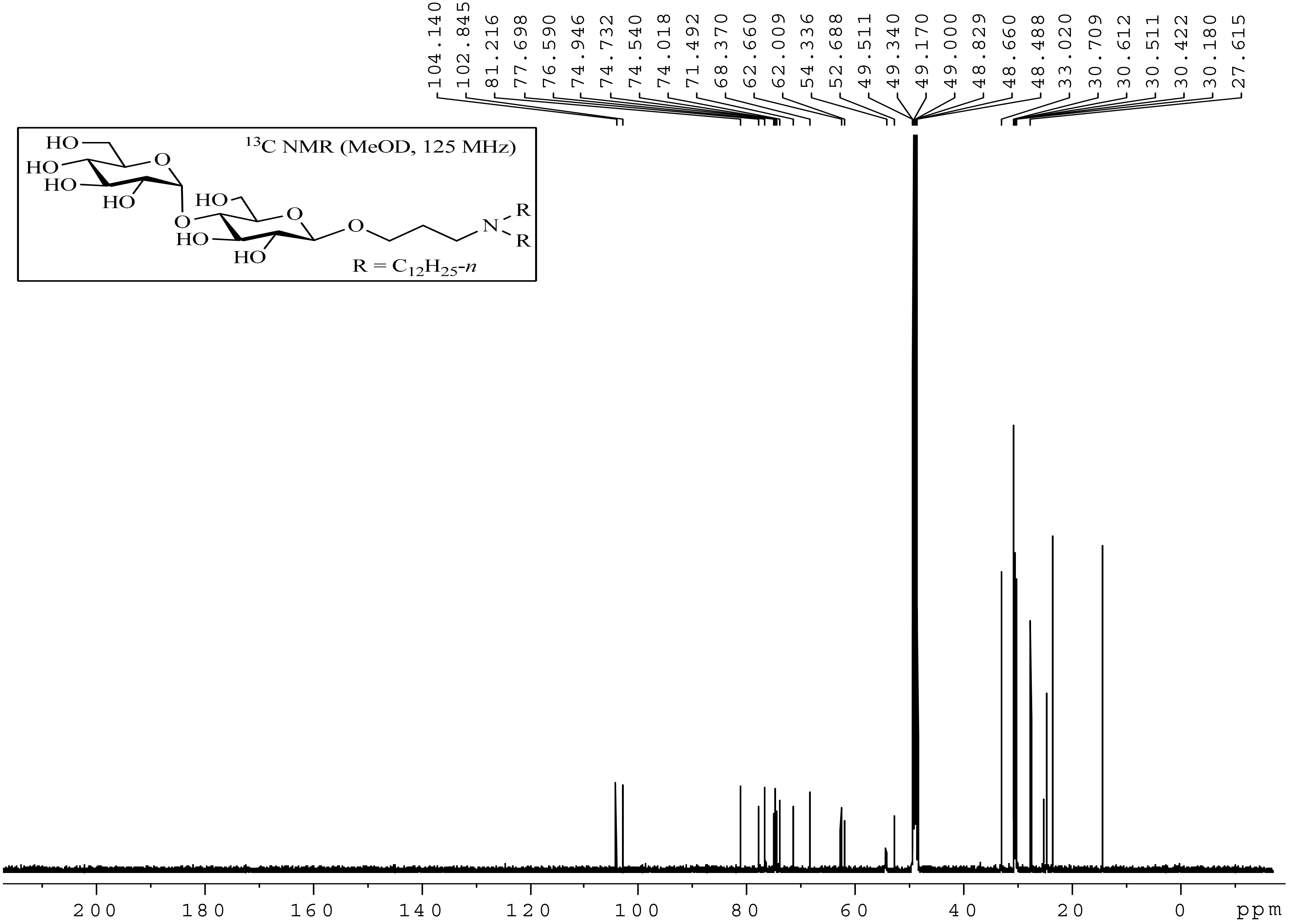

104.140
102.845
81.216
77.698
76.590
74.946
74.732
74.540
74.018
71.492
68.370
62.660
62.009
54.336
52.688
49.511
49.340
49.170
49.000
48.829
48.660
48.488
33.020
30.709
30.612
30.511
30.422
30.180
27.615
^{13}C NMR (MeOD, 125 MHz)
R = $C_{12}H_{25}$-*n*
200 180 160 140 120 100 80 60 40 20 0 ppm

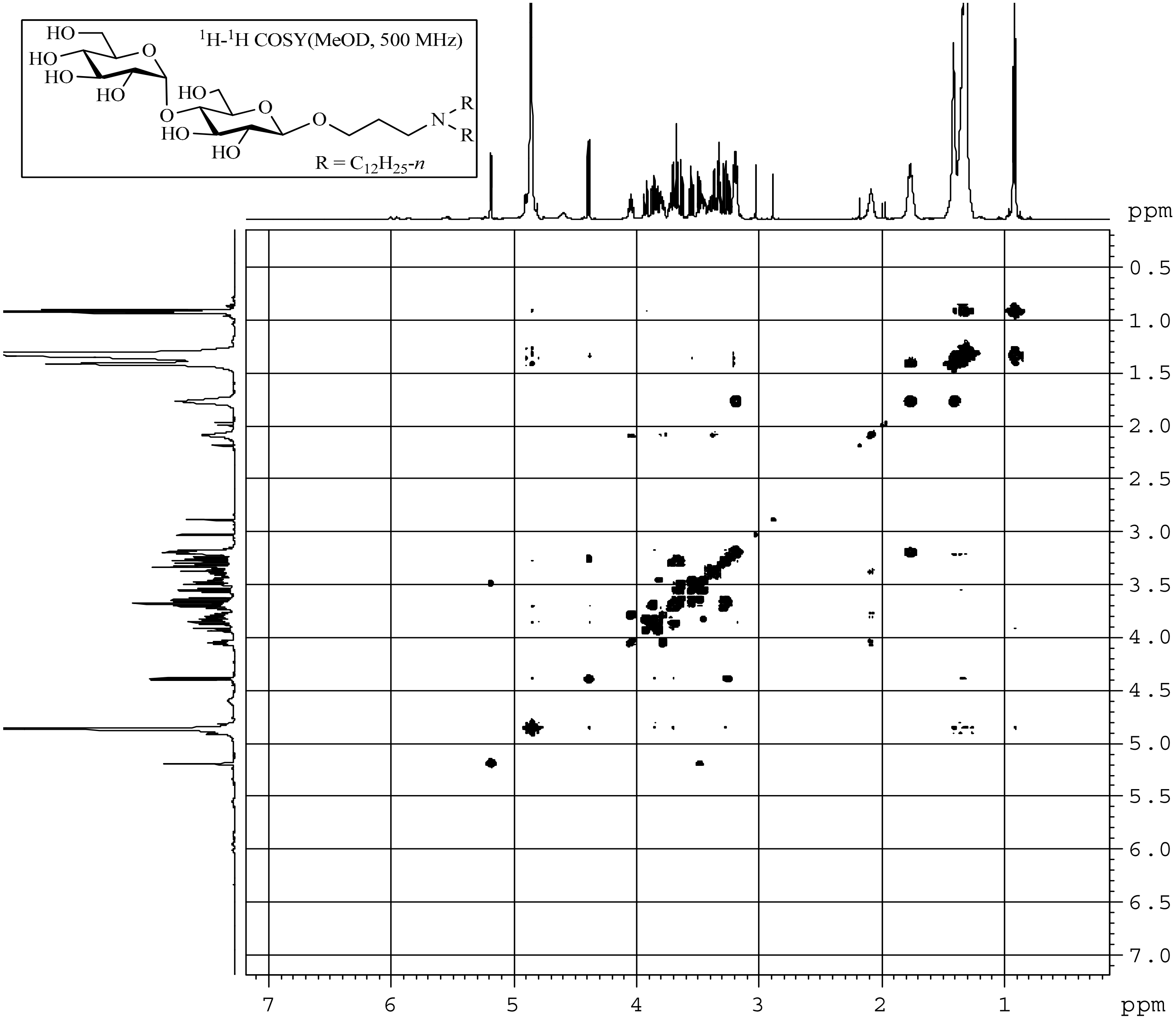
^{1}H-^{1}H COSY(MeOD, 500 MHz)
R = $C_{12}H_{25}$-*n*
ppm
0.5
1.0
1.5
2.0
2.5
3.0
3.5
4.0
4.5
5.0
5.5
6.0
6.5
7.0
7
6
5
4
3
2
1
ppm

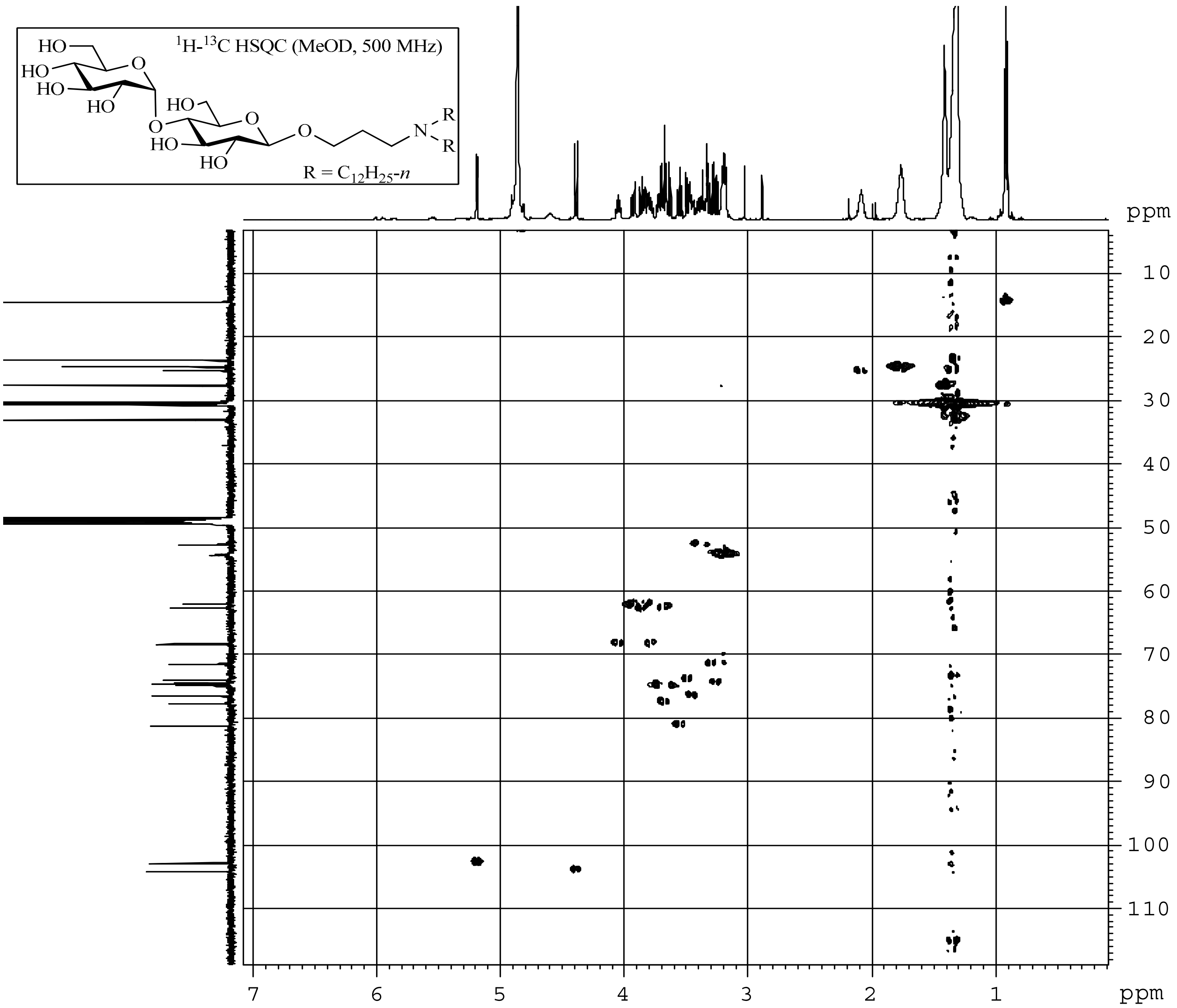
^{1}H-^{13}C HSQC (MeOD, 500 MHz)
R = $C_{12}H_{25}$-*n*
ppm
ppm

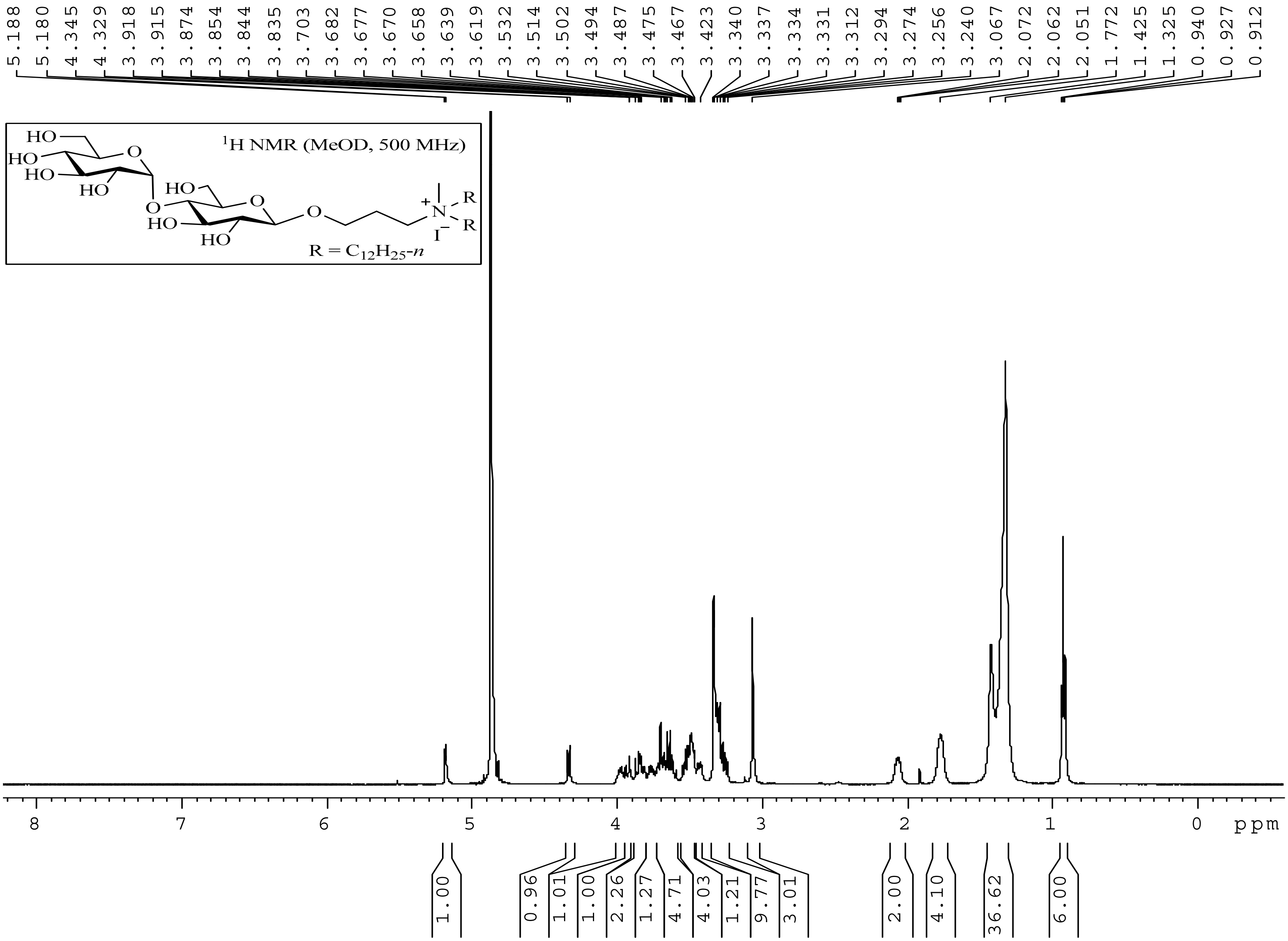
1H NMR (MeOD, 500 MHz)
R = C12H25-n
5.188 5.180 4.345 4.329 3.918 3.915 3.874 3.854 3.844 3.835 3.703 3.682 3.677 3.670 3.658 3.639 3.619 3.532 3.514 3.502 3.494 3.487 3.475 3.467 3.423 3.340 3.337 3.334 3.331 3.312 3.294 3.274 3.256 3.240 3.067 2.072 2.062 2.051 1.772 1.425 1.325 0.940 0.927 0.912
8 7 6 5 4 3 2 1 0 ppm
1.00 0.96 1.01 1.00 2.26 1.27 4.71 4.03 1.21 9.77 3.01 2.00 4.10 36.62 6.00

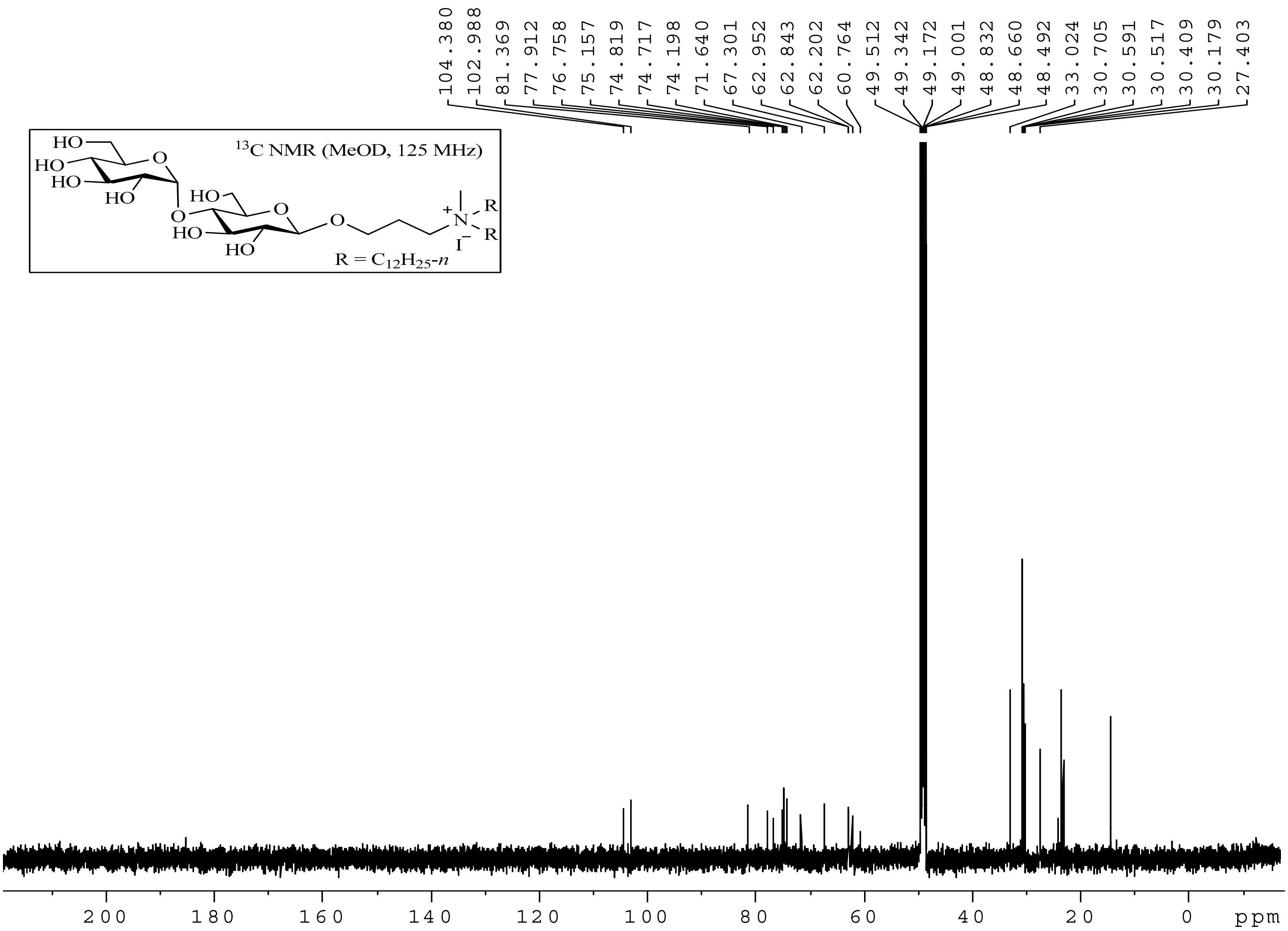
13C NMR (MeOD, 125 MHz)
R = C12H25-n
104.380
102.988
81.369
77.912
76.758
75.157
74.819
74.717
74.198
71.640
67.301
62.952
62.843
62.202
60.764
49.512
49.342
49.172
49.001
48.832
48.660
48.492
33.024
30.705
30.591
30.517
30.409
30.179
27.403
200
180
160
140
120
100
80
60
40
20
0
ppm

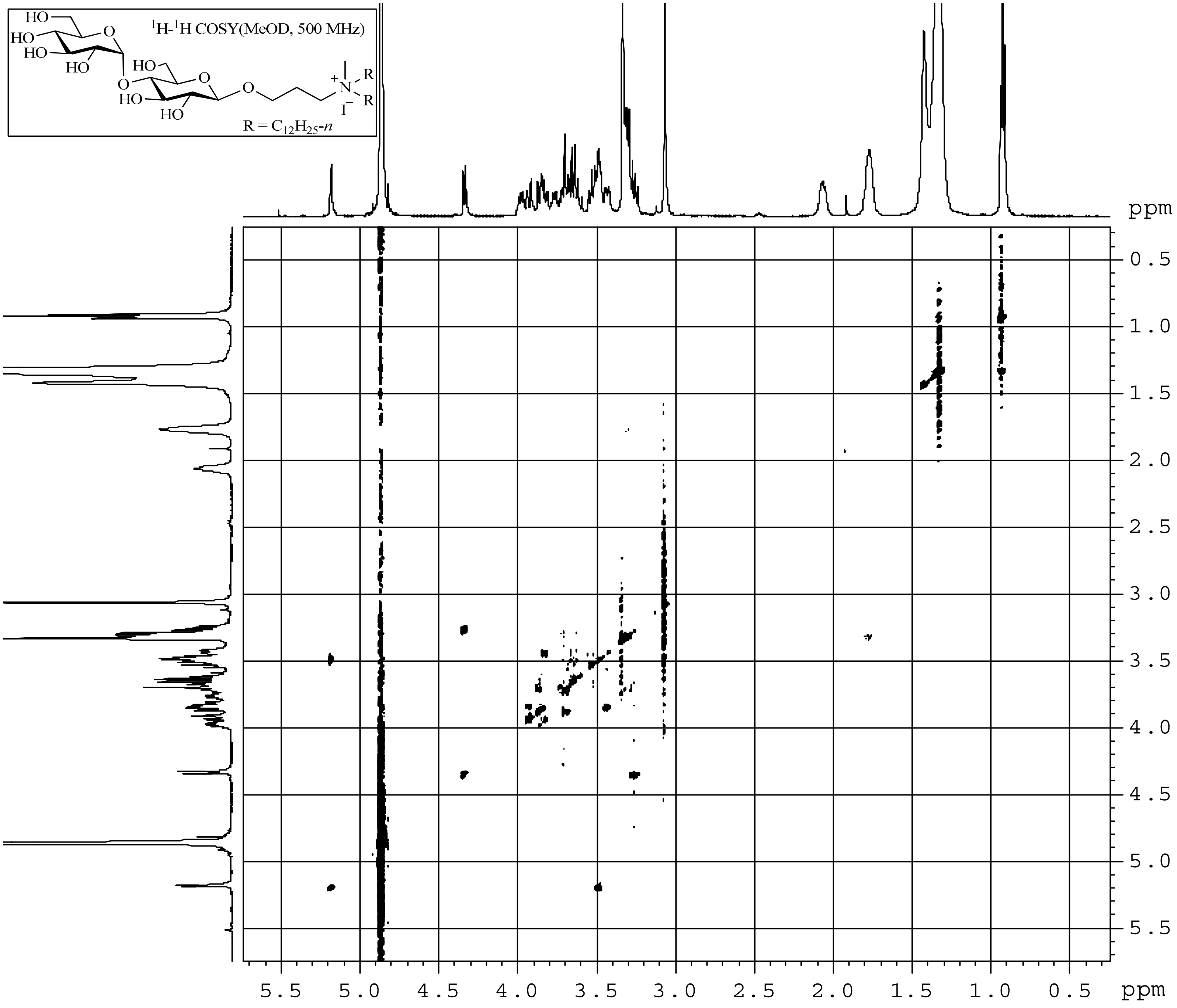

^1H-^1H COSY(MeOD, 500 MHz)
R = $C_{12}H_{25}$-*n*
ppm
5.5 5.0 4.5 4.0 3.5 3.0 2.5 2.0 1.5 1.0 0.5

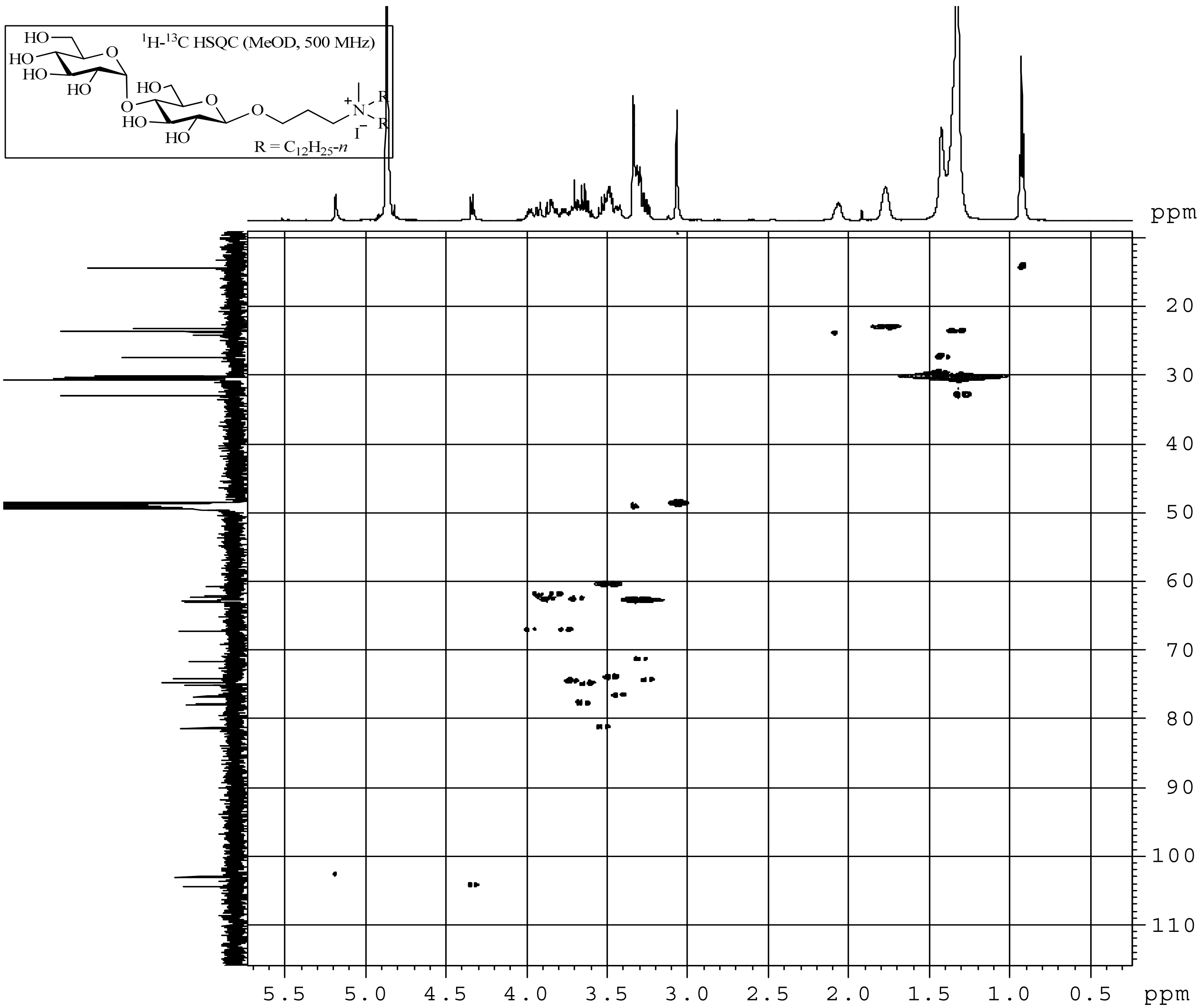

^{1}H-^{13}C HSQC (MeOD, 500 MHz)
R = $C_{12}H_{25}$-*n*
ppm

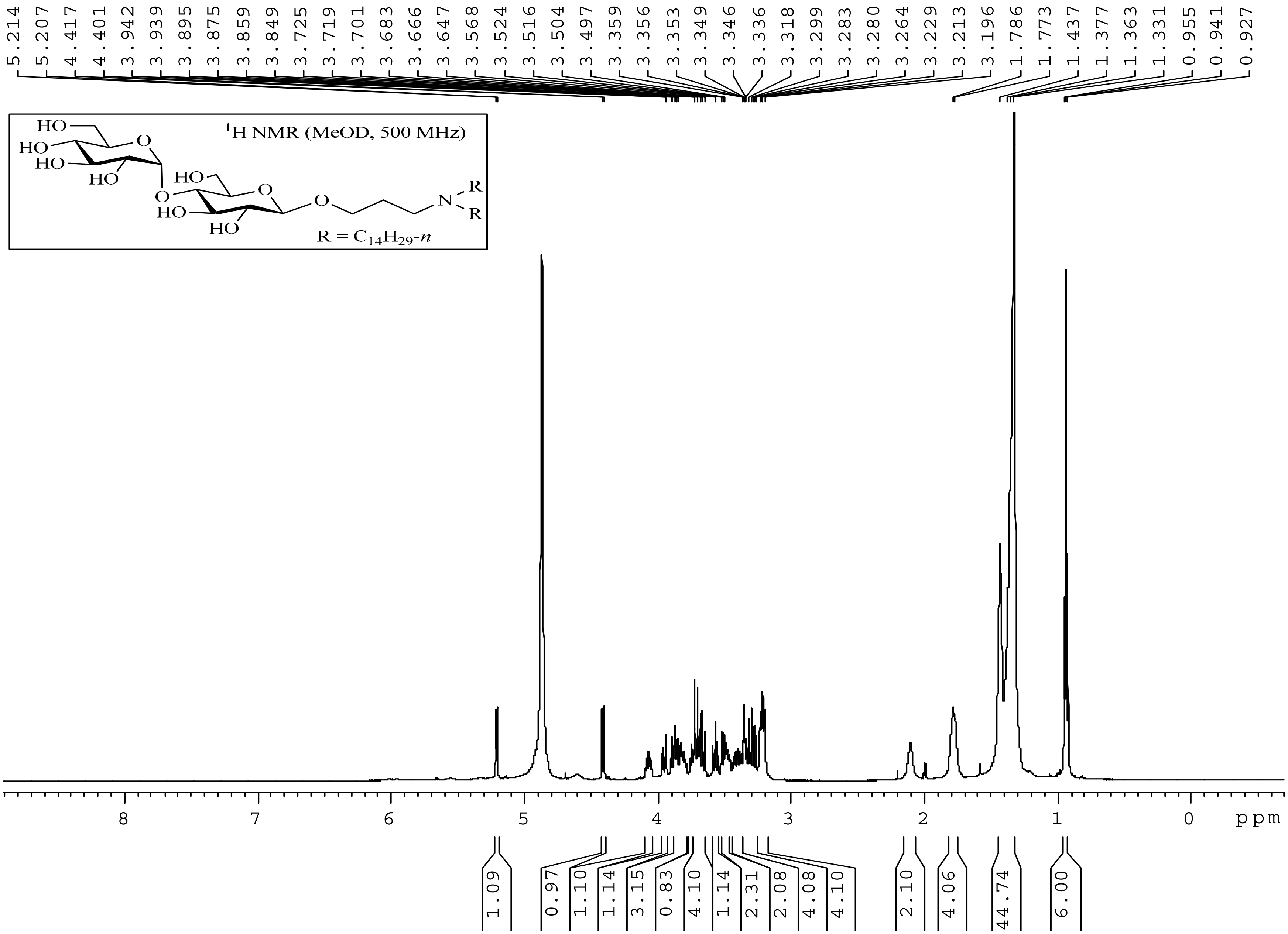
5.214
5.207
4.417
4.401
3.942
3.939
3.895
3.875
3.859
3.849
3.725
3.719
3.701
3.683
3.666
3.647
3.568
3.524
3.516
3.504
3.497
3.359
3.356
3.353
3.349
3.346
3.336
3.318
3.299
3.283
3.280
3.264
3.229
3.213
3.196
1.786
1.773
1.437
1.377
1.363
1.331
0.955
0.941
0.927
HO
HO
HO
HO
O
HO
O
HO
HO
O
O
N
R
R
1H NMR (MeOD, 500 MHz)
R = C14H29-n
8
7
6
5
4
3
2
1
0
ppm
1.09
0.97
1.10
1.14
3.15
0.83
4.10
1.14
2.31
2.08
4.08
4.10
2.10
4.06
44.74
6.00

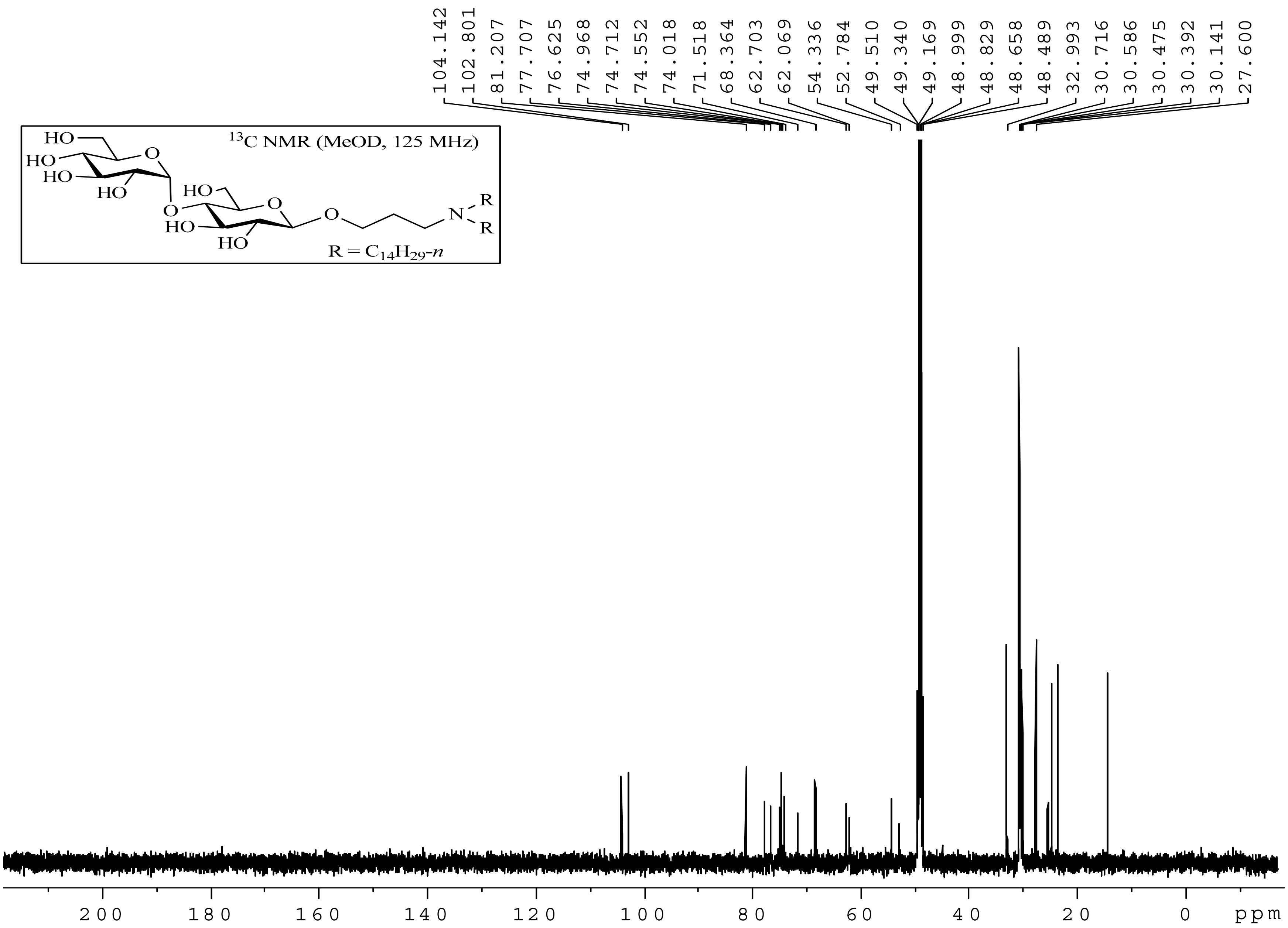

13C NMR (MeOD, 125 MHz)
R = C14H29-n
104.142
102.801
81.207
77.707
76.625
74.968
74.712
74.552
74.018
71.518
68.364
62.703
62.069
54.336
52.784
49.510
49.340
49.169
48.999
48.829
48.658
48.489
32.993
30.716
30.586
30.475
30.392
30.141
27.600
200
180
160
140
120
100
80
60
40
20
0
ppm

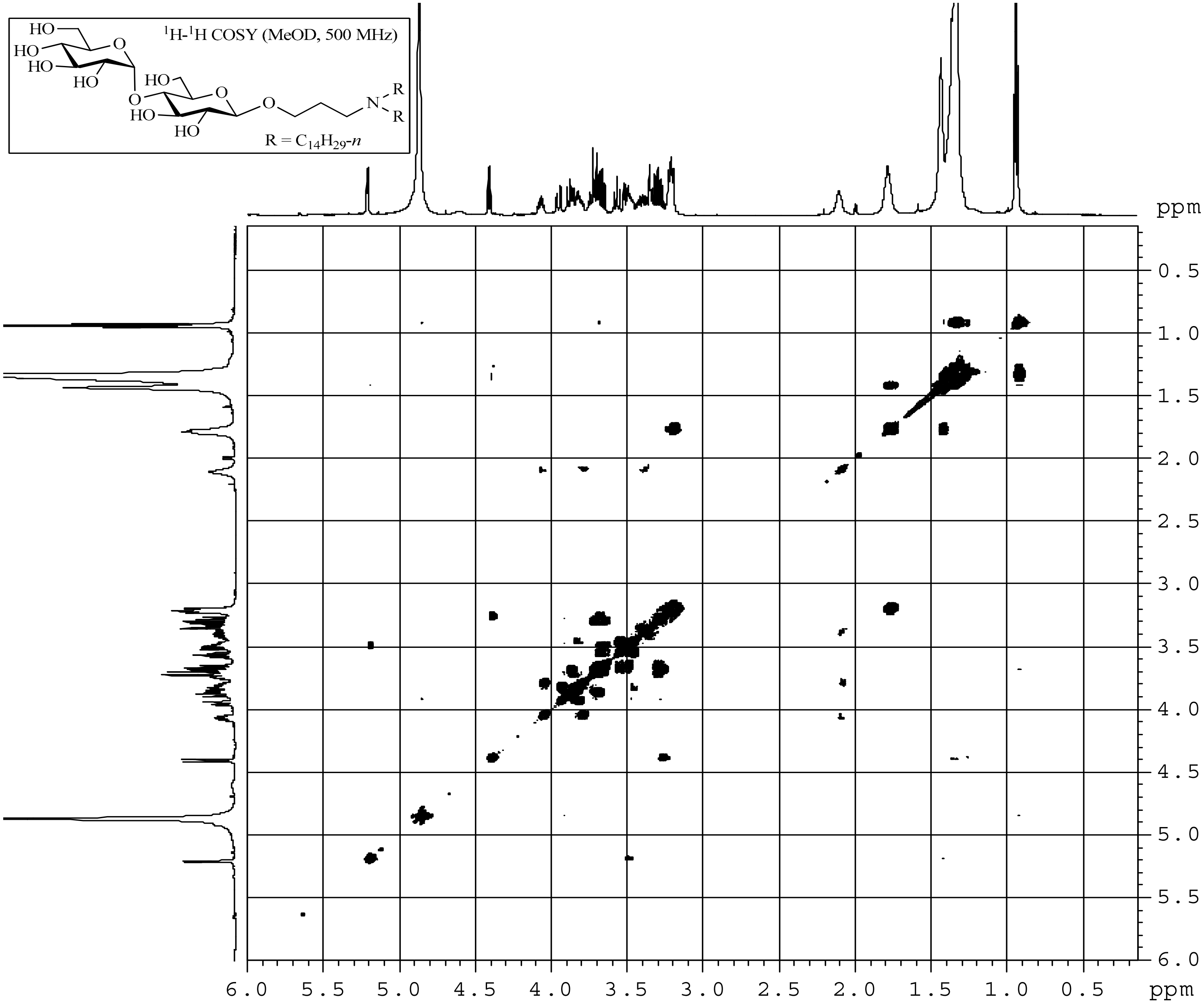

HO
HO
HO
HO
O
O
HO
O
HO
HO
O
N
R
R
R = C14H29-n
1H-1H COSY (MeOD, 500 MHz)
ppm
0.5
1.0
1.5
2.0
2.5
3.0
3.5
4.0
4.5
5.0
5.5
6.0
6.0 5.5 5.0 4.5 4.0 3.5 3.0 2.5 2.0 1.5 1.0 0.5
ppm

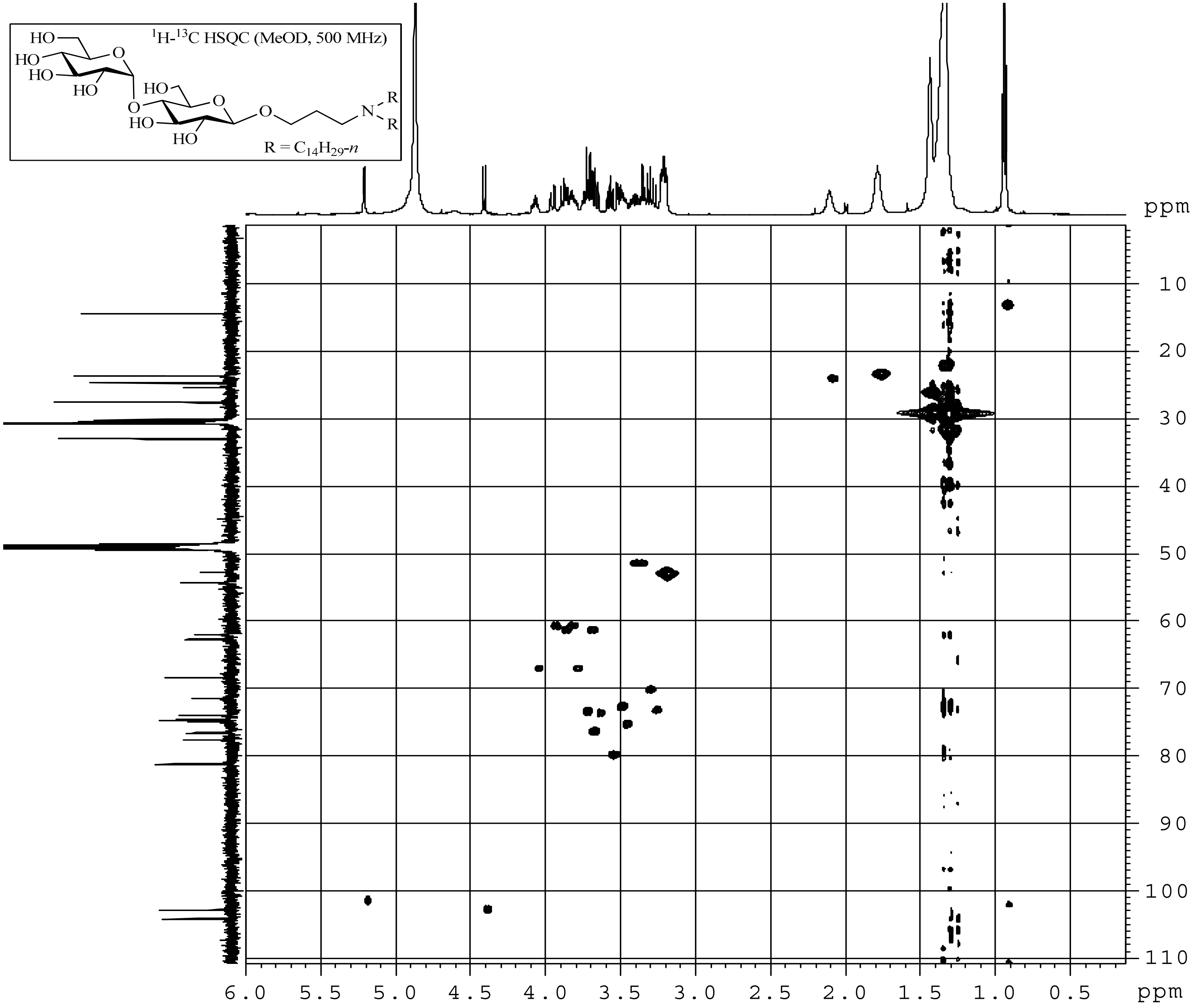

^{1}H-^{13}C HSQC (MeOD, 500 MHz)
HO
HO
HO
HO
O
O
HO
O
HO
HO
O
O
N
R
R
R = $C_{14}H_{29}$-*n*
ppm
10
20
30
40
50
60
70
80
90
100
110
6.0 5.5 5.0 4.5 4.0 3.5 3.0 2.5 2.0 1.5 1.0 0.5
ppm

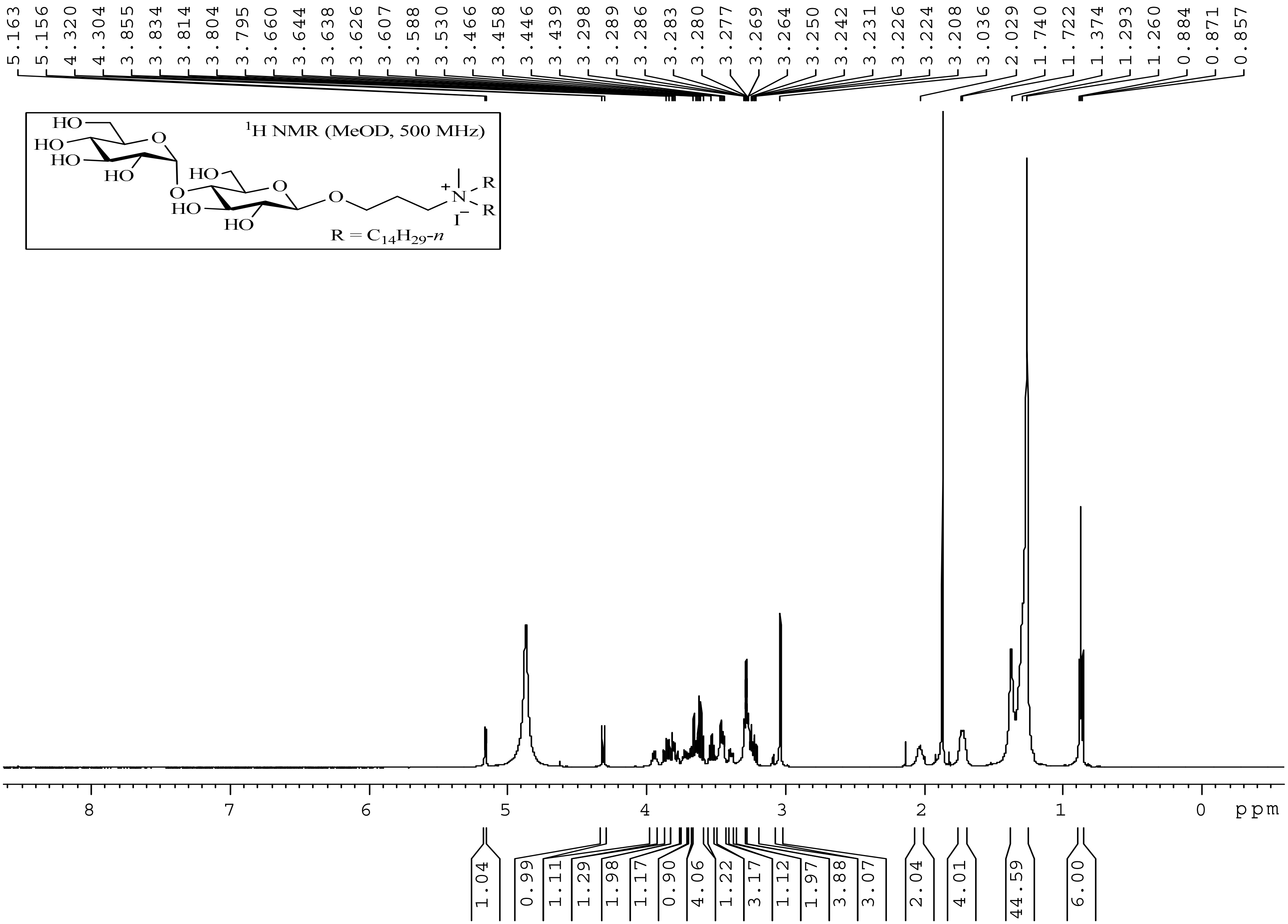

1H NMR (MeOD, 500 MHz)
R = C14H29-n
5.163 5.156 4.320 4.304 3.855 3.834 3.814 3.804 3.795 3.660 3.644 3.638 3.626 3.607 3.588 3.530 3.466 3.458 3.446 3.439 3.298 3.289 3.286 3.283 3.280 3.277 3.269 3.264 3.250 3.242 3.231 3.226 3.224 3.208 3.036 2.029 1.740 1.722 1.374 1.293 1.260 0.884 0.871 0.857
8 7 6 5 4 3 2 1 0 ppm
1.04 0.99 1.11 1.29 1.98 1.17 0.90 4.06 1.22 3.17 1.12 1.97 3.88 3.07 2.04 4.01 44.59 6.00

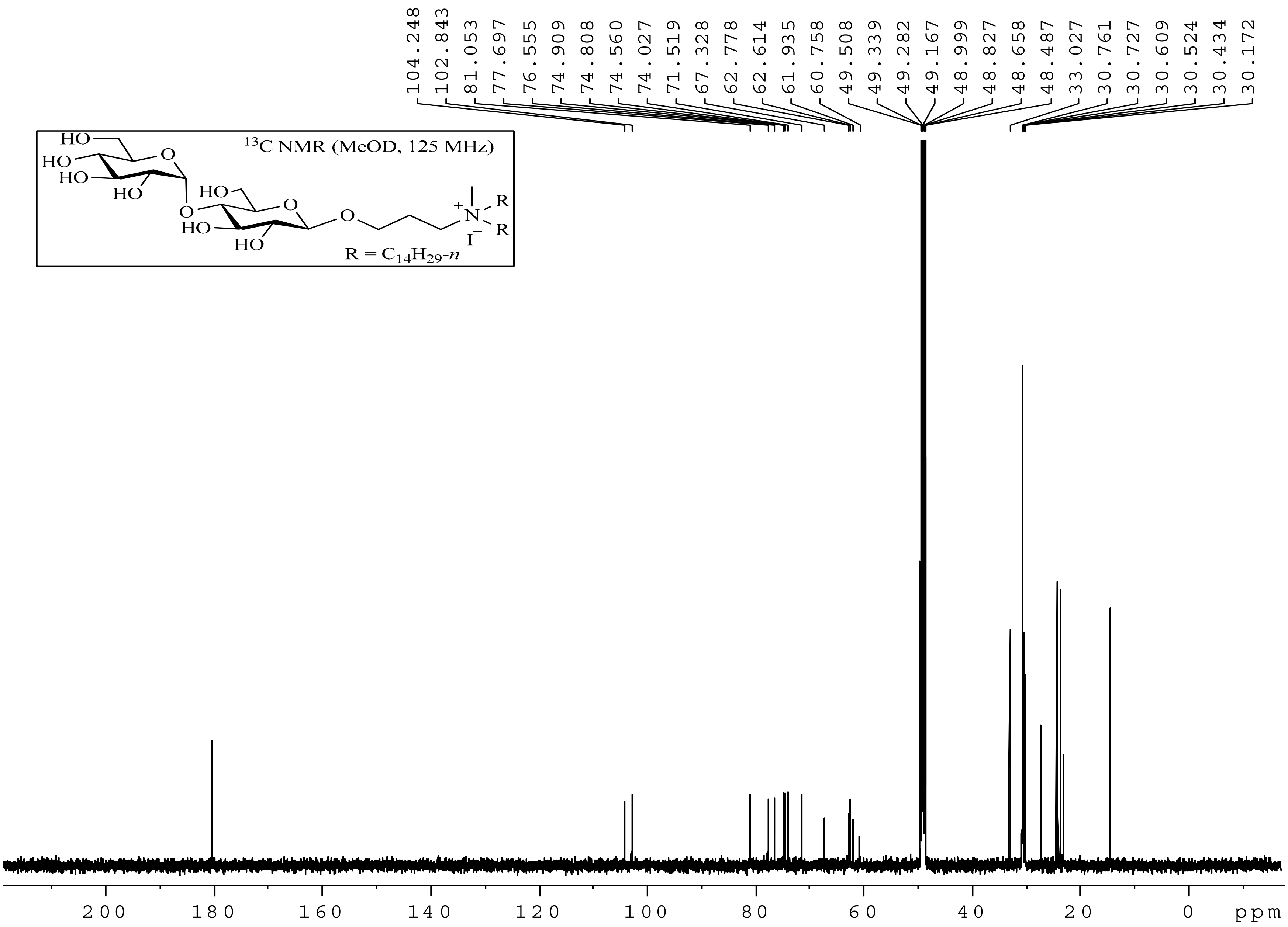
104.248
102.843
81.053
77.697
76.555
74.909
74.808
74.560
74.027
71.519
67.328
62.778
62.614
61.935
60.758
49.508
49.339
49.282
49.167
48.999
48.827
48.658
48.487
33.027
30.761
30.727
30.609
30.524
30.434
30.172
13C NMR (MeOD, 125 MHz)
R = C14H29-n
200
180
160
140
120
100
80
60
40
20
0
ppm

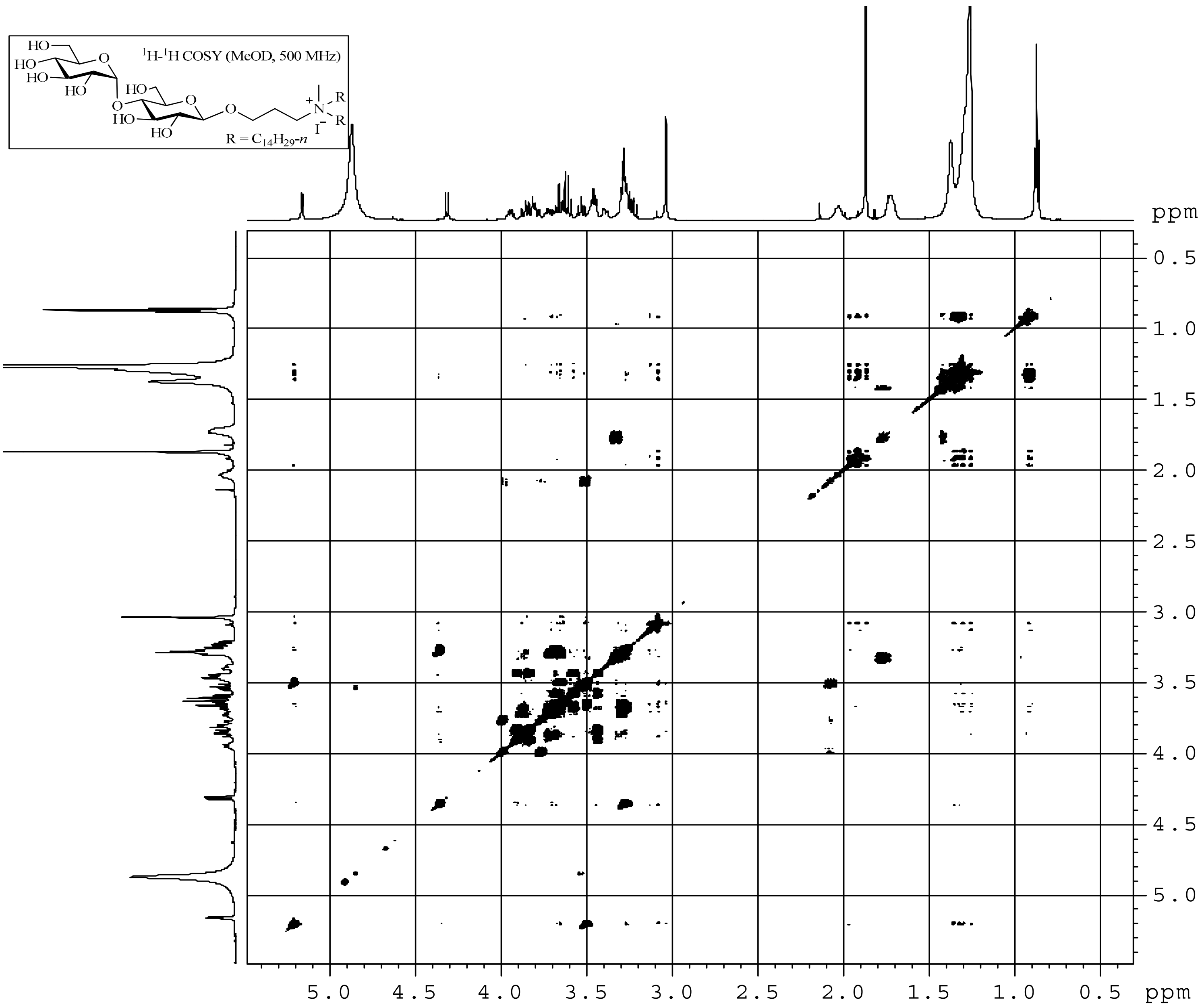

^{1}H-^{1}H COSY (MeOD, 500 MHz)
R = $C_{14}H_{29}$-*n*
ppm

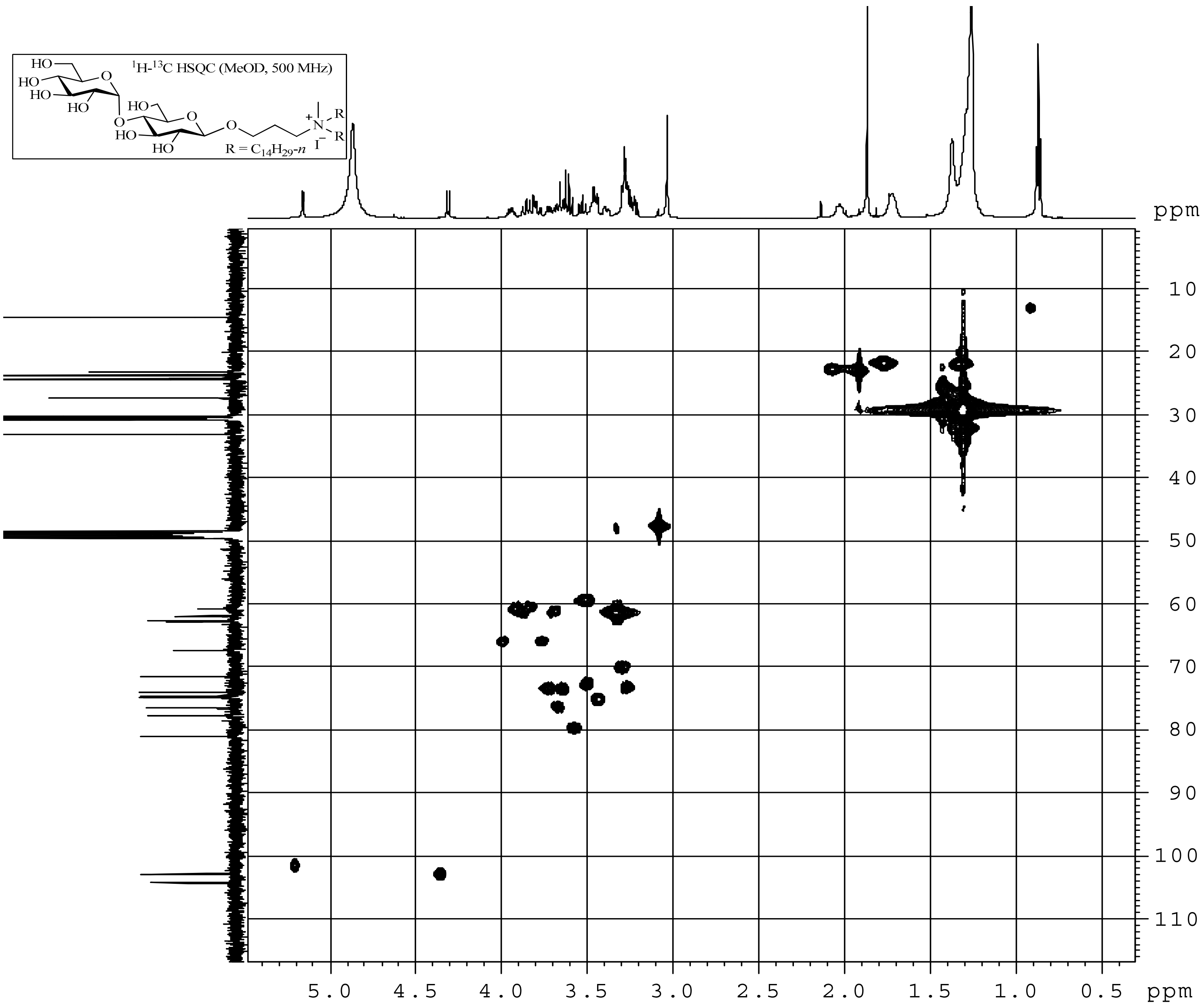

HO
HO
HO
HO
O
O
HO
O
HO
HO
HO
O
O
N
I
R
R
R = $C_{14}H_{29}$-*n*
^{1}H-^{13}C HSQC (MeOD, 500 MHz)
ppm
10
20
30
40
50
60
70
80
90
100
110
5.0
4.5
4.0
3.5
3.0
2.5
2.0
1.5
1.0
0.5
ppm

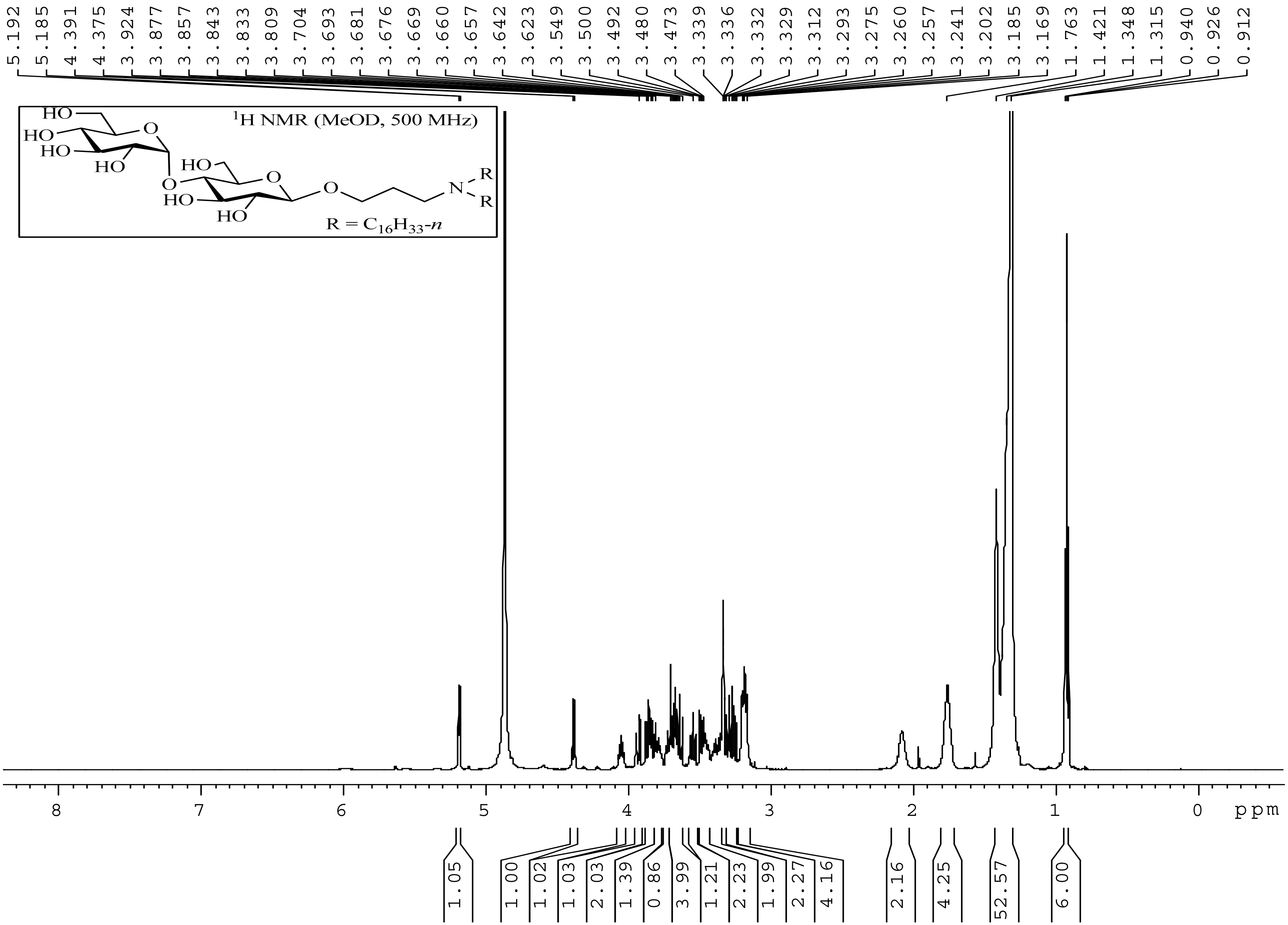
5.192
5.185
4.391
4.375
3.924
3.877
3.857
3.843
3.833
3.809
3.704
3.693
3.681
3.676
3.669
3.660
3.657
3.642
3.623
3.549
3.500
3.492
3.480
3.473
3.339
3.336
3.332
3.329
3.312
3.293
3.275
3.260
3.257
3.241
3.202
3.185
3.169
1.763
1.421
1.348
1.315
0.940
0.926
0.912
1H NMR (MeOD, 500 MHz)
R = C16H33-n
8
7
6
5
4
3
2
1
0
ppm
1.05
1.00
1.02
1.03
2.03
1.39
0.86
3.99
1.21
2.23
1.99
2.27
4.16
2.16
4.25
52.57
6.00

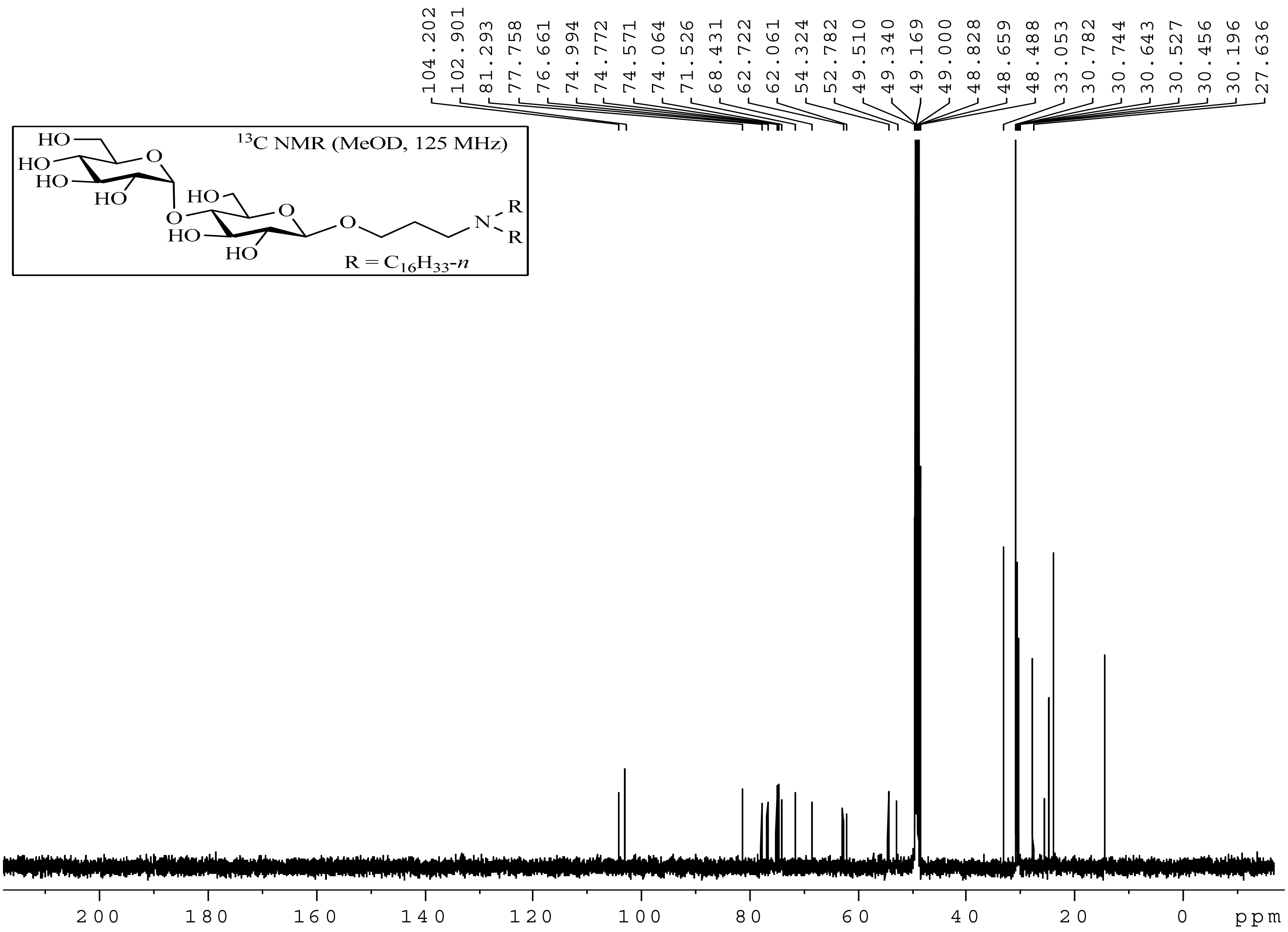

^{13}C NMR (MeOD, 125 MHz)
R = $C_{16}H_{33}$-n
104.202
102.901
81.293
77.758
76.661
74.994
74.772
74.571
74.064
71.526
68.431
62.722
62.061
54.324
52.782
49.510
49.340
49.169
49.000
48.828
48.659
48.488
33.053
30.782
30.744
30.643
30.527
30.456
30.196
27.636
200 180 160 140 120 100 80 60 40 20 0 ppm

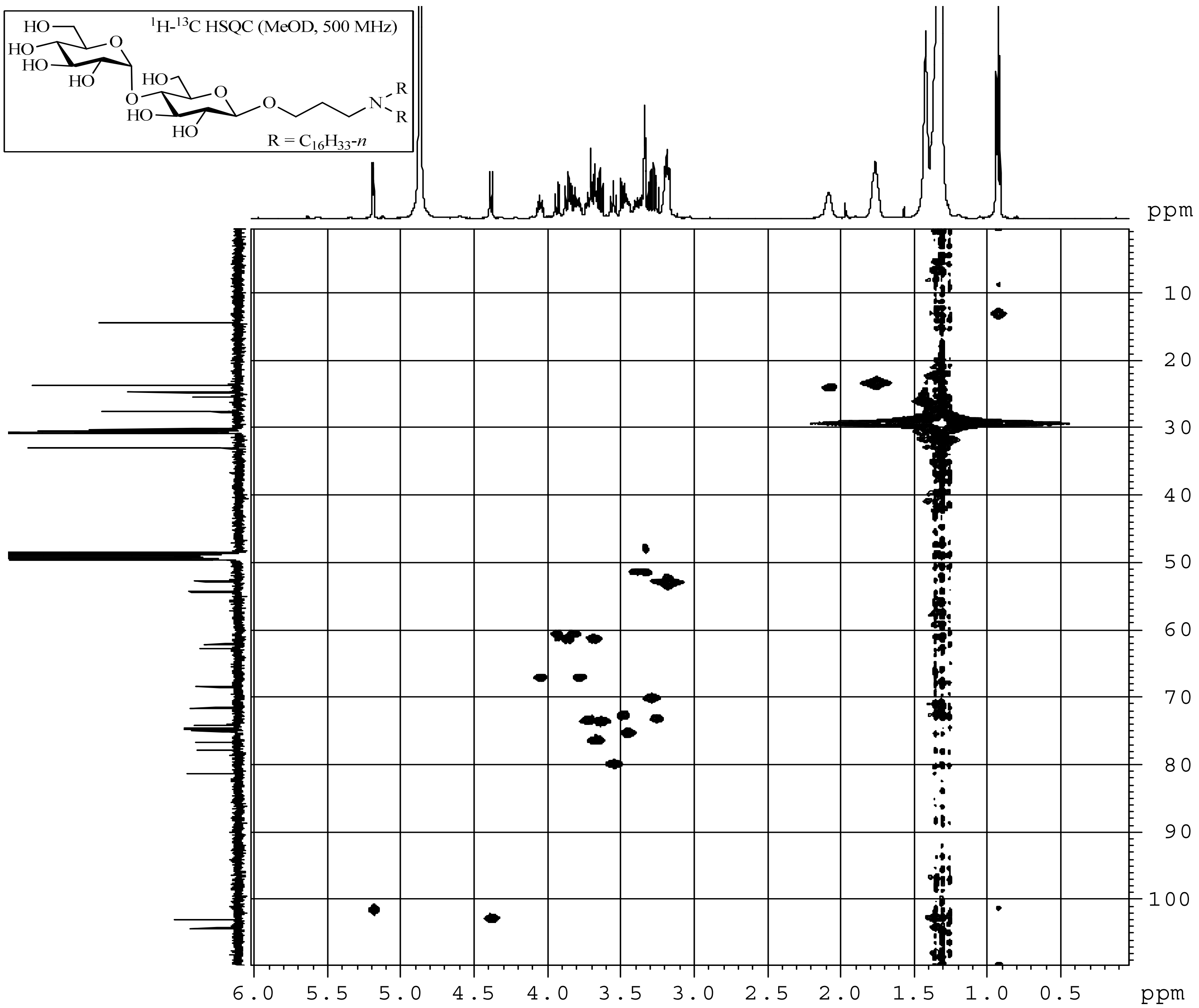

HO
HO
HO
HO
O
O
HO
O
HO
HO
O
N
R
R
R = $C_{16}H_{33}$-*n*
^{1}H-^{13}C HSQC (MeOD, 500 MHz)
ppm
10
20
30
40
50
60
70
80
90
100
6.0 5.5 5.0 4.5 4.0 3.5 3.0 2.5 2.0 1.5 1.0 0.5
ppm

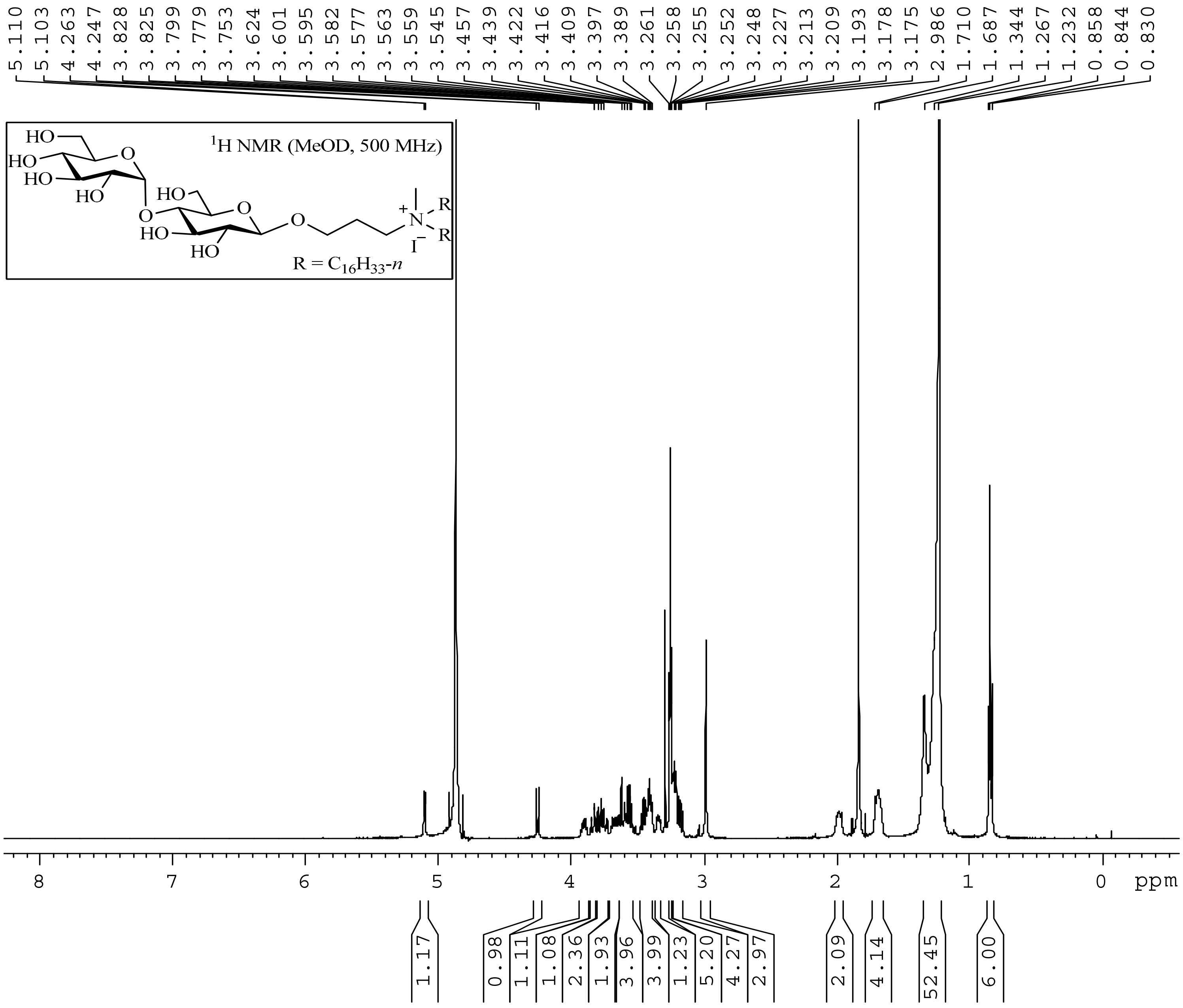

5.110 5.103 4.263 4.247 3.828 3.825 3.799 3.779 3.753 3.624 3.601 3.595 3.582 3.577 3.563 3.559 3.545 3.457 3.439 3.422 3.416 3.409 3.397 3.389 3.261 3.258 3.255 3.252 3.248 3.227 3.213 3.209 3.193 3.178 3.175 2.986 1.710 1.687 1.344 1.267 1.232 0.858 0.844 0.830
1H NMR (MeOD, 500 MHz)
HO
O
HO
HO
HO
HO
O
O
HO
HO
O
N
R
R
I
R = C16H33-n
8 7 6 5 4 3 2 1 0 ppm
1.17 0.98 1.11 1.08 2.36 1.93 3.96 3.99 1.23 5.20 4.27 2.97 2.09 4.14 52.45 6.00

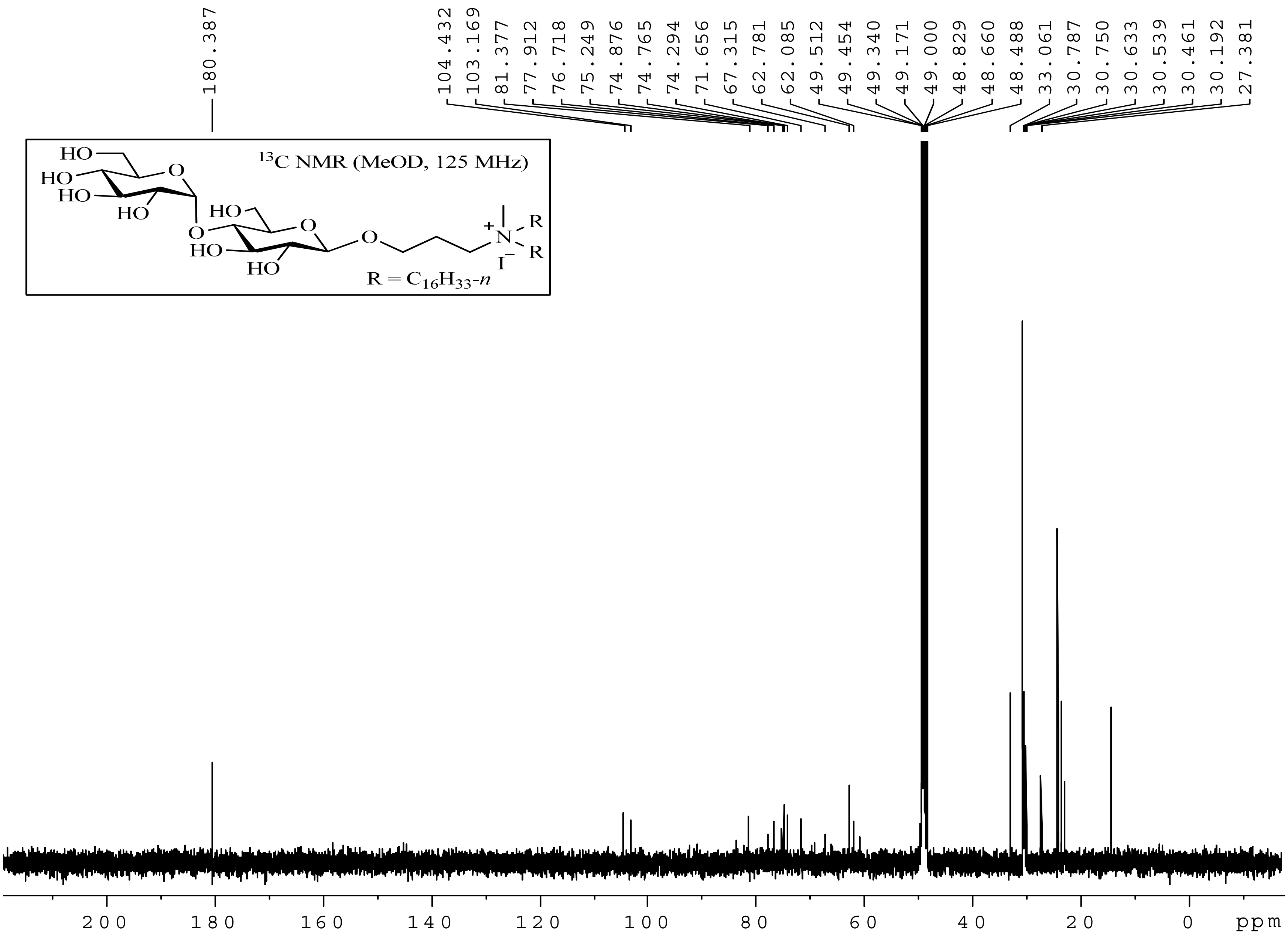
180.387
104.432
103.169
81.377
77.912
76.718
75.249
74.876
74.765
74.294
71.656
67.315
62.781
62.085
49.512
49.454
49.340
49.171
49.000
48.829
48.660
48.488
33.061
30.787
30.750
30.633
30.539
30.461
30.192
27.381
13C NMR (MeOD, 125 MHz)
HO
HO
HO
HO
O
O
HO
O
HO
HO
O
N
I
R
R
R = C16H33-n
200
180
160
140
120
100
80
60
40
20
0
ppm

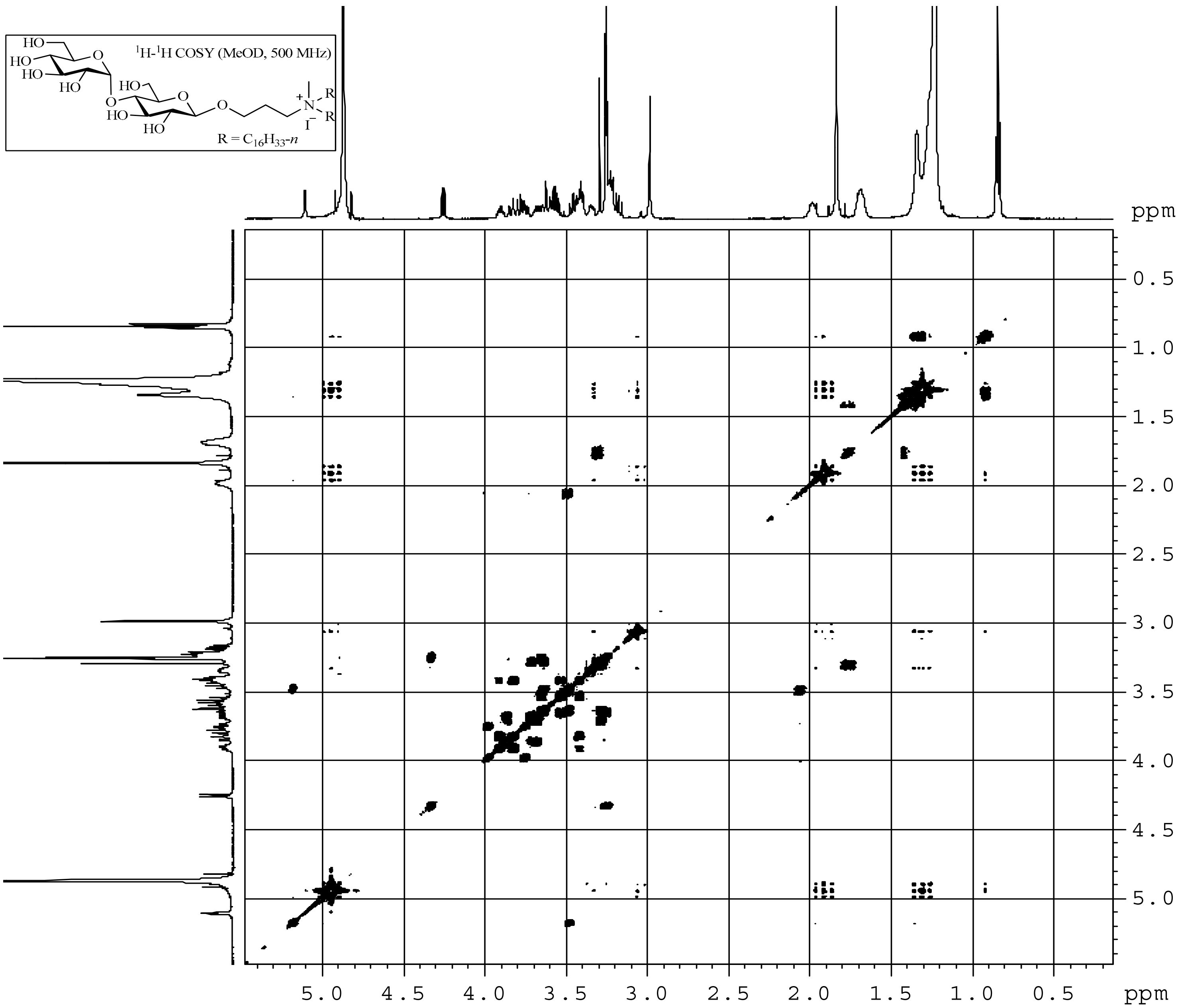

^{1}H-^{1}H COSY (MeOD, 500 MHz)
R = $C_{16}H_{33}$-n
ppm
0.5
1.0
1.5
2.0
2.5
3.0
3.5
4.0
4.5
5.0
ppm
5.0
4.5
4.0
3.5
3.0
2.5
2.0
1.5
1.0
0.5

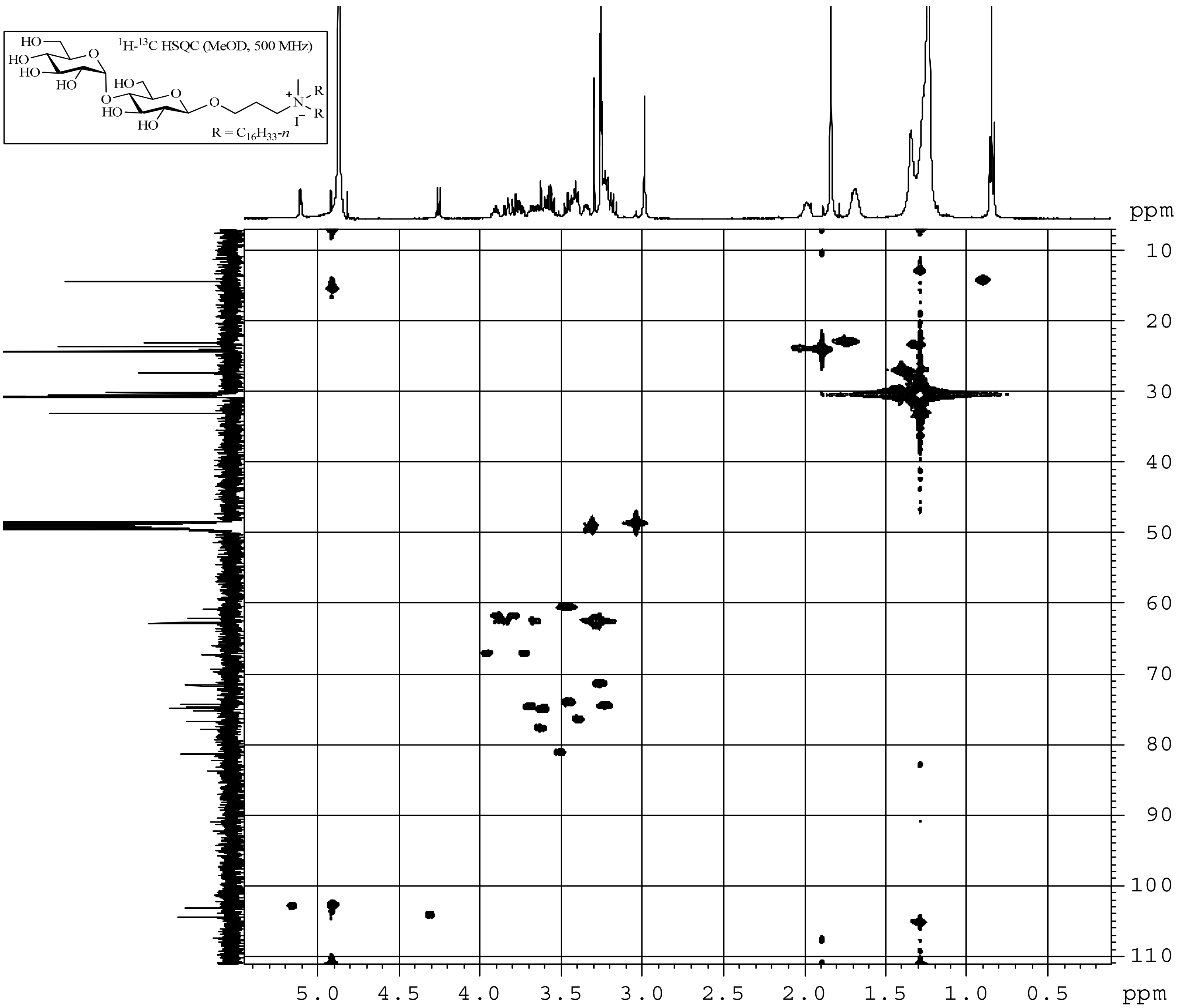
^{1}H-^{13}C HSQC (MeOD, 500 MHz)
R = $C_{16}H_{33}$-n
ppm
10
20
30
40
50
60
70
80
90
100
110
5.0
4.5
4.0
3.5
3.0
2.5
2.0
1.5
1.0
0.5
ppm
